# Selective Nitrate Transmembrane Transport Through Adaptive Weak C─H Bonding Cyanostilbene Water Channels

**DOI:** 10.1002/anie.4918548

**Published:** 2026-05-11

**Authors:** Ioan Stroia, Dan‐Dan Su, Yuhao Li, Niculina Hadade, Ion Grosu, Arie van der Lee, Mihail Barboiu

**Affiliations:** ^1^ Institut Européen des Membrane Adaptive Supramolecular Nanosystems Group University of Montpellier ENSCM CNRS Montpellier France; ^2^ Supramolecular Organic and Organometallic Chemistry Center (SOOMCC) Babes‐Bolyai University Cluj‐Napoca Romania

**Keywords:** anion transport, artificial water channels, CH donors, nitrate‐selective transport, self‐assembled channels

## Abstract

Transmembrane water transport strongly depends on how dynamic, translocating water clusters are stabilized within hydrogen‐bonding (HB) channels. Both structural order and short‐lived disorder of transient channels play important roles in ion and water translocation. Strong HB can result in tight water binding and reduced mobility. We hypothesize that weaker HB binding sites reduce water friction, thereby enhancing water permeation while suppressing ion transport due to their unmet dehydration requirements within membrane. Herein, we show that weak HB CH donor cyanostilbenes promote water transport through adaptive, less‐ordered water channels within the lipid bilayer, with water translocation efficiency depending in part, on the strength of the CH donor sites. Fine‐tuning both CH donor strength and binding geometry enables modulation of anion transport selectivity, ranging from moderate NO_3_^−^ over Cl^−^ selectivity to highly NO_3_^−^‐selective transport and ultimately to exclusive water transport. For instance, we achieved over 200‐fold selectivity for NO_3_^−^ over Cl^−^ and ∼100‐fold selectivity for NO_3_^−^ over Br^−^, while some systems showed no detectable Cl^−^ or Br^−^ transport yet retaining strong NO_3_^−^ transport activity. This work represents a step toward adaptive transmembrane water channels and highlights the potential of HB CH donor channels for water translocation and for increased NO_3_^−^/ Cl^−^ selectivity.

## Graphical abstract

Here we demonstrate that weak HB CH donor cyanostilbenes promote water/ion transport through their ability to form less‐ordered transient water channels within the lipid bilayer with increased water permeability and NO_3_^−^/ Cl^−^ selectivity.

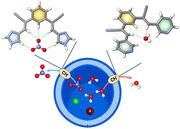

## 1 Introduction

Transmembrane transport of physiologically relevant species is of fundamental importance for regulating cellular metabolism, pH and osmotic balance, signaling [1]. Nature employs a wide range of specialized protein channels to control the passage of different solutes through the membrane, either passively or actively, according to physiological demands required to maintain homeostasis [2, 3]. Mimicking these natural channels with simpler artificial transporters (channels or carriers) is therefore of great importance, not only for biomedical applications such as the treatment of channelopathies [4, 5], but also for separation technologies, offering significant advantages in terms of processability, stability, and scalability [6].

Artificial water channels (AWCs) [7], are designed to mimic Aquaporins (AQPs), the natural channel proteins that enable selective water translocation to dissipate the osmotic pressure gradient across the cell membrane [8, 9]. While AQPs achieve highly efficient water transport with almost complete rejection of ions and protons [8], artificial analogues have emerged as innovative solutions to enable large‐scale applications (e.g., for efficient industrial seawater desalination) [10]. The field of AWCs has been gradually developed, with relatively few active compounds reported to date [10, 11, 12]. Imidazole, I‐quartet [11, 13, 14, 15], foldamers [16], pillar[5]arenes [17, 18, 19, 20, 21], hydroxy‐channels [22, 23], peptide‐pillar[4]arenes [24] are landmark examples of AWCs, I‐quartets being successfully incorporated into polymeric membranes used in water desalination processes [25, 26]. The mechanisms of water transport through AWCs are related to structural behaviors and dynamics of water clusters within the channels which control the HB connecting the water molecules between them or with the channel wall, depending on the functional nature of the channel. We previously showed *via* simulation approaches that strong Imidazole‐water HB within I‐quartet form well‐organized and structured channels for water permeation, somewhat reminiscent of AQPs, [11, 13, 14, 15] but also more dynamic, transient emerging sponge‐like membrane perturbations that can also enable water permeation [27].

Meanwhile, artificial anion transporters (channels and carriers) have enjoyed considerably greater attention. Numerous anion transporters, predominantly chloride carriers, have been developed [28, 29, 30, 31, 32], driven by the extensive groundwork in anion recognition chemistry [33] and by their promising applications in channel replacement therapy and cancer treatment [4, 34, 35, 36]. Although chloride is one of the most abundant anions in living organisms, other anions such as bicarbonate [37], sulphate [38], phosphate [39, 40, 41], and nitrate [42, 43] also play essential physiological roles, and their transmembrane transport is of crucial importance. Although AQPs are primarily transporting water, AQP6 has been shown to facilitate selective nitrate translocation, with $P_{N{O_{3}}^{-}}/P_{Cl^{-}}>9$ [44, 45, 46]. While significant efforts have been devoted to developing bicarbonate‐selective transporters [47, 48], as well as systems tailored to compensate for the high dehydration energies of sulphate [49] and phosphate [50], nitrate‐selective artificial transport has comparatively received less attention. Indeed, most designed chloride transporters also mediate nitrate transport, with two general scenarios typically observed: (i) selectivity follows the Hofmeister series, where transporters preferentially translocate more chaotropic nitrate anions [51, 52, 53] or (ii) selectivity follows the anti‐Hofmeister series, with transporters favoring chloride over chaotropic oxoanions [54, 55]. In contrast, achieving nitrate‐selective transport through the design of specific cavities for nitrate binding remains challenging, as typically such cavities also strongly bind chloride. For instance, calix[4]pyrroles, which bind nitrate through combined HB and anion–π interactions, display only a modest $S_{N{O_{3}}^{-}}{/}_{Cl^{-}}\approx 2$ [56]. Another possible strategy for achieving nitrate‐selective transport is to exploit its higher lipophilicity relative to chloride and to fine‐tune the strength of the hydrogen‐bond donors, thereby enabling nitrate, but not chloride transport. Theoretically, this can be achieved by employing weaker HB donors, such as CH groups, which are insufficient to overcome the high dehydration energy of chloride (thus preventing its strong binding and translocation), yet still effective for nitrate owing to its lower dehydration energy and greater lipophilicity.

**FIGURE 1:**
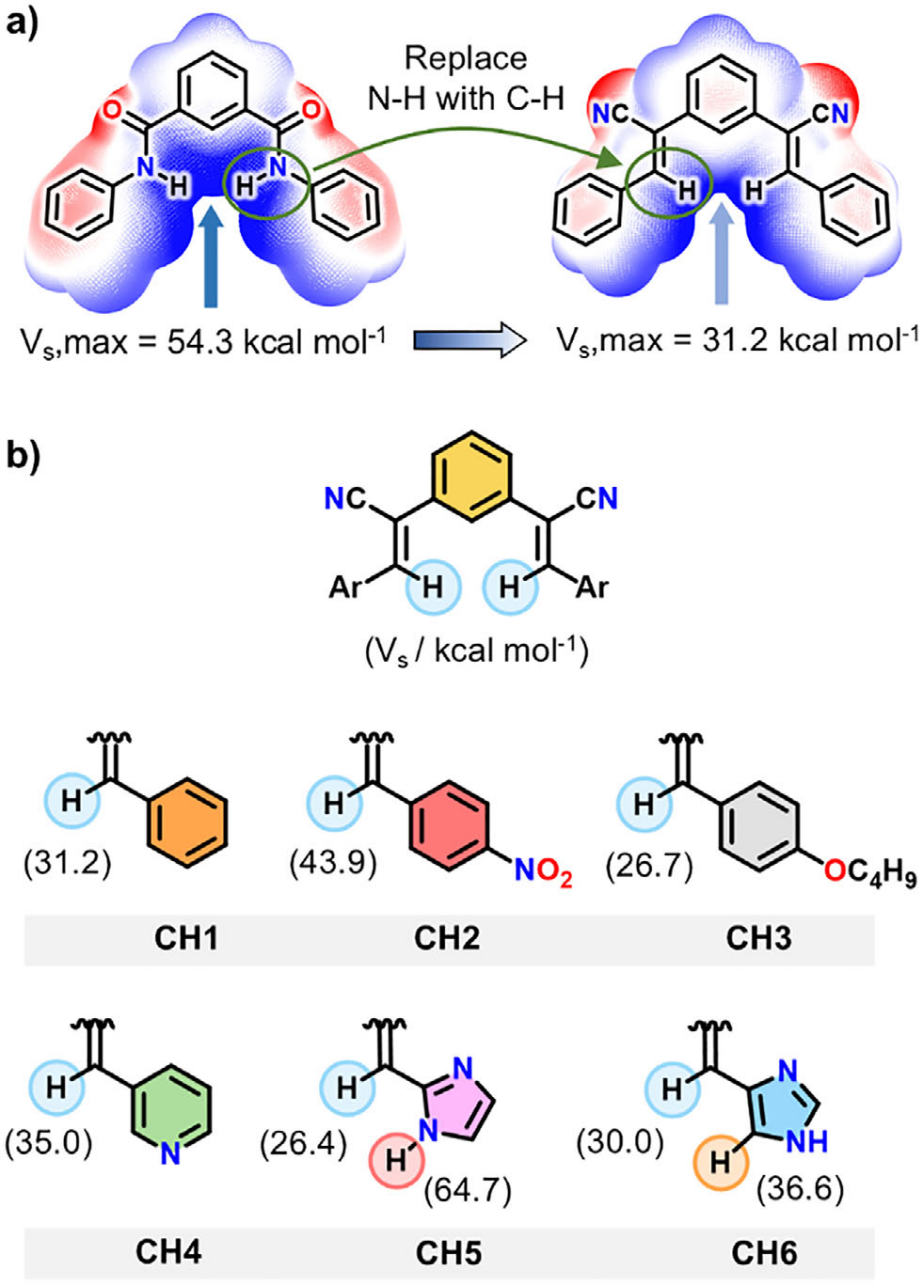
Design strategy of low‐binding‐energy cyanostilbene CH donor transporters: (a) Weakening the HB donors by conceptually replacing NH with CH binding sites, and (b) fine‐tuning the strength and the geometry of the binding sites by playing with the grafted aromatic cores. The value of the electrostatic potential (Vs, kcal mol^−1^) at the binding site (related to the strength of the anion binding power) [52] are also given.

Herein, we designed a series of simple cyanostilbene‐based CH‐donor podands (**CH1‐CH6**, Figure 1), tuning both CH donor HB strength and binding site geometry, two key determinants of anion/water translocation. Indeed, we found that weakening HB donor strength markedly reduces Cl^−^ transport activity while preserving efficient $NO_{3^{-}}$ translocation, affording over 200‐fold $NO_{3^{-}}/Cl^{-}$ selectivity. Moreover, interestingly, the dynamic nature of the self‐assembled adaptive superstructures enables efficient water transport, owing to the ability of the CH weak binding sites to stabilize transient water cluster during translocation. During the achievement of the present work, Valkenier and coworkers have pursued an opposite strategy: Strengthening CH donors to achieve high chloride transport efficiency [57]. Talukdar and coworkers have likewise demonstrated efficient chloride transport using weak HB CH‐donor foldamers [58]. Several other weak HB CH donor anionophores including the Flood's cyanostar have also been reported [59, 60, 61]; however, with the main focus on Cl^−^ transport.

These examples, together with our findings presented here, underscore how subtle adjustments in water/ion‐binding strength and geometry can finely tune both water/anion selectivity and translocation efficacy. This highlights the enormous potential of weaker CH bonding sites to induce the formation of transient water channels and enhance transmembrane water flow by enabling more dynamic water‐channel interaction than for strong HB systems. Altogether, our findings on cyanostilbene weak HB CH‐donor podands presented here aim to inspire the development of more nitrate‐selective artificial transporters along with an original series of efficient transient AWCs.

## 2 Results and Discussion

Based on the premise that donor strength and binding‐site geometry govern anion permselectivity, we designed CH‐donor analogues of isophthalamide [62, 63] NH anionophores (Figure 1). We further tuned C─H donor strength by introducing either electron‐withdrawing NO_2_ groups (**CH2**, strengthening) or electron‐donating OC_4_H_9_ substituents (**CH3**, weakening). In addition, based on our group experience with self‐assembled dipicolinamide water U‐channels [64], we incorporated pyridinic (**CH4**) and imidazole (**CH5** and **CH6**) aromatic cores to (i) introduce hydrogen‐bond acceptors that may promote self‐assembly and facilitate water transport, and (ii) enlarge the binding cavity to enhance nitrate transport selectivity. DFT calculations and quantitative analysis of the molecular surface confirmed that indeed replacing N─H with C─H donors significantly reduces HB donor strength (see the maximum electrostatic potential on the molecular surface V_s_, max in Figure 1a). Compounds **CH1**–**CH6** were obtained by reacting 1,3‐bis (isocyanatomethyl)benzene with the corresponding aldehydes (Figure S1). All compounds were thoroughly characterized by ^1^H and ^1^ ^3^C NMR spectroscopy (see Supporting Information).

### 2.1 X‐Ray Single‐Crystal Structures

**FIGURE 2:**
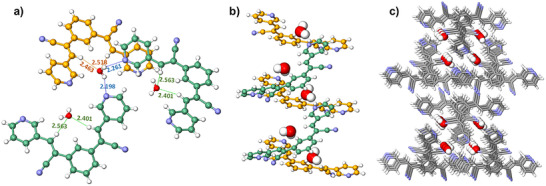
X‐ray single crystal structure of compound **CH4** co‐crystallized with water: (a) Two slightly different water‐binding modes within the CH binding sites of **CH4**; (b) Side view of their helical arrangement; (c) Top view of the molecular packing of **CH4⊃H_2_O**.

Single crystals suitable for x‐ray diffraction structure determination was obtained by slow evaporation of a concentrated THF/H_2_O (5:1, v/v) solution of **CH4**. The crystal structure reveals water binding within the podand cavity through CH···OH_2_ interactions in two different modes. Specifically, (i) water is H‐bonded almost symmetrically by the two CH donors (CH···OH_2_ distances of 2.46 and 2.52 Å), and (ii) water oriented preferentially toward one CH donor (CH···OH_2_ distances of 2.40 and 2.56 Å). These orientations are driven by the apical and basal H‐bond acceptor **CH4** molecules (i.e., through their pyridinic nitrogen, Figure 2a), generating a directional helical assembly of alternating **CH4⊃H2O** (Figure 2b).

Importantly, water templating channels with a diameter of ∼5 Å are observed (Figure 2c), with wire breaking induced by the cyano (CN) groups, which is beneficial for proton exclusion (i.e., disruption of the Grotthus proton transport mechanism, vide infra). Notably, the π–π stacking and hydrophobic interactions contribute to aggregate stabilization in the solid state and likely within the membrane, as further confirmed by MD simulations described below.

### 2.2 Water Transmembrane Transport Experiments

**FIGURE 3:**
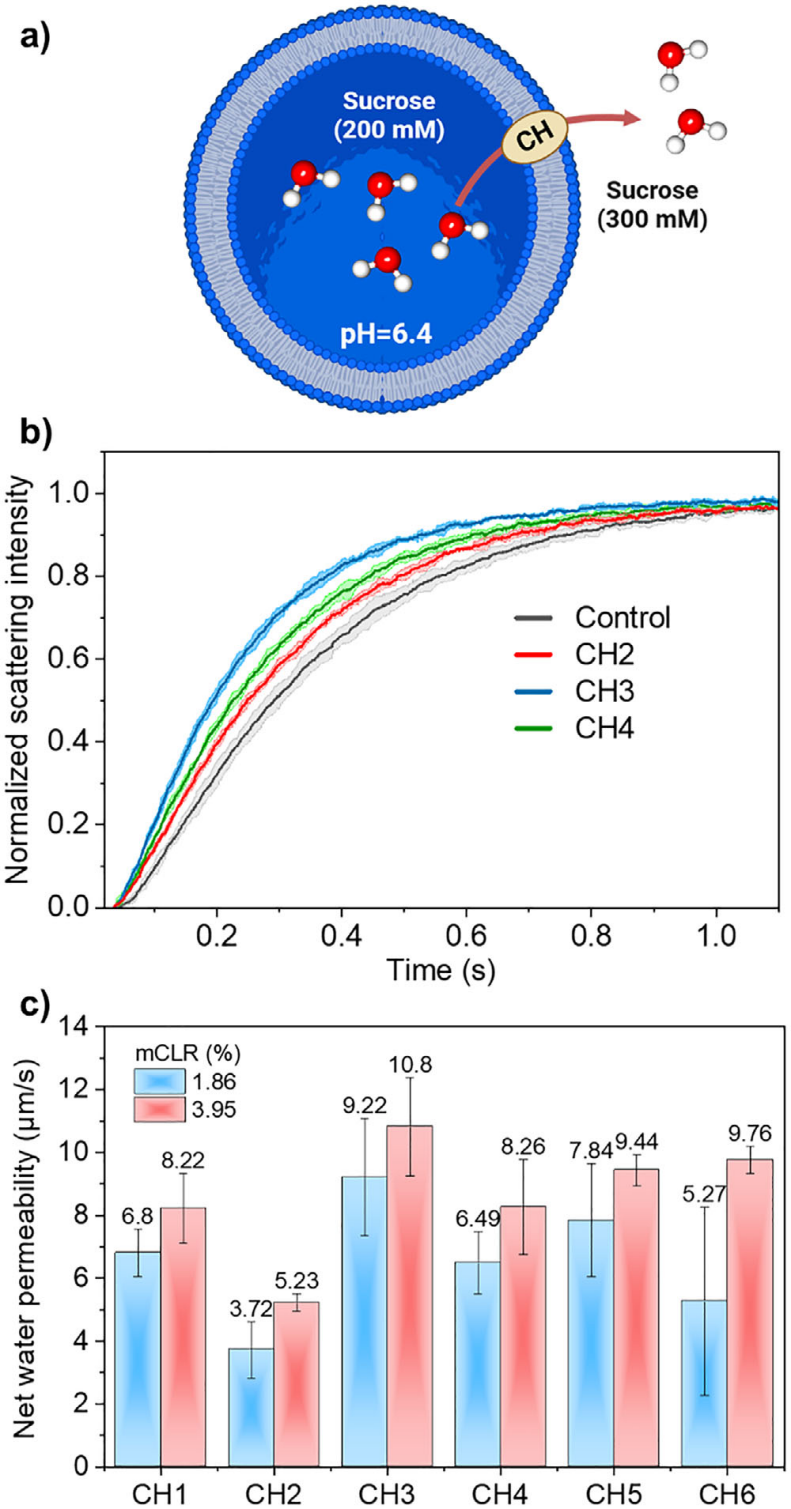
Quantification of water transmembrane transport facilitated by CH‐donor self‐assembled channels **CH1‐CH6**: (a) Schematic representation of the stop flow assay; (b) Stopped‐flow light scattering traces of LUVs containing **CH2**, **CH3**, and **CH4** at 3.95 mol%; (c) Net water permeabilities calculated from the light scattering traces for all CH‐donor transporters at 1.86 and 3.95 mol%.

Encouraged by the ability of the CH‐donor podands to form self‐assembled water channel as observed for **CH4**, we evaluated their water permeability using the stop‐flow technique. For this purpose, Large Unimolecular Vesicles‐LUVs (D = 100 nm) composed of a phosphatidylcholine/phosphatidyl‐serine/cholesterol (PC/PS/Chl) mixture in a 4:1:5 molar ratio were prepared, encapsulating a 200 mM sucrose solution buffered at pH 6.4. Compounds **CH1**–**CH6**, solubilized in dimethyl sulfoxide, were added to the LUVs at two different compound‐to‐lipid molar ratios (mCLR, mol%) and incubated for 30 min at 20° C. The vesicles were then exposed to a hypertonic sucrose solution (300 mM, pH 6.4), generating an osmotic pressure gradient Δ_osm_ = 100 mOsm/L (Figure 3a) that drives water efflux and vesicle shrinkage, which was monitored over time by light scattering. As shown in Figures 3b and S15–S20, an increase in the light scattering intensity relative to the control (no compound added) is observed for all transporters, indicating facilitated water permeation. Indeed, quantification of net water permeability (Figure 3c) revealed particularly high performance for **CH3**, higher than of imidazole‐based derivatives **CH5** and **CH6**. **CH4** and **CH1** exhibit intermediate performances, while **CH2** shows the lowest net water permeability among the series. Lowering the transporter concentration led to a decrease in net water permeability (Figure 3c), indicating concentration‐dependent channel self‐assembly behavior that may lie in the different self‐assembled water conducting pathways generated through polymorphic supramolecular scaffolds of **CH1**‐**CH6**. Specifically, ‐OBu substituents weaken the CH donor sites within **CH3**, leading to the formation of less stable HB water contacts within the water channel and thus reducing friction during water translocation. In contrast, the stronger CH binding sites in **CH2** are expected to slow down the water permeation by increasing friction. It is worth noting that, the water content within the channels, which is expected to be higher for **CH5** and **CH6** due to the presence of imidazole moieties [13, 14], also influences the overall water permeability. Therefore, a clear distinction between the individual factors governing water transport efficiency cannot be established. Nevertheless, the ability of weak C–H hydrogen‐bond donors to efficiently facilitate water transport (vide infra) is particularly important for the design of low‐friction AWCs and to modulate ion‐water selectivity.

### 2.3 Molecular Dynamics Simulations

**FIGURE 4:**
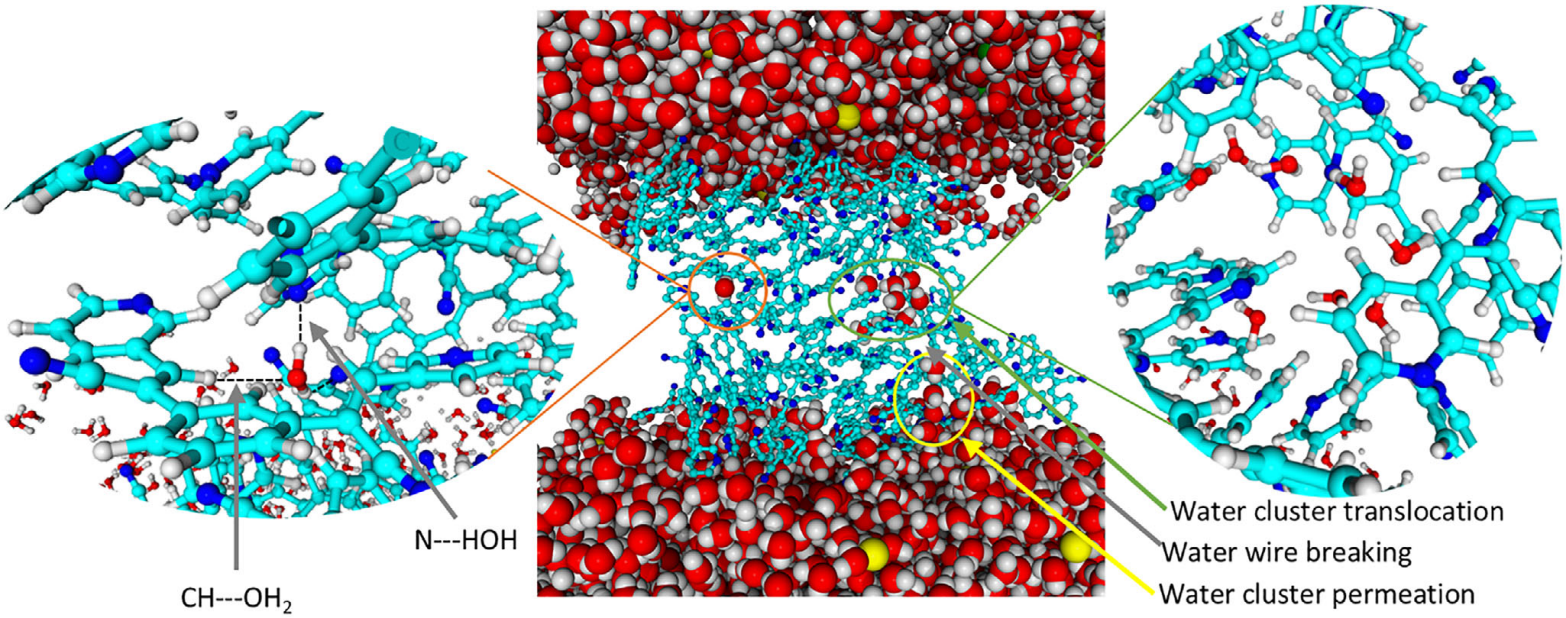
Molecular dynamics simulation snapshot at 25 ns showing water cluster permeation and translocation, along with single water molecule diffusion, through the stable self‐assembled aggregate of **CH4**. The phospholipid membrane is omitted for clarity.

To assess the stability of the channels within the lipid bilayer and to gain insights into water dynamics during translocation, we performed molecular dynamics (MD) simulations. A small patch from the resolved molecular structure of **CH4** was embedded into a POPC membrane. The self‐assembled aggregate remained stable over time (Figures 4 and S22). We observed that the initial molecular organization observed in the solid‐state structure of **CH4** is not fully preserved, as expected for a dynamic system, the HB and π–π interactions contribute to preserving a slightly disordered directional assembly, while hydrophobic interactions with the lipid bilayer ensured membrane insertion. Noteworthy, CN···HOH and N···HOH interactions are observed at the membrane/water interface, without evidence of extraction or migration of molecules into the aqueous phase. Although the solid‐state channels configuration observed in the x‐ray single crystal structure is not fully retained within the lipid bilayer, water permeation pathways enable water wire to enter the self‐assembled, dynamically rearranging aggregates stabilized by mutual H‐bonding with water. Importantly, water wire clustering is observed (Figure 4) and is stabilized within the **CH4** channels by multiple N···HOH, CN···HOH interactions, and, importantly, C–H···OH_2_ contacts. The presence of C–H···OH_2_ interactions is of crucial importance for facilitating water cluster translocation, C–H donors acting as HB donor water‐binding sites within the self‐assembled channel.

Notably, single water molecule diffusion through the aggregates is also possible, as observed for **CH4**. The water molecule is mainly located at the CH binding sites, with its lone pairs satisfied by the CH donors, while hydrogen‐bond acceptor interactions are provided by apical and basal N atoms (pyridine in **CH4**, imidazole in **CH5** and **CH6**, or the CN groups in all transporters). Water is practically hopping through the adaptive channel CH binding sites.

### 2.4 Ion Transport Studies

Ion transport across bilayer membranes was investigated using the 8‐hydroxypyrene‐1,3,6‐trisulfonic acid trisodium salt (HPTS) fluorescence assay [65, 66]. Large unilamellar vesicles (LUVs, 100 nm) composed of egg‐PC (EYPC) were prepared containing HPTS (10 µM) and different sodium salts (NaX, 100 mM) in PBS buffer at pH 6.4. Notably, varying the intravascular anion in the pH gradient assay is essential for accurately assessing both transport activity and anion selectivity [54]. The EYPC⊃HPTS (NaX)_in_ vesicles were suspended in appropriate external salt solutions (NaX)_out_, also buffered at pH 6.4, and placed in a quartz cuvette for further HPTS ratiometric fluorescence monitoring at 510 nm (excitation at both 405 and 460 nm). The transporter, added as a DMSO solution, was injected at *t* = ─40 s (i.e., 40 s before pH gradient creation) at concentrations of 0–4.31 mol% (relative to lipids), followed by NaOH injection at *t* = 0 s to create a transmembrane pH gradient (∼1.5 units) that drives ion transport. At *t* = 260 s, Triton X‐100 was added to lyse the vesicles and obtain the maximum HPTS fluorescence signal.

**FIGURE 5:**
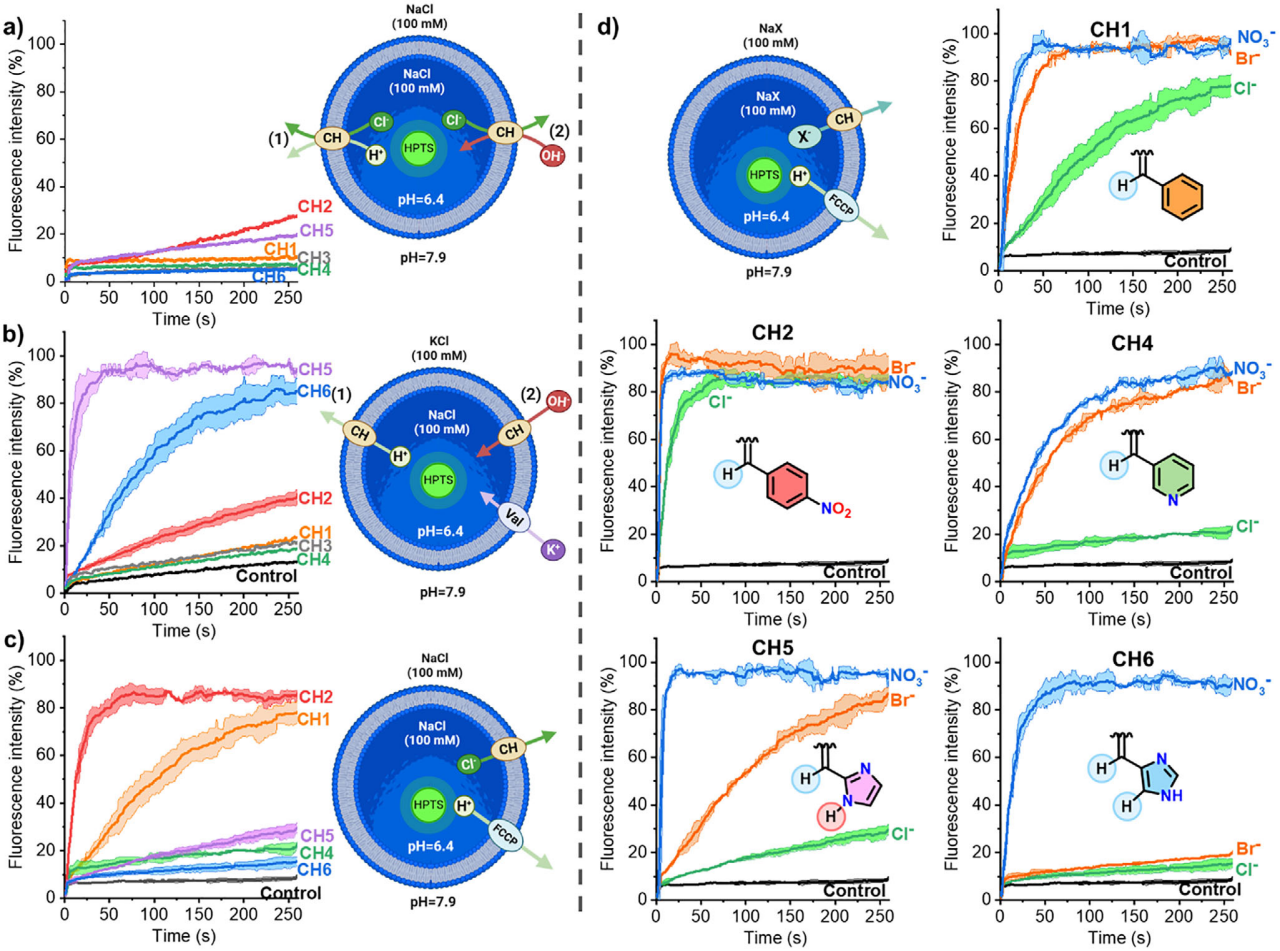
Time‐dependent HPTS fluorescence intensity change (%) corresponding to: (a) Electroneutral H^+^/Cl^−^ symport (1) or Cl^−^/OH^−^ antiport (2) facilitated by **CH1**–**CH6**; (b) H^+^ (1) or OH^−^ (2) uniport in the valinomycin‐coupled assay; (c) Cl^−^ uniport mediated by **CH1**–**CH6**; (d) Comparative Cl^−^, Br^−^, and NO_3_^−^ translocation performance of **CH1**–**CH6**. Schematic representations of each process are also provided. See the text and supporting information for the detailed procedure of each assay.

To study different transport mechanisms, protonophore‐coupled assays with FCCP and cationophore‐coupled assays with valinomycin were carried out (vide infra). We first examined the ability of **CH1**–**CH6** to mediate electroneutral transport using the EYPC ⊃HPTS (NaCl)_in_/NaCl_out_ assay configuration. As shown in Figure 5a, compounds **CH1**, **CH3**, **CH4**, and **CH6** do not support Cl^−^/H^+^ symport (or the equivalent Cl^−^/OH^−^ antiport), while **CH2** and **CH5** display low activity, with initial transport rates (k_0_) of 8.02·10^−2^ %s^−1^ and 3.90·10^−2^ %s^−1^, respectively. The observed behavior can be explained either by a H^+^ (or OH^−^) rate‐determining step that limits Cl^−^ transport, by intrinsically low activity toward Cl^−^ translocation, or by a combination of both factors. To elucidate the mechanism, we employed a cationophore‐coupled assay using valinomycin (0.108 mol%), known as a fast K^+^ carrier. Valinomycin ensure charge balance by transporting K^+^ in the event of H^+^ (or OH^−^) translocation facilitated by the CH donors. The coupled assay confirmed that **CH1**, **CH3**, and **CH4** exhibit very low H^+^ (or OH^−^) transport activity (Figure 5b), consistent with their weak CH donor strength, which is insufficient to bind OH^−^ strongly enough for transmembrane translocation. Furthermore, as shown previously, channel formation in these systems disrupts the water wire, effectively preventing proton transport via the Grotthuss mechanism. In contrast, **CH5** displayed high activity, with k_0_ reaching 8.0·10^3^ %s^−1^, while modest H^+^ (or OH^−^) activity was observed for **CH6** and **CH2**. For **CH2**, the enhanced CH donor strength likely facilitates OH^−^ binding and translocation, whereas in **CH5** and **CH6**, proton transport is more probable, owing to the presence of mobile protons on the imidazole moieties. To further quantify the H^+^ (or HO^−^) translocation efficacy in terms of EC_50_ (i.e., the concentration required to achieve half‐maximal transport), dose‐dependent transport experiments have been performed (Figures S24 and S28). As expected, **CH5** reached the lowest EC_50_ (0.775 mol%), followed by **CH2** (0.830 mol%) and **CH6** (2.58 mol%).

Having established that H^+^ (or OH^−^) translocation is most likely the rate‐limiting step, we employed a protonophore‐coupled assay using Carbonylcyanide‐p‐trifluoromethoxyphenyl‐hydrazone—FCCP (0.108 mol%) as a fast H^+^ carrier to effectively eliminate this limitation and directly assess the anion uniport capabilities of transporters **CH1**–**CH6**. Coupling FCCP with **CH2** resulted in the rapid dissipation of the pH gradient, reaching electrochemical equilibrium (plateau) within ∼60 seconds (Figure 5c). This indicates that **CH2** facilitates efficient Cl^−^ uniport (see also Table 1 for *k*_0_ values), consistent with its relatively high acidity as a CH donor. Interestingly, **CH1** achieved nearly the same performance as **CH2** (i.e., ∼80%) after 260 s of effective transport at 4.31 mol%; however, with a considerably lower transport rate and a much higher EC_50_ (Table 1 and Figure 5c). In contrast, the other compounds were very weakly active. This result is somewhat surprising for transporter **CH5**, which contains strong imidazole NH HB donors; however, its low Cl^−^ transport activity is explained by theoretical calculations (vide infra).

**TABLE 1:** Transmembrane transport parameters (*k*_0_, EC_50_, and Hill coefficient) for the uniport of Cl^−^, Br^−^, and NO_3_^−^ facilitated by **CH1**–**CH6**, along with their binding properties: Experimental (*K*_a_) and theoretical (non‐bonding interaction energies; see text for details).^a^

Transporter	Anion	k_0_ ^b^ (%s^−1^)	EC_50_ ^c^ (mol%)	Hill number	Ka^d^ (M^−1^)	*E*(Ha···X^−^)^e^ (kJ mol^−1^)	*E*(Hb···X^−^)^e^ (kJ mol^−1^)	*E*(Hc···X^−^)^e^ (kJ mol^−1^)
**CH1**	Cl^−^	1.03	1.84	2.1	2.47	−9.14	−7.02	−4.52
	Br^−^	92.13	1.99	3.7	2.12	−7.03	−5.93	−2.90
	NO_3_^−^	1.72·10^4^	1.45	3.3	‐^f^	−10.50	−9.56	−5.98
**CH2**	Cl^−^	4.00·10^2^	0.0875	1.4	5.11	−10.15	−8.03	−6.57
	Br^−^	1.61·10^13^	0.0144	1.9	3.08	−8.61	−6.15	−3.86
	NO_3_^−^	7.35·10^12^	0.0107	2.4	1.64	−11.54	−9.95	−6.68
**CH4**	Cl^−^	9.34·10^−2^	‐^f^	‐^f^	3.52	−10.87	−6.43	−6.00
	Br^−^	3.65	0.778	2.9	2.95	−8.59	−5.35	−3.89
	NO_3_^−^	4.66	0.228	1.1	‐^f^	−11.63	−6.76	−6.68
**CH5**	Cl^−^	1.45·10^−2^	2.48	4.3	8.49	−6.19	−17.64	0.00
	Br^−^	1.10	1.33	2.0	2.00	−5.33	−12.11	0.00
	NO_3_^−^	3.27·10^8^	0.0107	3.0	<1	−10.64	−36.39	−5.49
**CH6**	Cl^−^	7.85·10^−2^	‐^f^	‐^f^	3.33	−7.05	−6.95	−3.19
	Br^−^	6.45·10^−2^	‐^f^	‐^f^	2.59	−5.53	−5.97	−2.44
	NO_3_^−^	3.11·10^2^	0.859	1.3	‐^f^	−7.52	−15.73	−3.51
**NH1**	Cl^−^	5.88·10^8^	0.229	1.7	16^g^	−15.70	−6.84	−9.10
	Br^−^	2.72·10^2^	0.0224	0.8	‐^h^	−11.23	−5.95	−6.81
	NO_3_^−^	2.17·10^6^	0.0674	1.1	‐^h^	−22.20	−8.69	−12.14

^a^ Compound **CH3** is not included in the table as it does not facilitate the translocation of any of the studied anions, however its theoretical binding properties are included in Tables S2 and S3.

^b^ Determined at 4.31 mol%.

^c^ Determined at 250 s.

^d^ Determined in DMSO‐*d6* by NMR titration, see ESI for errors in affinity constants.

^e^ Computed in bilayer hydrophobic medium (*ε* = 2) at B3LYP‐D3/def2‐TZVP level of theory.

^f^ Not determined due to low activity.

^g^ from ref [62].

^h^ Not determined.

**FIGURE 6:**
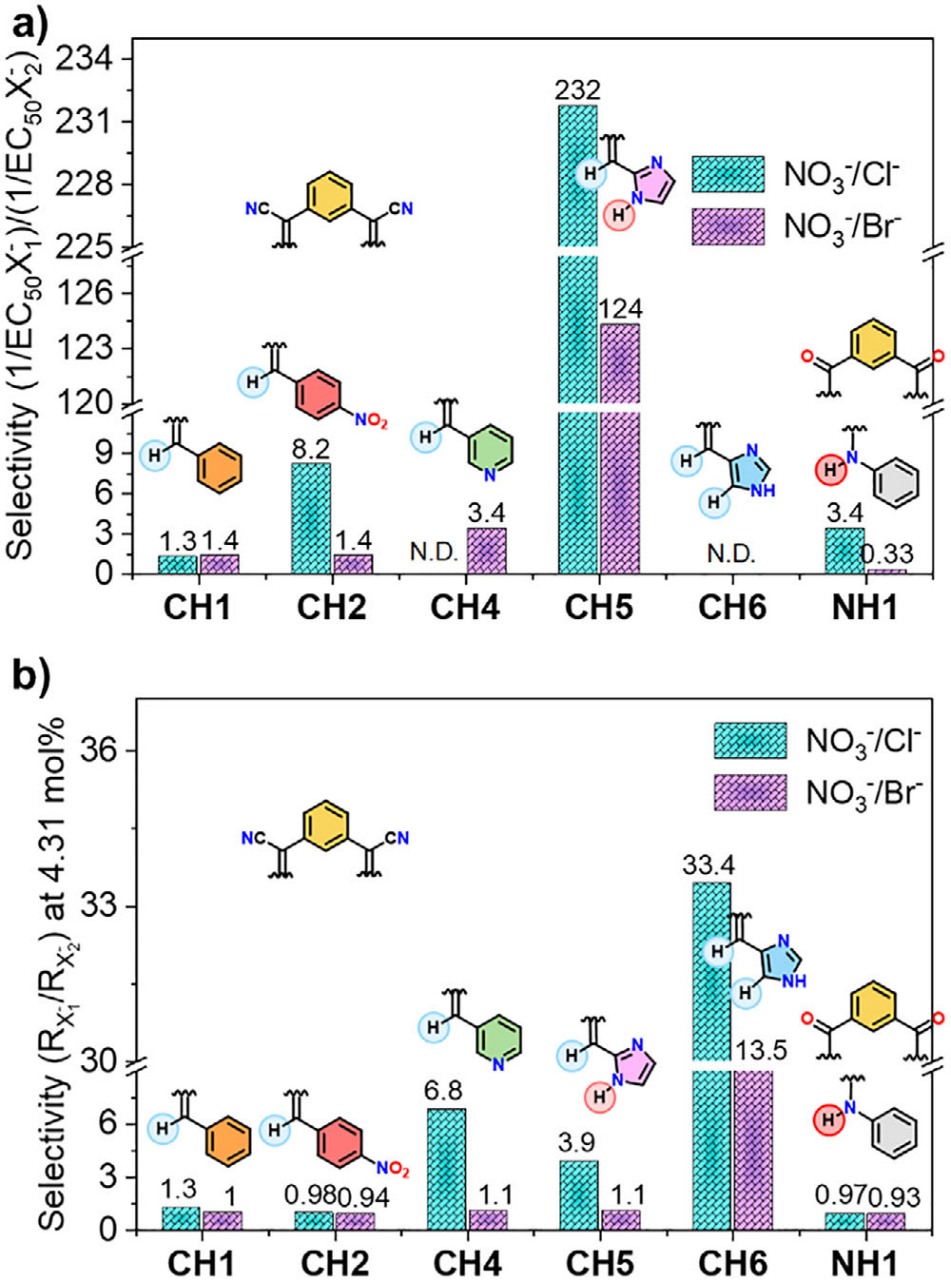
Anion transport selectivity expressed as (a) the ratio of the inverse EC_50_ values and (b) the ratio of fractional anion transport activity at 4.31 mol% (see text for details). For cases with too low Cl^−^ transport activity, reliable EC_50_(Cl^−^) values, and consequently selectivity ratios could not be determined.

Next, we examined the uniport of anions other than chloride, specifically Br^−^ and NO_3_^−^. For this purpose, we employed the FCCP‐coupled assay, replacing both intra‐ and extravesicular anions accordingly (see Supporting Information for more details). To quantify the transport selectivity, we have calculated the reverse EC_50_ ratios (i.e., $\left(1/EC50_{X_{1}^{-}}\right)$/$\left(1/EC50_{X_{2}^{-}}\right)$) where possible (vide infra), and fractional anion transport activity ratios ($R_{X_{1}^{-}}$/$R_{X_{2}^{-}}$). As can be observed in Figure 5d, transporters **CH4** and **CH5** show similar behaviors, with a clear preference for nitrate and bromide transport, that is, transport selectivity follows the order NO_3_^− ^> Br^−^ >> Cl^−^. The same trend is observed also for **CH1** (Figure 5d); however, with an increase in Cl^−^ uniport performance, while **CH3** show inactivity toward all tested anions in line with its very weak CH donors. **CH2**, on the other hand, reached maximum transport at 4.31 mol% for all tested anions, indicating a lower selectivity (as further confirmed by dose‐dependent experiments). Interestingly, transporter **CH6** exhibits 33‐fold higher selectivity for NO_3_^−^ over Cl^−^  and 14‐fold higher selectivity over bromide, attributable to its harmonized weak CH donors and optimally sized binding cavity for nitrate, as further sustained by DFT calculation (see below). Dose‐dependent transport experiments were also performed for Br^−^ and NO_3_^−^ to obtain EC_50_ values together with the Hill numbers related to the degree of cooperativity required for translocation (Table 1). Overall, **CH2** and **CH5** exhibited the lowest EC_50_(NO_3_^−^) values (0.0107 mol%) and a relatively high degree of cooperativity in agreement with a channel mechanism (vide infra). Notably, **CH5** displayed exceptional anion selectivity, showing over 200‐fold preference for NO_3_^−^ over Cl^−^ and >100‐fold preference for NO_3_^− ^over Br^−^ as determined using EC_50_ values (Figure 6a). This high nitrate selectivity arises from the fine‐tuning of HB donor strength and the geometry of the binding cavity, as detailed in the DFT calculations section. Unfortunately, a direct comparison with **CH6** could not be made, as it displayed almost no activity toward Br^−^ or Cl^−^ translocation, preventing the determination of reliable EC_50_ values. Nevertheless, it is noteworthy that **CH6** exhibits EC_50_(NO_3_^−^) < 1 mol% while remaining very low active toward Cl^−^ transport even at higher concentrations (e.g., 4.31 mol%, Figure 6b). Importantly, the resulting nitrate‐over‐chloride selectivity, is maintained across the explored concentration range (Figure S33). This contrasts with **CH5**, for which maximum selectivity is observed at lower concentration and decreases gradually at higher loadings (Figures 6b and S33), dropping to approximately fourfold at high concentrations (4.31 mol%, for instance). This is consistent with the higher degree of cooperativity (as suggested by the Hill number) required to compensate for the unbalanced Cl^−^ binding within the cavity of **CH5**. Meanwhile, **CH2** shows a modest yet discernible NO_3_^−^/Cl^−^ selectivity (Figure 6a); however, this selectivity rapidly diminishes as the transporter concentration increases (Figures 6b and S33). In contrast, **CH1** displays lower and comparable NO_3_^−^/Cl^−^ and NO_3_^−^/Br^−^ selectivity due to decreased NO_3_^−^ uniport efficacy, while **CH4** shows superior selectivity. Despite the structural similarities of **CH1** and **CH4**, the distinct translocation behaviors can be rationalized by differences in anion binding, as discussed below.

We also compared the transport selectivity of the CH donors with that of a structurally related NH donor podand, **NH1** (Scheme S2), which is analogous to **CH1** [62]. **NH1** was found to transport Cl^−^ more rapidly (*k*_0_ = 5.88·10^8^%s^−1^) than any of the CH donor podands **CH1‐CH6**, consistent with its stronger and balanced HB donor sites. Due to the high Cl^−^ transport activity, **NH1** exhibits only modest NO_3_^−^ over Cl^−^ selectivity (≈3), which rapidly decreases with increasing transporter concentration, mirroring the behavior observed for **CH2** (Figures 6b and S33). By comparison, although **CH1** reaches a slightly lower selectivity (Figure 6a), its dose‐dependent selectivity is considerably more robust and decreases less steeply than that of the stronger HB donors **NH1** and **CH2** (Figures 6b and S33). Furthermore, deliberate disruption of harmonized convergent chloride binding in **CH4**, as revealed by DFT calculations (see below), effectively suppresses Cl^−^ translocation and leads to enhanced NO_3_^−^/Cl^−^ selectivity that is retained even at higher transporter loadings (Figure 6b).

In the pH gradient assay, anion uniport is driven by the pH gradient, creating an anion gradient across the membrane; therefore operating under a secondary active transport regime. To probe passive anion transport driven solely by an anion concentration gradient, we performed experiments in the absence of any pH gradient. Specifically, an EYPC⊃HPTS (NaX)_in_/ (NaGluconate)_out_ assay was implemented, in which an outwardly directed anion gradient (X‐ = Cl‐, Br‐, NO3‐) is established across the membrane. Upon transporter‐mediated anion efflux, charge compensation occurs through proton efflux via the FCCP pathway. As a result, the anion gradient is progressively dissipated while a pH gradient is created until electrochemical equilibrium is reached. Notably, sodium gluconate (NaGluc) is a bulky, highly hydrated anion that is not transported by conventional biomimetic anion transporters.

**FIGURE 7:**
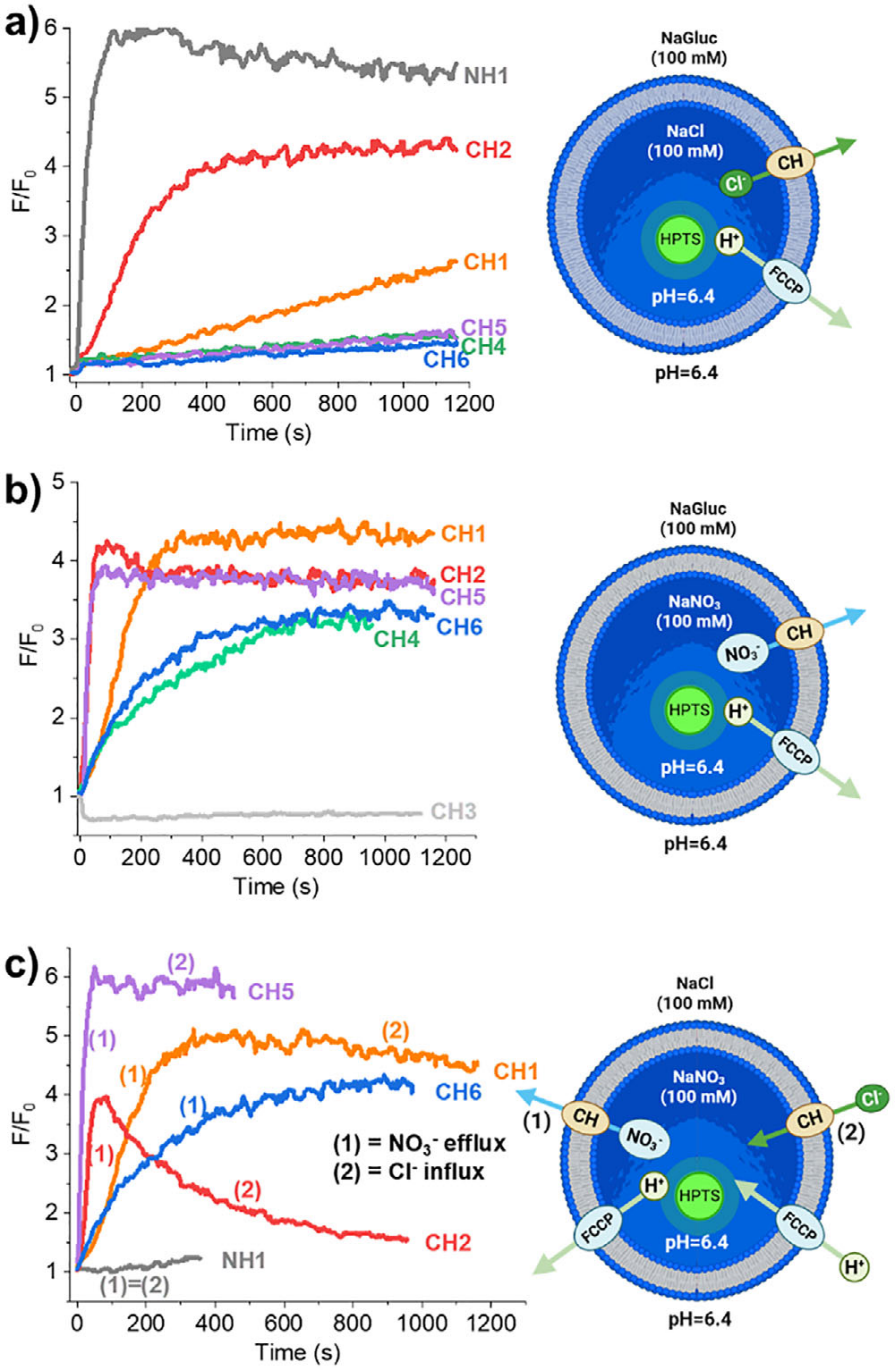
HPTS fluorescence intensity change (F/F_0_) versus time and schematic representations corresponding to: (a) Cl^−^ passive uniport by **CH** derivatives and **NH1** at 4.31 mol%, accompanied by H^+^ efflux through FCCP; (b) NO_3_^−^ passive uniport by C─H donors at 4.31 mol%, accompanied by H^+^ efflux through FCCP; (c) competitive transport, showing NO_3_^−^ efflux accompanied by H^+^ efflux through FCCP (process 1) versus Cl^−^ influx accompanied by H^+^ influx through FCCP (process 2). For **NH1**, chloride influx equals nitrate efflux, thus no net change in the internal pH.

As shown in Figure 7a, rapid Cl^−^ efflux is mediated by the **NH1** transporter, whereas **CH2** exhibits moderate activity and **CH1** shows weak, but clearly discernible transport. In contrast, **CH4**, **CH5**, and **CH6** display only marginal Cl^−^ transport. This activity trend closely mirrors the performance order observed in the pH gradient assay. Replacing the intravascular anion with nitrate leads to rapid passive NO_3_^− ^efflux except in the case of **CH3** (Figure 7b), thereby demonstrating the ability of the C–H hydrogen‐bond donors, particularly **CH4**, **CH5**, and **CH6** to mediate highly selective passive transmembrane nitrate transport. A detailed comparison of passive NO_3_^−^, Br^−^, and Cl^−^ translocation efficiencies for each transporter is presented in Figure S34.

Finally, anion transport selectivity was further evaluated using the competitive assay in which NaCl was present in the extravesicular medium and NaNO_3_ in the intravascular space [54]. As shown in Figure 7c, the strong H‐bond donor **NH1** displays no detectable selectivity: NO_3_^− ^efflux and Cl^−^ influx occur at comparable rates, resulting in no net proton flux across the membrane. In contrast, the corresponding C–H HB donor **CH1** exhibits rapid NO_3_^−^ efflux (accompanied by proportional H^+^ efflux), followed by slow Cl^−^ influx (accompanied by proportional H^+^ influx). For **CH2**, which features stronger C–H HB donor sites, reduced selectivity is observed, as evidenced by the rapid Cl^−^ influx subsequent to the NO_3_^− ^initial efflux.

By contrast, the high NO_3_^−^/Cl^−^ selectivity of **CH5** and **CH6**, as discussed above, is reflected in a very slow Cl^−^ influx subsequent to NO_3_^−^ fast efflux. We further extended the competitive assay to evaluate NO_3_^−^/SO_4_2^−^ and NO_3_^−^/HCO_3_^−^ transport selectivities. Accordingly, no SO_4_2^−^ influx was observed for any of the studied transporters (Figure S35). On the other hand, HCO_3_^−^ influx (following the rapid initial NO_3_^−^ efflux) was observed only for the strongest C–H donor transporter **CH2**, whereas the other CH derivatives showed no detectable HCO_3_^−^ transport (Figure S35). Thus, the anion transport selectivity generally follows the order NO_3_^−^> Br^−^≫ Cl^−^ > HCO_3_^− ^> SO_4_2^−^, consistent with the Hofmeister series [67]. Notably, the selectivity shifts strongly toward nitrate when the binding mode and strength (e.g. in **CH4**, **CH5** and **CH6**, see also DFT calculations) fail to effectively stabilize more kosmotropic anions (including Cl^−^).

One must mention that carboxyfluorescein (CF) leakage assays were performed and revealed no detectable membrane leakage for any of the studied transporters at a compound‐to‐lipid molar ratio of 4.31 mol% (Figure S36).

Taken all together, these results demonstrate that weak HB donors, combined with precise modulation of binding pocket geometry, provides a straightforward and effective strategy for achieving highly nitrate‐selective transmembrane transport.

### 2.5 Ion Transport Mechanism

**FIGURE 8:**
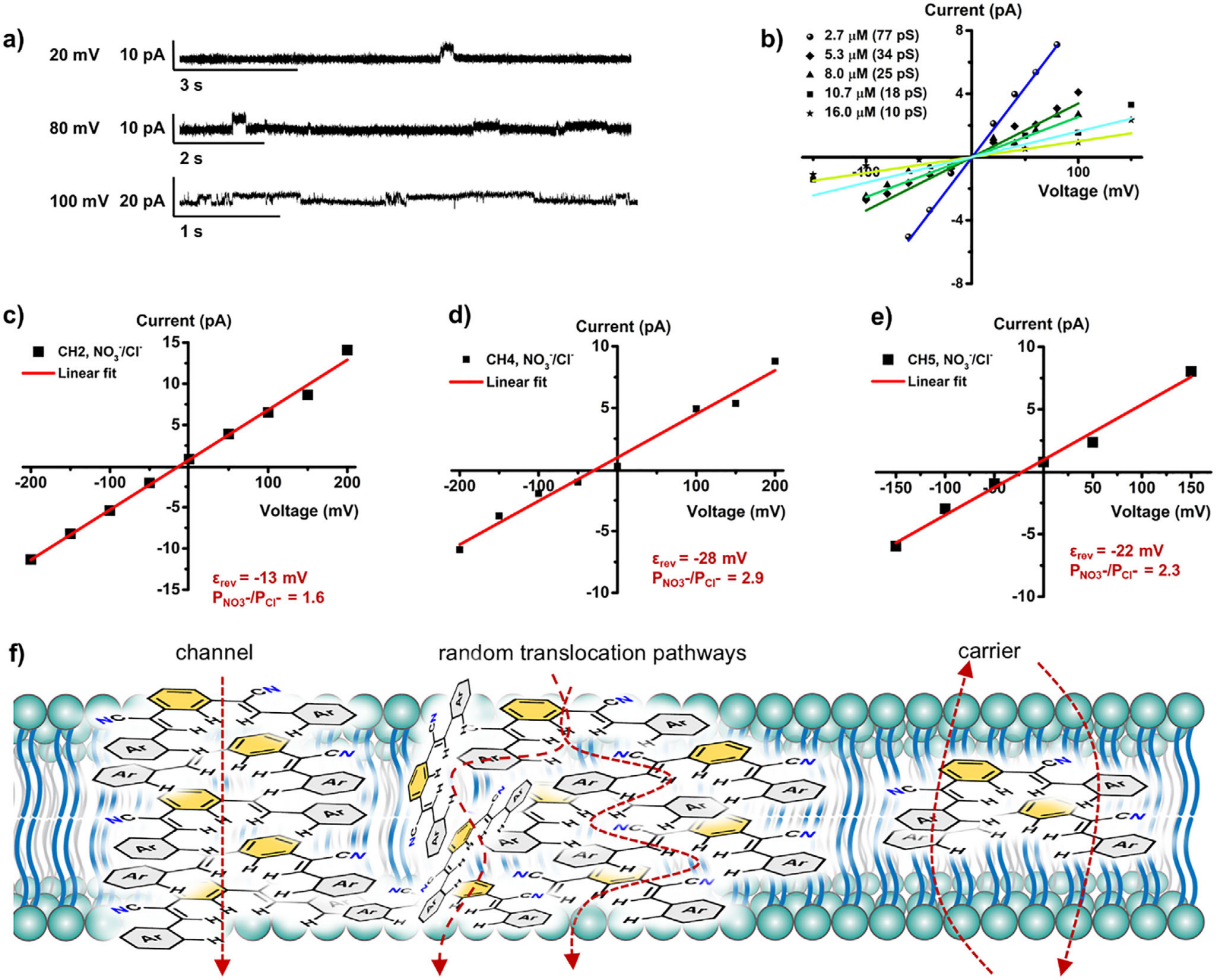
(a) Single‐channel currents of **CH5** (5.3 µM) recorded in symmetrical NaNO_3_ (1 M) solutions at +20, +80, and +100 mV. (b) *I–V* plots of **CH5** at different concentrations in symmetrical NaNO_3_ (1 M) solutions. *I–V* plots of **CH2** (c), **CH4** (d), and **CH5** (e) recorded 5.3 µM in unsymmetrical (NaNO_3_)_trans_/(NaCl)_cis_ solutions (1 M salt concentration); the calculated $P_{NO_{3}^{-}}/P_{Cl^{-}}$ selectivities are also given. (f) Schematic representation of the proposed transport mechanisms.

Planar bilayer patch‐clamp experiments were performed to elucidate the transport mechanism. In a symmetrical NaNO_3_ cell configuration, **CH5** forms conductive channels showing an interesting concentration‐dependent conductance (Figure 8a, b). Specifically, increasing the compound concentration leads to a decrease conductance from 77 pS at 2.7 µM to 10 pS at 16 µM (Figure S38). This behavior is likely associated with an aggregation‐induced disruption of the channel conducting pathways. In contrast, no channel openings were observed for **CH6** and **CH1** (Figure S42). The NO_3_^−^/Cl^−^ selectivity ($P_{NO_{3}^{-}}/P_{Cl^{-}}$) was further determined using an asymmetric nitrate/ chloride configuration (1M NaNO_3_ in the *trans* compartment and 1M NaCl in the *cis* chamber). **CH5** and **CH4** display the selectivities of 2.3 and 2.9, respectively, whereas **CH2** shows a lower value of 1.6 (Figures 8c,d, and S39–S41). These results are in good agreement with the selectivity trends determined from the HPTS assay (0.1 M salt) (Figure 6) with lower selectivities in terms of absolute values obtained in the planar bilayer experiments (1 M salt) compared with those from the HPTS assay, likely due to the different driving forces governing transport.

Taken together, we envision the following transport mechanism. At resting (i.e., in the absence of a transport‐driving stimulus), and lacking strongly directional self‐assembling motifs such as ureas, all CH derivatives are expected to self‐organize within the lipid bilayer in different supramolecular architectures, depending on their structure, conformers interconversion, temperature, concertation etc. Nevertheless, useful structural insight can be drawn from the solid‐state structure of **CH4** and the stabilization of its aggregates within the membrane (vide supra). Upon a driving force (i.e., anion gradient, pH gradient or applied voltage), anion permeation templates molecular reorganization to open channel conducting pathways as observed for **CH2**, **CH4,** and **CH5**. Increasing compound concentration likely leads to supramolecular polymorphic transitions from directional nitrate channel flow to multiple random non directional nitrate diffusion pathways (Figure 8f). **CH1** and **CH6**, on the other hand, seem to operate through random nitrate diffusion pathways (as no channel openings were observed), a classical carrier mechanism, or a combination of both. Notably, the contribution of the carrier‐type mechanism likely becomes more significant at lower loading concentrations, as also previously proposed for alkyl‐ureido crown ether self‐assembled channels [68], or oligo(aryl‐triazole) NO_3_^−^/Cl^−^ exchangers [60]. Such multiple conducting species are an example of the supramolecular polymorphism [69].

To gain more insight into the proposed mechanisms, we also performed variable‐temperature in Dipalmitoylphosphatidyl‐choline (DPPC) and cholesterol‐dependent transmembrane transport experiments. DPPC lipids undergo a gel‐to‐liquid phase transition at ∼41° C. In a channel‐mediated transport scenario, ion translocation is expected to occur even below the transition temperature (e.g., at 35°C), whereas carrier‐mediated transport generally requires the liquid phase of the membrane [70].

Thus, implementing the DPPC⊃HPTS (NaNO_3_)_in_/(NaNO_3_)_out_ pH gradient assay, we observed that at 35° C translocation occurs for all **CH1‐CH5** derivatives excepting **CH6**, whose activity is only detected above the phase transition, at 45° C (Figure S43). Next, the EYPC⊃HPTS (NaNO_3_)_in_/(NaGluconate)_out_ anion‐gradient assay was performed at different cholesterol contents (0, 15, 30, and 45 mol% relative to EYPC lipids) to modulate membrane rigidity. In the case of a carrier mechanism, a substantial decrease in transport efficiency is generally expected as membrane rigidity increases (i.e., with increasing cholesterol concentration) [71, 72]. Indeed, **CH6** exhibits a pronounced decrease in activity even at higher loading concentrations as the cholesterol fraction increases (Figure S44). In contrast, **CH5** and the other transporters show only a slight reduction in transport rates. These observations are consistent with the DPPC experiments and further support a classical carrier‐type transport mechanism for **CH6**. Notably, **CH1** exhibits channel‐like behavior in the DPPC and cholesterol‐dependent experiments, while no channel opening is observed in planar bilayer measurements. This observation is consistent with the above‐proposed mechanism of random nitrate diffusion rather than continuous flow through well‐defined channels. To better understand the transport through **CH1**‐**CH6**, variable‐temperature (VT) EYPC⊃HPTS nitrate gradient experiments were performed. **CH4**, **CH5,** and **CH6** display pseudo‐first‐order kinetic behavior across the temperature range investigated (10–45° C). In contrast, **CH1** and **CH2** exhibit sigmoidal cooperative transport profiles below 20° C (Figure S45), with an initial time‐lag phase that may arise from adaptive membrane insertion, conformational changes and/or polymorphic transitions required to open nitrate translocation pathways. For **CH4**, **CH5,** and **CH6**, an initial lag phase does not appear to be rate‐limiting, and transport begins immediately following the expected pseudo‐first‐order kinetics. Both translocation through more well‐defined channel pathways in **CH4** and **CH5**, as well as a carrier mechanism in the case of **CH6**, are compatible with this kinetic behavior. Fitting the experimental transport traces to obtain the observed rate constants (*k*_obs_) followed by Arrhenius and Eyring analyses, the activation parameters for **CH4**, **CH5,** and **CH6** were determined (Table S1 and Figure S46–S48). **CH5** shows the lowest activation energy (i.e., *E_a_ =* 24.7 kJ mol^−1^), followed by **CH6** (52.8 kJ mol^−1^) and **CH4** (63.6 kJ mol^−1^), in close agreement with the above‐discussed transport efficiencies (vide supra). The most negative activation entropy of **CH5** (Δ*S* ^#^ = ‐192.6 J mol^−1^ K^−1^) in comparison with **CH4** (Δ*S* ^#^ = ‐84.5 J mol^−1^ K^−1^) again underlines the higher (almost three times) degree of cooperativity reached by **CH5**, in close agreement with the Hill coefficients (Table 1). A similar trend is also observed for **CH5** versus **CH6** and **CH4** versus **CH6** (see Table S1).

### 2.6 Experimental and Theoretical Ion Binding Studies

Anion binding at the membrane interface is a necessary, but not sufficient condition for transmembrane transport. During translocation, the anion must be effectively shielded from the hydrophobic bilayer environment via a 1:1 or a cooperative binding mode. Consequently, binding affinities measured in solution, typically expressed as association constants (*K*_a_), do not necessarily correlate with transport efficiency or selectivity. Nevertheless, to help rationalize the observed trends in transport performance, we carried out ion‐binding studies in DMSO using ^1^H‐NMR titration experiments. Upon titration of podands **CH1**–**CH6** with Cl^−^ and Br^−^ (added as TBA^+^X^−^ salts), deshielding of the CH‐proton singlet was observed (Figures S49–S68) for all compounds except **CH3**, whose binding sites are too weak to bind anions, consistent with its lack of transport activity. In contrast, nitrate binding was detected only for the strongest CH donor (**CH2**) and for the synergistic CH/NH donor (**CH5**). Association constants for the 1:1 binding mode were obtained by fitting the CH‐proton chemical shift changes as a function of anion equivalents using supramolecular.org [73], and are summarized in Table 1. As expected, and in agreement with DFT predictions (Figure 1), replacing NH with CH hydrogen‐bond donor markedly decreases chloride binding affinity and further Cl^−  ^translocation efficacy, as illustrated by the comparison between **NH1** and **CH1** (vide supra). Further modulation of CH donor strength is directly reflected in the binding affinities: **CH1** exhibits the lowest Cl^−^ and Br^−^ affinities (as also very recently reported by Valkenier and coworkers) [57], **CH4** and **CH6** display comparable binding constants, while **CH2** and **CH5** show slightly higher affinities. Notably, only **CH2** supports fast Cl^−^ uniport, highlighting the importance of binding mode as discussed below.

**FIGURE 9:**
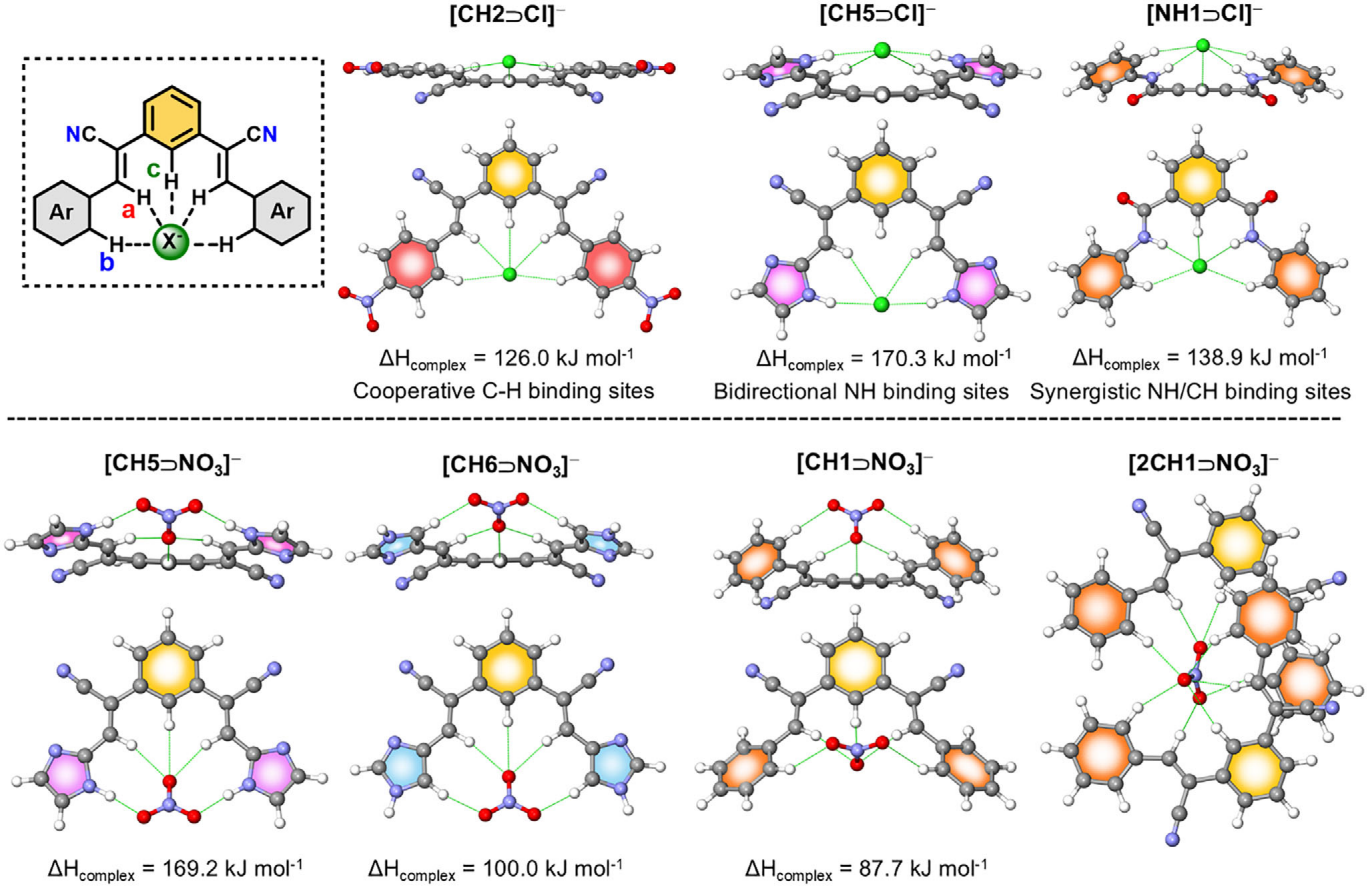
Schematic representation of anion (X^−^) interactions with the binding sites of the studied transporters, along with the relevant complex geometries optimized at the B3LYP‐D3/def2‐TZVP level of theory in a hydrophobic medium with dielectric constant set to two. The calculated binding enthalpies are given in kJ mol^−1^, and the corresponding non‐bonding interaction energies for each anion–transporter complex are summarized in Table 1.

To gain an atomistic understanding of the anion binding within the hydrophobic membrane bilayer, and thereby obtain further insights into their transport behaviors, density functional theory (DFT) calculations were performed at the B3LYP‐D3/def2‐TZVP level of theory (see ESI for computational details). The DFT calculations were focused on the 1:1 **CH**–anion complexes, even though the translocation higher degrees of supramolecular cooperativity can be considered. This approach provide valuable insights into the anion binding mode and strength, relevant to the overall transport mechanism. We firstly sought to explain the markedly different Cl^−^ transport behavior of **CH2** and **CH5**, as the latter does not translocate despite exhibiting higher chloride affinity based on *K*_a_ values in Table 1 and the theoretical binding enthalpies in Figure 9. In addition, the contrasting behaviors observed for **CH1** versus **CH4**, **CH5** versus **NH1**, and other related trends were also discussed. As the overall binding affinities do not fully explain the observed transport permselectivities, the anion binding interactions were decomposed into individual H···X^−^ non‐bonding contributions (Figure 9) using the Quantum theory of atoms in molecules (QTAIM) formalism [74, 75]. The energies of the H···X^−^ interactions were estimated from the potential energy density at the bond critical points (BCPs) using the Espinosa–Molins–Lecomte relationship and are listed in Table 1 [76]. As can be observed in Figure 9, the larger binding cavity of **CH5**, together with its bidirectional otherwise strong NH···Cl^−^ interactions (Table 1), results in chloride binding being dominated by the NH donors, thereby significantly weakening the CH···Cl^−^ contributions (Table 1 for the interaction energies and Table S3 for the non‐bonding interaction distances and angles). This reduces the effectiveness of convergent cooperative chloride stabilization and likely does not promote chloride‐templated formation of efficient translocation pathways within the self‐assembled aggregates. Furthermore, conformational changes partially inactivate the binding site. Thus, the observed slow Cl^−^ transport (Table 1) requires a high degree of molecular organization (reflected by a high Hill number) to mitigate chloride dehydration cost. It is also noteworthy that in the nearly coplanar geometry of [**CH5⊃Cl]^−^** (Figure 9), chloride remains largely exposed to the hydrophobic lipid bilayer, hampering also translocation through a hypothetical classical carrier mechanism. Similarly, in‐plane and unbalanced binding combined with weak C–H···Cl^−^ contacts completely suppresses chloride and bromide transport in **CH6**. In contrast, in **CH2** and even in **CH1**, a well‐balanced cooperative convergent action of CH donors stabilizes Cl^−^, facilitating the removal of its hydration shell upon permeation. Importantly multiple contacts facilitate anion‐templated dynamic formation of chloride translocation pathways in a channel mechanism (**CH2**) or a random diffusion scenario (**CH1**). On the other hand, the loss of CH_b_···Cl^−^ contacts in **CH4** (Table 1) explains the suppressed Cl^−^ transport activity in this derivative. Meanwhile, the stronger NH HB donors in **NH1** led to a significantly more effective Cl^−^ translocation than **CH2** and **CH1**. Importantly, unlike in **CH5**, the strong action of N–H donors in **NH1** does not disrupt the C–H contributions, highlighting once again the importance of balanced convergent anion binding contributions rather than overall binding strength alone. Furthermore, the transient binding mode also contributes to the degree of supramolecular cooperativity required for translocation. Specifically, the out‐of‐plane geometries observed for **[NH1⊃Cl]** ^−^ and **[CH1⊃Cl]^−^** promote the formation of cooperative 2:1 and/or 3:1 complexes (Figure S75), in agreement with the Hill coefficients (Table 1). In contrast, **CH2** adopts a nearly planar binding geometry that enables chloride translocation even in a 1:1 binding stoichiometry, as reflected by its Hill coefficient below 1.5. As far as nitrate transport is concerned, **CH5** and **CH6** exhibit the highest nitrate selectivities, as their binding cavities are optimal to accommodate nitrate ([**CH5⊃NO_3_]** ^−^ and [**CH6⊃NO_3_]** ^−^ in Figure 9, the calculated H···ONO_2_^−^ interaction energies in Table 1, and bond length and angles in Table S3). Yet, **CH5** shows superior activity in terms of both transport rates and EC_50_, due to its enhanced ability to recognize the anion at the membrane interface and to adaptively self‐reorganize into channel assemblies capable of sustaining nitrate conductance.

Unlike Cl^−^, despite the similar binding affinities in the hydrophobic membrane (Figure 9), nitrate forms multiple and balanced interactions with HB donors of **CH5,** facilitating the anion‐templated formation of channel pathways as well as efficient anion hopping during translocation. **CH4**, **CH1,** and even **CH2** also display high‐to‐moderate nitrate selectivity, although constraining the binding cavity induces out‐of‐plane nitrate binding (see [**CH1⊃NO_3_]^−^** in Figure 9). Nevertheless, this is not detrimental for translocation performance, as nitrate can be stabilized within cooperative assemblies (such as [**2CH1⊃NO_3_]^−^** in Figure 9), in agreement with the above discussed cooperative proposed mechanisms.

### 2.7 Water Versus Anion Transport

Weakening the HB donor strength results in strongly suppressed chloride transport and a shift toward selective nitrate translocation. When the C–H···anion interactions become too weak, as in **CH3**, even nitrate transport is suppressed. In contrast, water transport is not suppressed and may even be slightly enhanced as the C–H···OH_2_ interactions weaken. These differences arise from the fundamentally distinct nature of the two processes. Water permeation is associated with HB strength of channels surfaces that act primarily as “lubricating” interaction sites during water translocation rather than as strong stabilizing binding sites. By contrast, anion translocation requires full or at least partial dehydration of the anion upon translocation, as well as effective shielding from the hydrophobic membrane. Therefore, the HB donors act as stabilizing sites during translocation. It is worth noting that transport of a fully hydrated anion through the **CH1**‐**CH6** derivatives is unlikely, considering the narrow permeation pathways involved (see also Figure 4). Consequently, small variations in hydrogen‐bond donor strength have only a minor effect on CH···water interactions, as reflected by the small difference of ∼6 kJ mol^−1^ in calculated water binding enthalpies between the strongest C–H donor (**CH2**) and the weakest (**CH3**), while only 1 kJ mol^−1^ difference between **CH1** and **CH3** (see also Figure S77). In contrast, nitrate stabilization differs by approximately 60 kJ mol^−1^ between **CH2** and **CH3** and by nearly 12 kJ mol^−1^ between **CH1** and **CH3**, explaining the much greater sensitivity of anion transport to the hydrogen‐bond donor strength of the transporter. Therefore, by finely tuning the strength of HB sites, transport selectivity could be modulated from efficient Cl^−^ and NO_3_^−^ translocation, to moderate‐to‐high NO_3_^−^/Cl^−^ selectivity, to exceptionally high NO_3_^−^ selectivity, and ultimately to exclusive water transport.

## 3 Conclusion

To conclude, we have designed and synthesized simple cyanostilbene‐based C‐H HB donor podands and tuned their anion‐binding mode and strength to combine efficient water permeability with modulated anion transport through dynamic and adaptive assemblies. To the best of our knowledge, this work provides the first evidence that CH‐donor molecular podands can form adaptive and transient water‐templated self‐assembled aggregates in lipid bilayers that sustain efficient water translocation via weak CH···OH_2_ interactions. This supports the use of CH binding sites for the design of AWCs for applications in water purification. Weakening the HB donor strength and tailoring the geometry of the binding site also suppresses chloride transport, due to insufficient compensation of its dehydration penalty, while still supporting efficient nitrate transport, consistent with the lower dehydration energy of nitrate. This provides a straightforward strategy for achieving nitrate selectivity and should encourage further work in the field toward biomimetic nitrate transporters. Moreover, together with very recent work from the Valkenier group [56], this study highlights how precise modulation of HB donor strength can shift transport behavior from efficient chloride translocation to nitrate‐selective transport and ultimately to complete suppression of ion transport, enabling selective water translocation. Altogether, this study shows that structural self‐assembled channels stabilized by strong HB are not always optimal, and emerging polymorphic superstructures based on weaker interactions can generate effective water/ion conducting pathways, providing important insights into their transport mechanism and permselectivity.

## Author Contributions

**Ioan Stroia**: investigation, writing – original draft, methodology, validation, and formal analysis. **Dan‐Dan Su**: methodology, investigation. **Yuhao Li**: methodology. **Niculina Hadade**: investigation, validation, writing – review and editing, and funding acquisition. **Ion Grosu**: writing – review and editing, investigation, and funding acquisition. **Arie van der Lee**: methodology, formal analysis. **Mihail Barboiu**: conceptualization, investigation, funding acquisition, writing – review and editing, validation, formal analysis, and project administration.

## Conflicts of Interest

The authors declare no conflicts of interest.

## Supporting information




**Supporting File 1**: anie72570‐sup‐0001‐SuppMat.docx.

## Data Availability

The data that support the findings of this study are available from the corresponding author upon reasonable request.
