# Supplementary material for: Selective Nitrate Transmembrane Transport Through Adaptive Weak C─H Bonding Cyanostilbene Water Channels
Source: Angew Chem Int Ed Engl. 2026 May 11;65(29):e4918548. doi: 10.1002/anie.4918548 (PMC13360724; doi:10.1002/anie.4918548)
Supplement: Supplementary file 1 — Supporting File 1: anie72570‐sup‐0001‐SuppMat.docx. [file ANIE-65-e4918548-s001.docx]

Supporting Information for

Selective Nitrate Transmembrane Transport Through Adaptive Weak C-H bonding Cyanostilbene Water Channels

Ioan Stroia,^[a,b]^ Dandan Su,^[a]^ Yuhao Li,^[a]^ Niculina Hadade,^[b]^ Ion Grosu,^[b]^ Arie van der Lee,^[a]^ and Mihail Barboiu^[a]^*

[a] Institut Européen des Membranes, Adaptive Supramolecular Nanosystems Group, University of Montpellier, ENSCM-CNRS, UMR5635, Place E. Bataillon CC047, 34095 Montpellier, France

[b] Babes-Bolyai University, Supramolecular Organic and Organometallic Chemistry Center (SOOMCC), Cluj-Napoca, 11 Arany Janos str., RO-400028, Cluj-Napoca, Romania

Table of Contents

[General experimental data 2](#_Toc227318808)

[Synthesis and characterization 2](#_Toc227318809)

[X-Ray crystallography 10](#_Toc227318810)

[Water Transport tests 12](#_Toc227318811)

[Molecular Dynamics (MD) simulations 16](#_Toc227318812)

[Ion Transport tests 17](#_Toc227318813)

[Carboxyfluorescein (CF) leakage assay 35](#_Toc227318814)

[Planar bilayer experiments 36](#_Toc227318815)

[DPPC experiments 39](#_Toc227318816)

[Cholesterol-dependent tests 40](#_Toc227318817)

[Variable-temperature nitrate transport experiments 42](#_Toc227318818)

[^1^H-NMR titration experiments 47](#_Toc227318819)

[DFT calculations 61](#_Toc227318820)

[References 65](#_Toc227318821)

# General experimental data

All reagents and solvents were obtained from commercial sources, including Sigma Aldrich and VWR and were used without further purification. Oil bath was used for all reactions requiring heating. The NMR spectra were recorded on a Bruker Avance III 400 MHz. Fluorescence spectra were recorded using a Perkin Elmer FL6500 spectrometer equipped with a fast filter and thermostat.

# Synthesis and characterization

**General procedure for the synthesis of the studied CH donors**

All the compounds have been synthetized (Scheme S1) according to the following general procedure: 1,3-phenylenediacetonitrile (1.92 mmol), the appropriate aldehyde (4.04 mmol) and NaOH (0.4 mmol) were solubilized in absolute ethanol (20 mL) and water (0.2 mL). The reaction mixture was refluxed overnight. After cooling to room temperature. the precipitates were filtered and washed with cold EtOH. Compound **CH1** was purified by precipitation with diethyl ether from toluene, while **CH2**, **CH3**, **CH5** and **CH6** were subjected to trituration with toluene.

Scheme S1. Synthesis of the target C-H donor transporters.

**(2Z,2'Z)-2,2'-(1,3-phenylene)bis(3-phenylacrylonitrile)**, **CH1.** Yield: 344 mg, 54%. ^1^H-NMR (400 MHz, DMSO-*d*_6_) δ 8.20 (s, 2H), 8.11 (t, *J* = 1.7 Hz, 1H), 7.97 (d, *J* = 6.9 Hz, 4H), 7.84 (dd, *J* = 7.8, 1.8 Hz, 2H), 7.71 – 7.65 (m, 1H), 7.61 – 7.52 (m, 6H). ^13^C-NMR (101 MHz, DMSO-*d*_6_) δ 144.28, 134.87, 133.60, 130.89, 130.10, 129.24, 129.03, 126.72, 123.08, 117.74, 109.72.


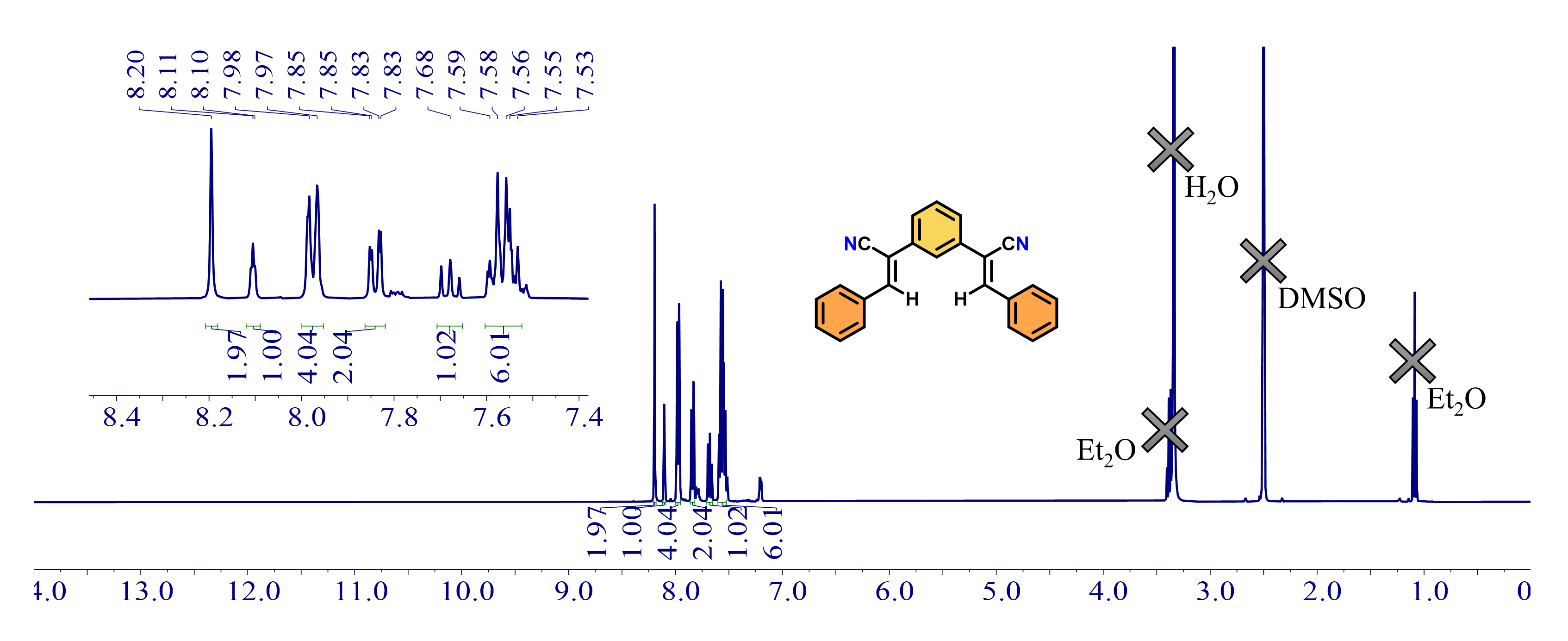


Figure S1. ^1^H-NMR spectrum (400 MHz, DMSO-*d6*) of transporter **CH1.**


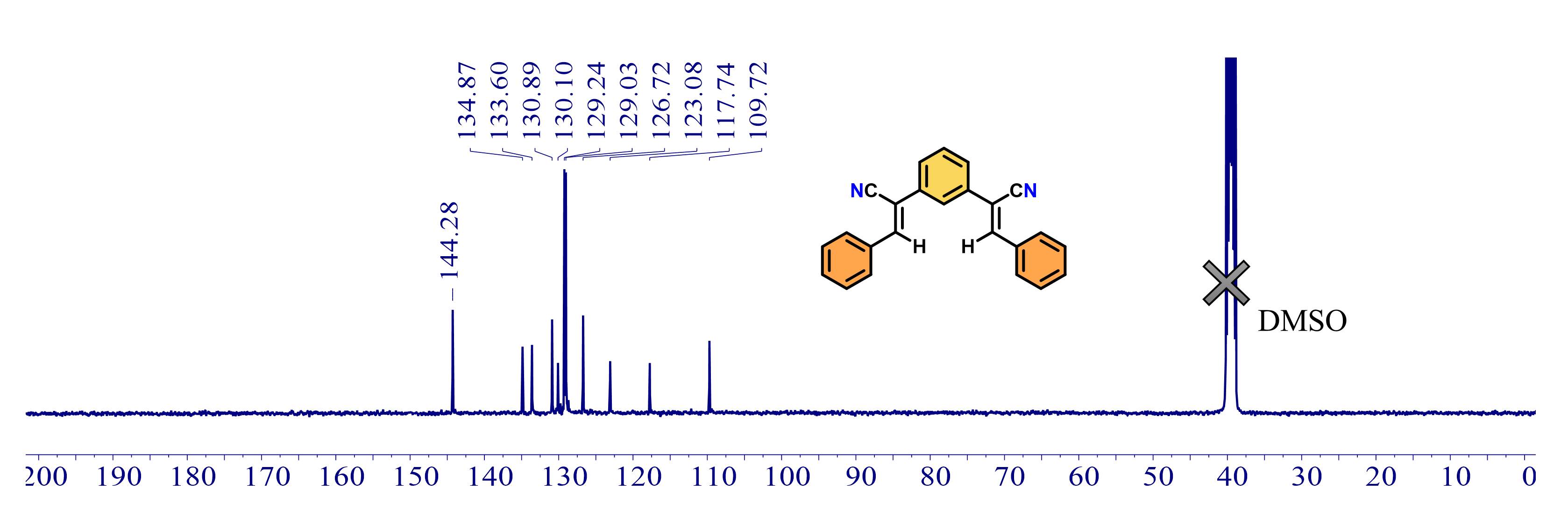


Figure S2. ^13^C-NMR spectrum (400 MHz, DMSO-*d6*) of transporter **CH1.**

**(2Z,2'Z)-2,2'-(1,3-phenylene)bis(3-(4-nitrophenyl)acrylonitrile)**, **CH2**. Yield: 567 mg, 70%. ^1^H-NMR (400 MHz, DMSO-*d*_6_) δ 8.41 (d, *J* = 8.8 Hz, 4H), 8.35 (s, 2H), 8.21 – 8.15 (m, 5H), 7.93 (dd, *J* = 7.8, 1.5 Hz, 2H), 7.74 (t, *J* = 7.9 Hz, 1H). ^13^C-NMR (101 MHz, DMSO-*d*_6_) δ 148.01, 141.98, 139.78, 134.34, 130.34, 127.74, 124.23 – 124.05 (two overlapped singlets), 123.80, 117.00, 113.38.


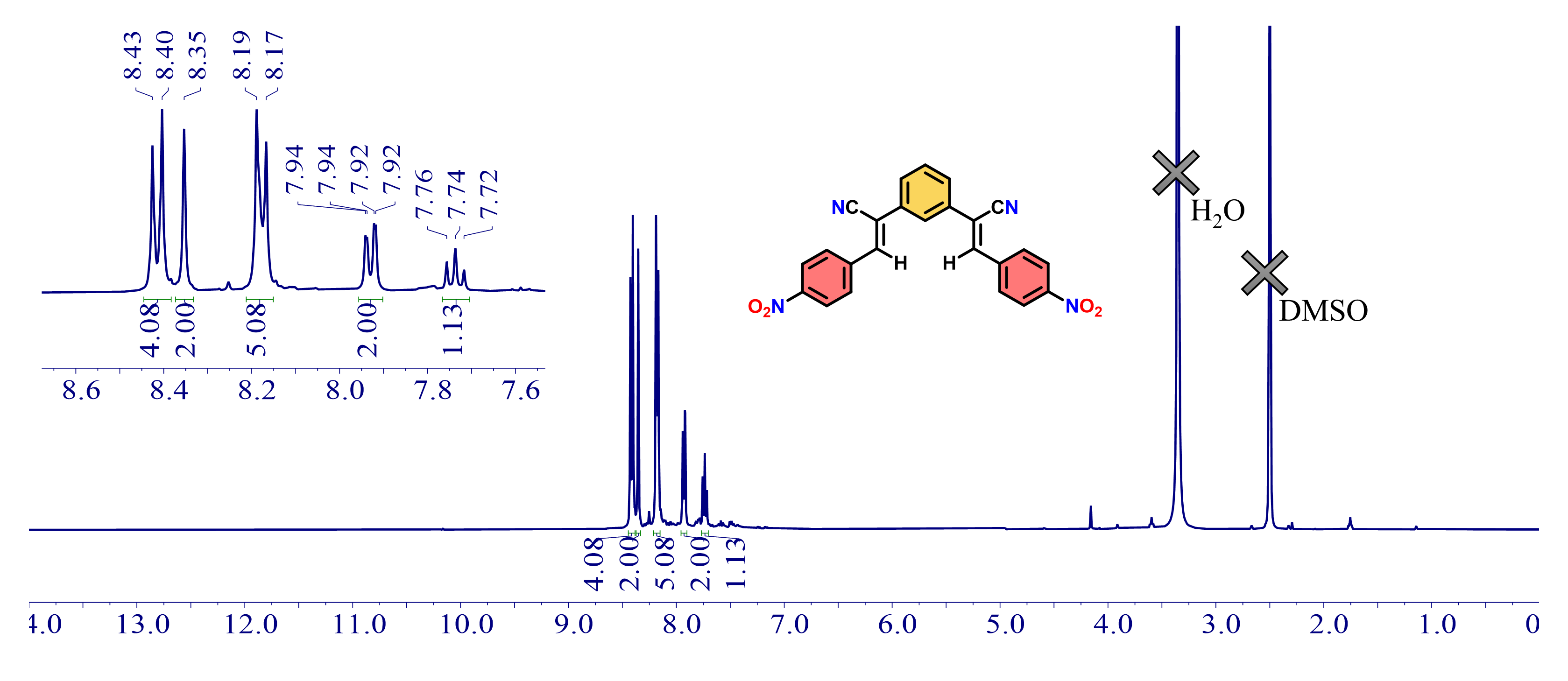


Figure S3. ^1^H-NMR spectrum (400 MHz, DMSO-*d6*) of transporter **CH2**.


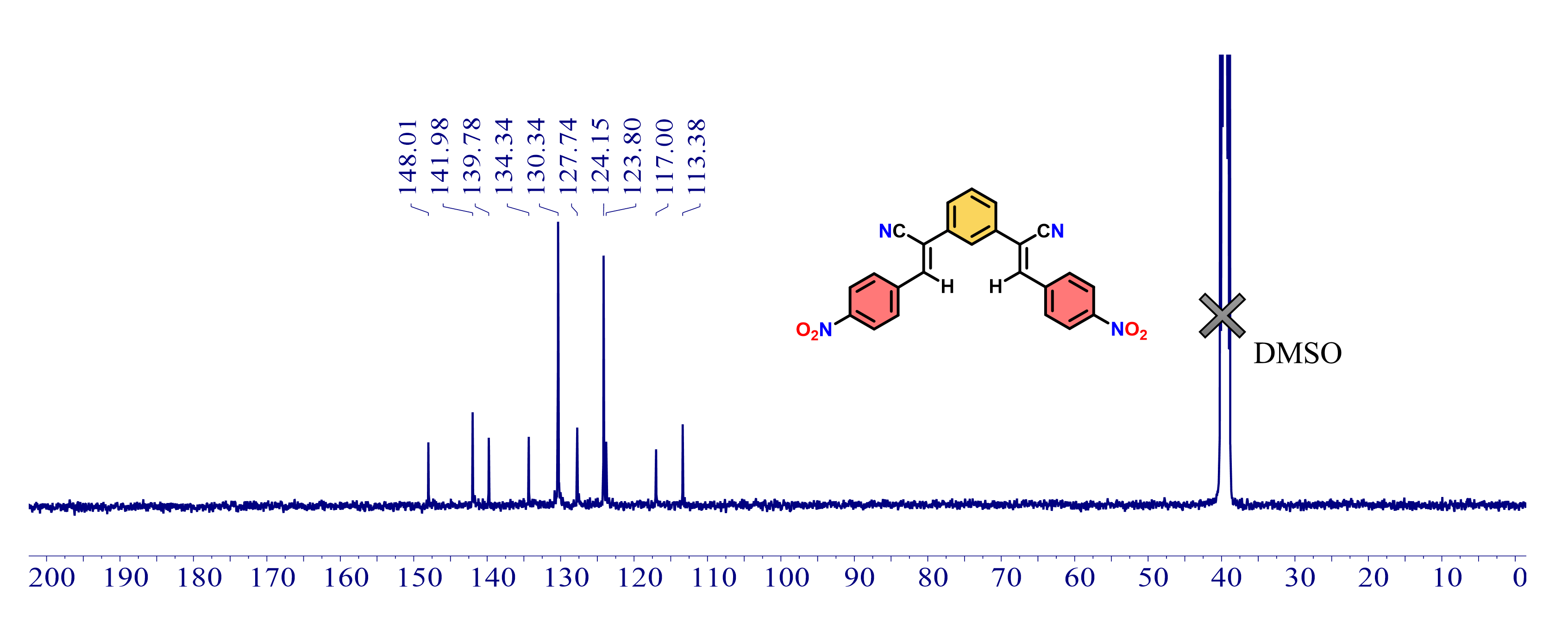


Figure S4. ^13^C-NMR spectrum (400 MHz, DMSO-*d6*) of transporter **CH2.**

**(2Z,2'Z)-2,2'-(1,3-phenylene)bis(3-(4-butoxyphenyl)acrylonitrile)**, **CH3**. Yield: 650 mg, 71%. ^1^H-NMR (400 MHz, DMSO-*d*_6_) δ 8.03 (s, 2H), 8.00 – 7.92 (m, 5H), 7.73 (d, *J* = 7.6 Hz, 2H), 7.60 (t, *J* = 7.8 Hz, 1H), 7.10 (d, *J* = 8.6 Hz, 4H), 4.05 (t, *J* = 6.3 Hz, 4H), 1.71 (p, *J* = 6.5 Hz, 4H), 1.44 (h, *J* = 7.1 Hz, 4H), 0.94 (t, *J* = 7.3 Hz, 6H). ^13^C-NMR (101 MHz, DMSO-*d*_6_) δ 160.86, 143.64, 135.24, 131.36, 129.96, 125.97, 125.87, 122.41, 118.33, 114.96, 106.28, 67.54, 30.64, 18.72, 13.70.


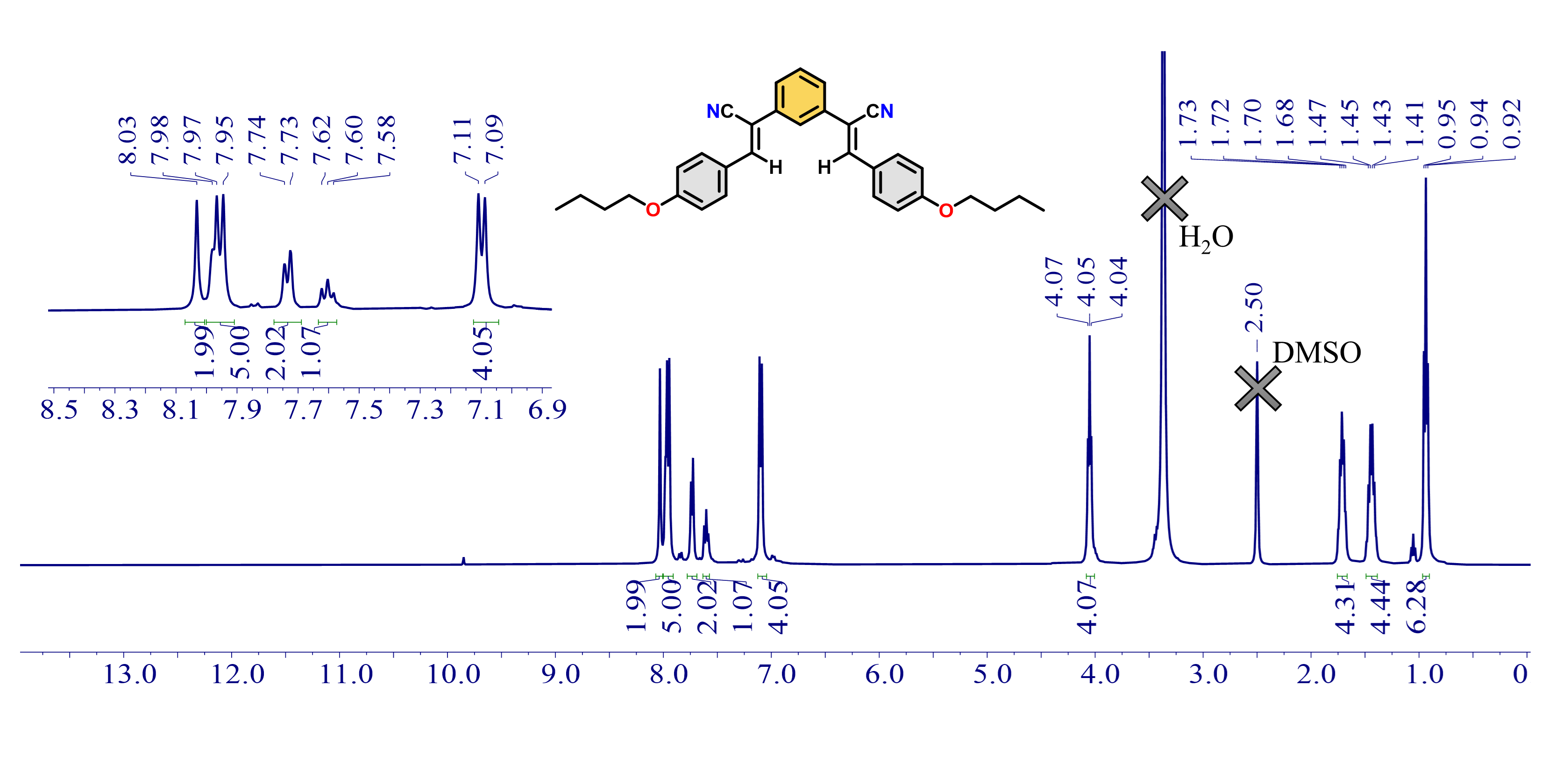


Figure S5. ^1^H-NMR spectrum (400 MHz, DMSO-*d6*) of transporter **CH3**.


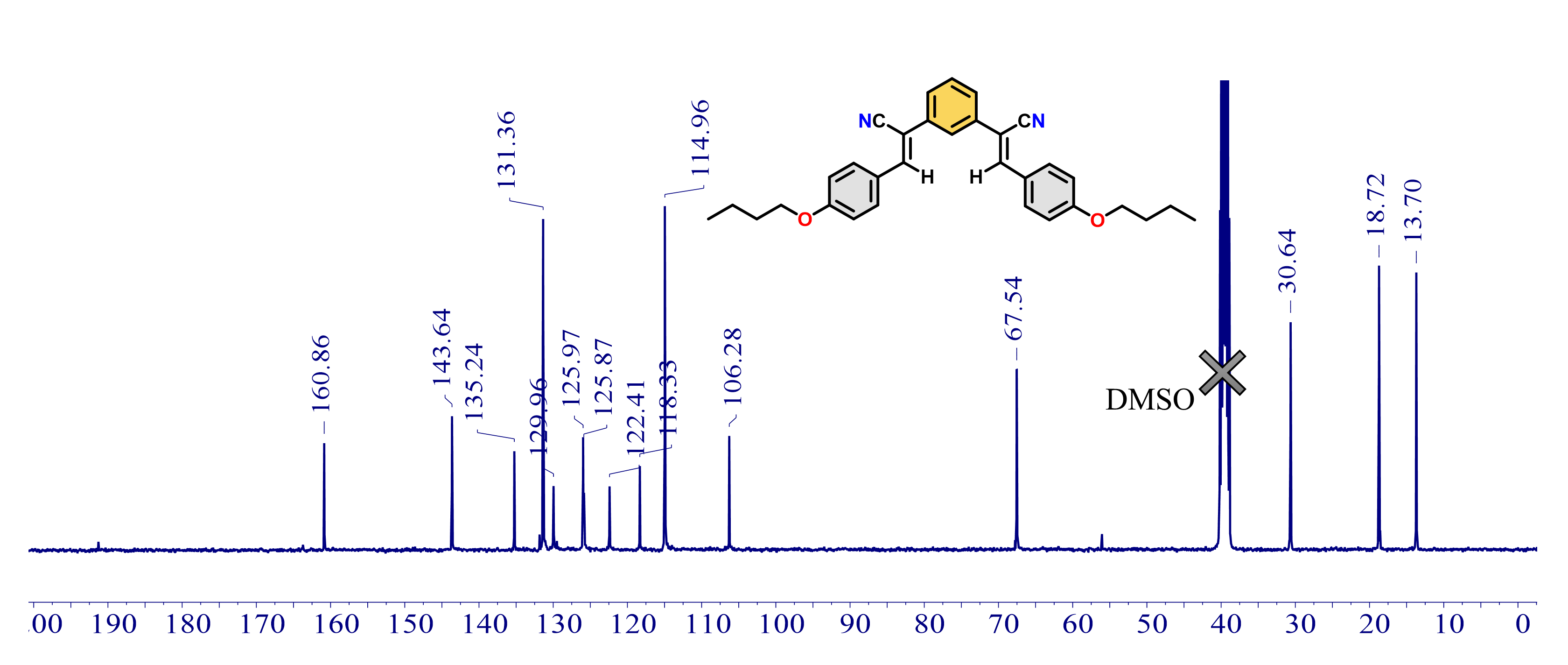


Figure S6. ^13^C-NMR spectrum (400 MHz, DMSO-*d6*) of transporter **CH3.**

**(2Z,2'Z)-2,2'-(1,3-phenylene)bis(3-(pyridin-3-yl)acrylonitrile)**, **CH4**. Yield: 325 mg, 51%. ^1^H-NMR (400 MHz, DMSO-*d*_6_) δ 9.03 (d, *J* = 2.3 Hz, 2H), 8.69 (dd, *J* = 4.8, 1.6 Hz, 2H), 8.40 (dt, *J* = 8.1, 2.0 Hz, 2H), 8.24 (s, 2H), 8.12 (t, *J* = 1.7 Hz, 1H), 7.88 (dd, *J* = 7.8, 1.8 Hz, 2H), 7.71 (t, 1H), 7.61 (dd, *J* = 8.0, 4.8 Hz, 2H). ^13^C-NMR (101 MHz, DMSO-*d*_6_) δ 151.13, 150.59, 141.06, 135.29, 134.45, 130.23, 129.68, 127.14, 123.95, 123.29, 117.31, 111.90.


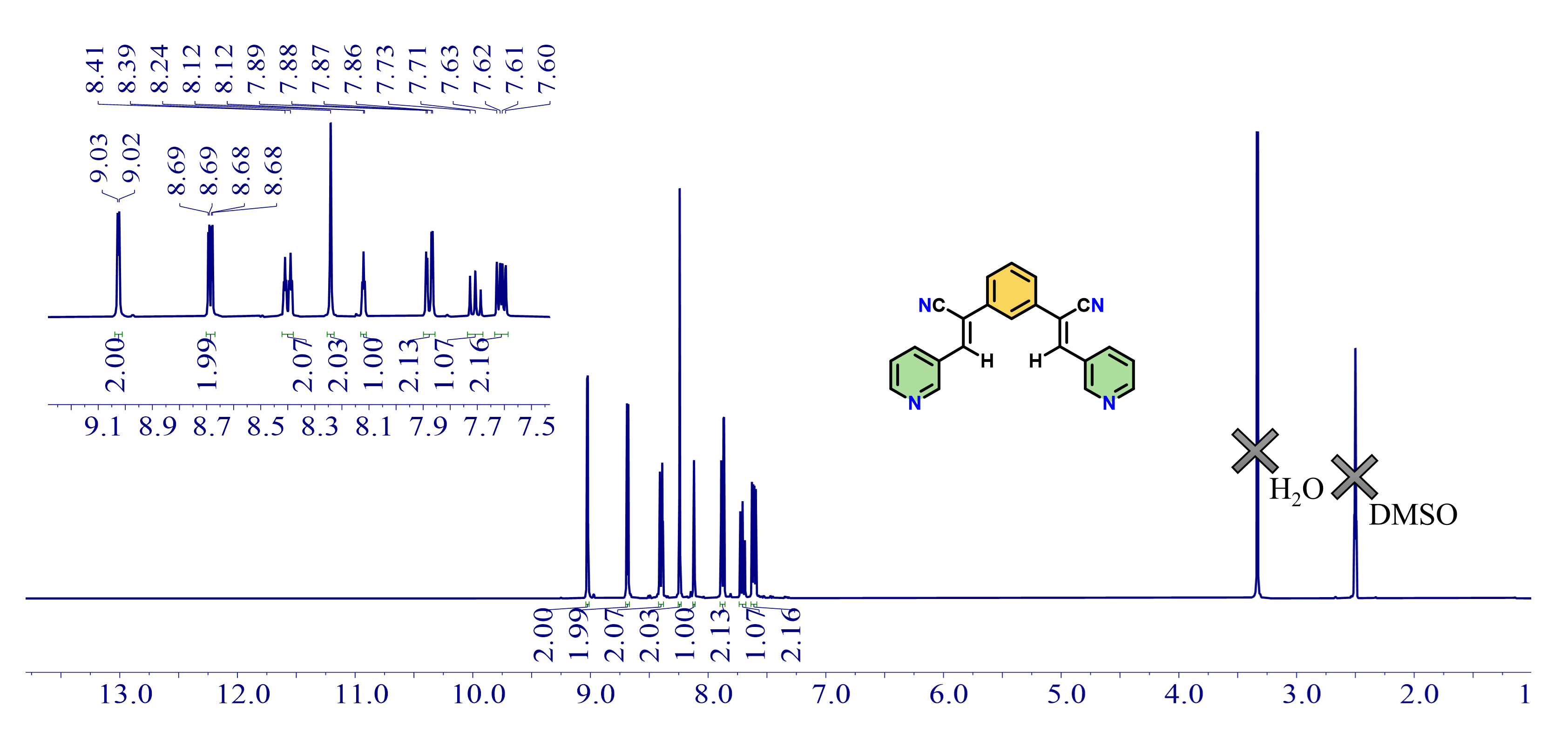


Figure S7. ^1^H-NMR spectrum (400 MHz, DMSO-*d6*) of transporter **CH4**.


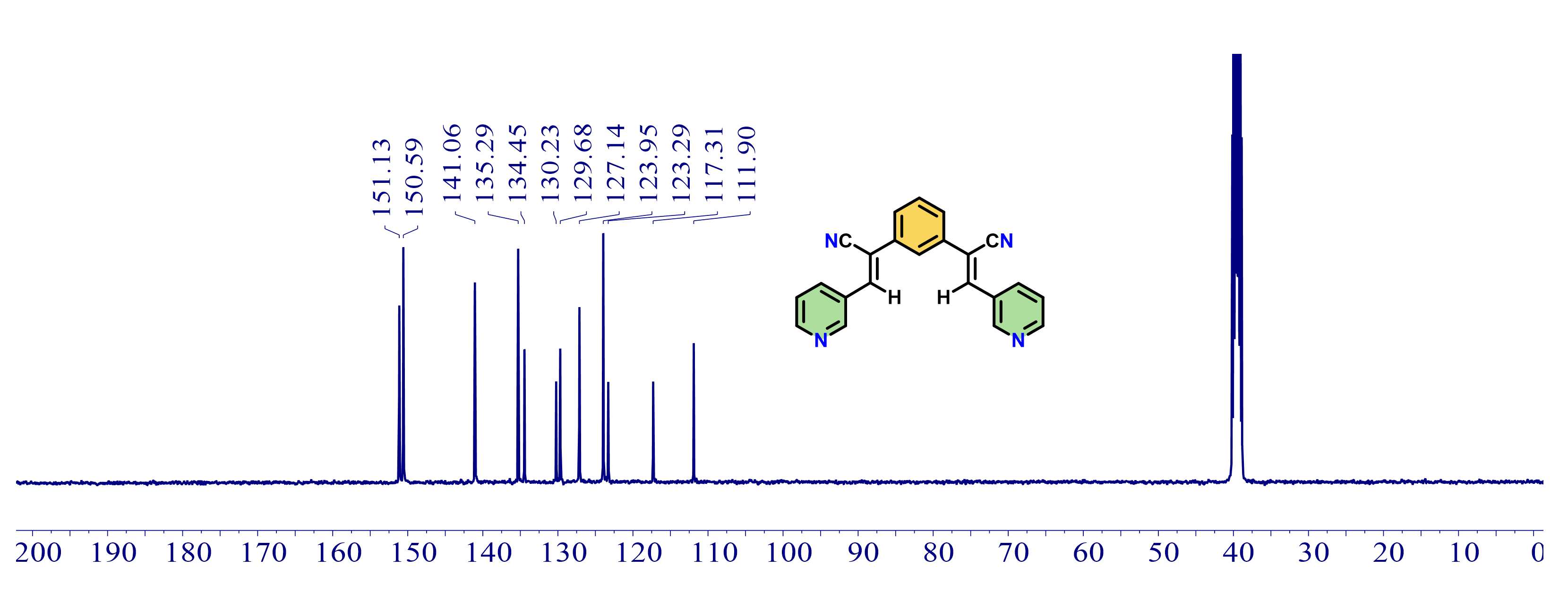


Figure S8. ^13^C-NMR spectrum (400 MHz, DMSO-*d6*) of transporter **CH4.**

**(2Z,2'Z)-2,2'-(1,3-phenylene)bis(3-(1H-imidazol-2-yl)acrylonitrile)**, **CH5**. Yield: 300 mg, 50%. ^1^H-NMR (400 MHz, DMSO-*d*_6_) δ 7.86 (s, 1H), 7.68 – 7.64 (overlapped signals, 4H), 7.58 (t, *J* = 7.0 Hz, 1H), 7.32 (overlapped signals, 4H), 3.88 (s, broad). ^13^C-NMR (101 MHz, DMSO-*d*_6_) δ 143.43, 135.43, 131.17, 130.11, 127.31, 125.30, 121.60, 117.81, 104.78.


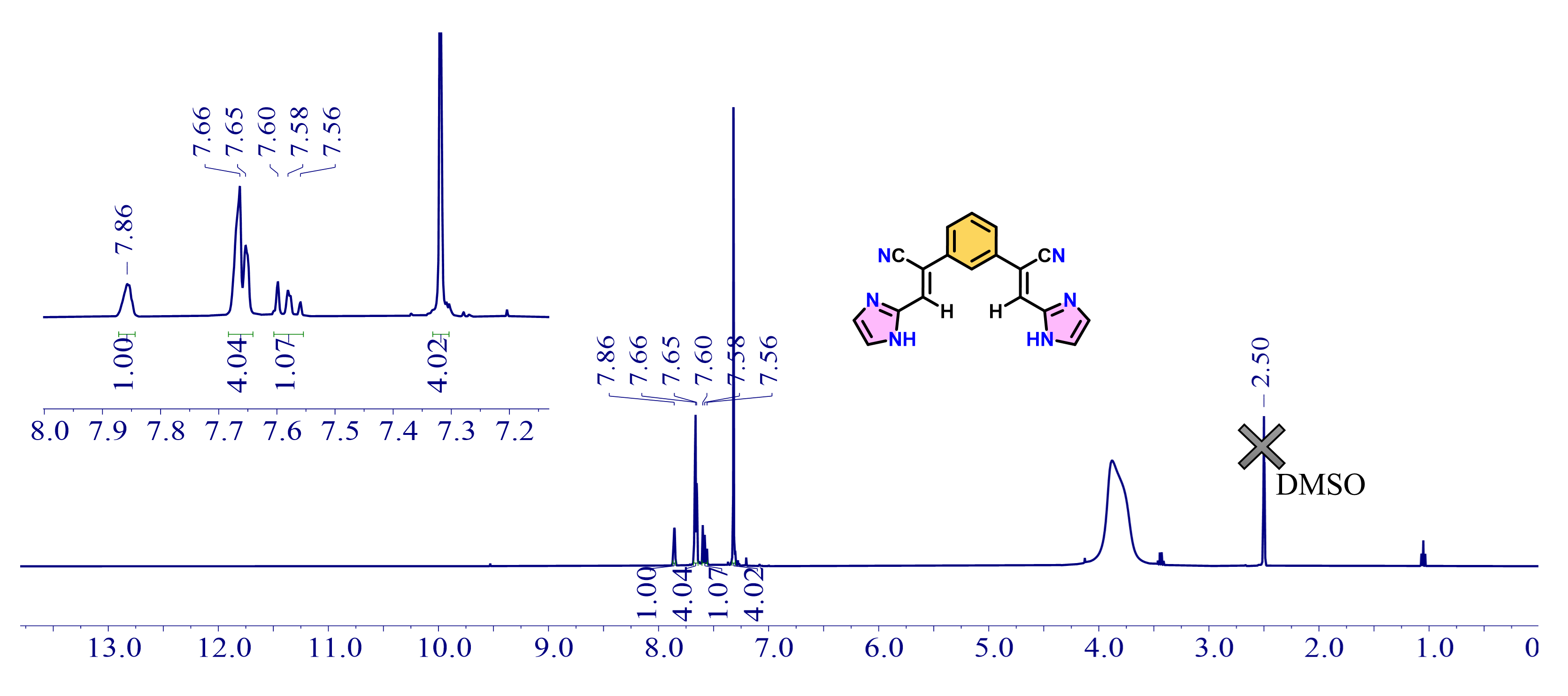


Figure S9. ^1^H-NMR spectrum (400 MHz, DMSO-*d6*) of transporter **CH5**.


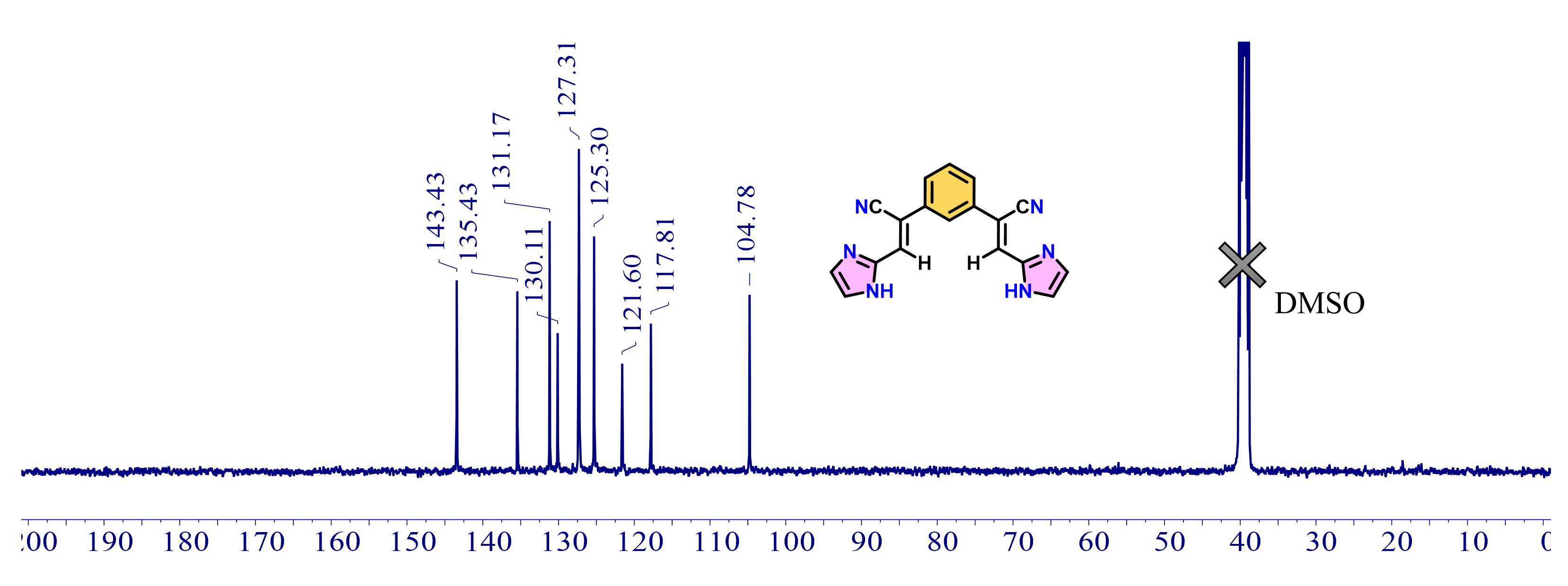


Figure S10. ^13^C-NMR spectrum (400 MHz, DMSO-*d6*) of transporter **CH5.**

**(2Z,2'Z)-2,2'-(1,3-phenylene)bis(3-(1H-imidazol-4-yl)acrylonitrile)**, **CH6**. Yield: 450 mg, 75%. ^1^H-NMR (400 MHz, DMSO-*d*_6_) δ 7.98 – 7.92 (two overlapped singlets, 3H), 7.90 (s, 2H), 7.84 (s, 2H), 7.67 (d, *J* = 7.3 Hz, 2H), 7.56 (t, *J* = 7.5 Hz, 1H). ^13^C-NMR (101 MHz, DMSO-*d*_6_) δ 137.65, 136.09, 135.24, 135.18, 129.92, 125.02, 122.76, 121.41, 118.34, 103.96.


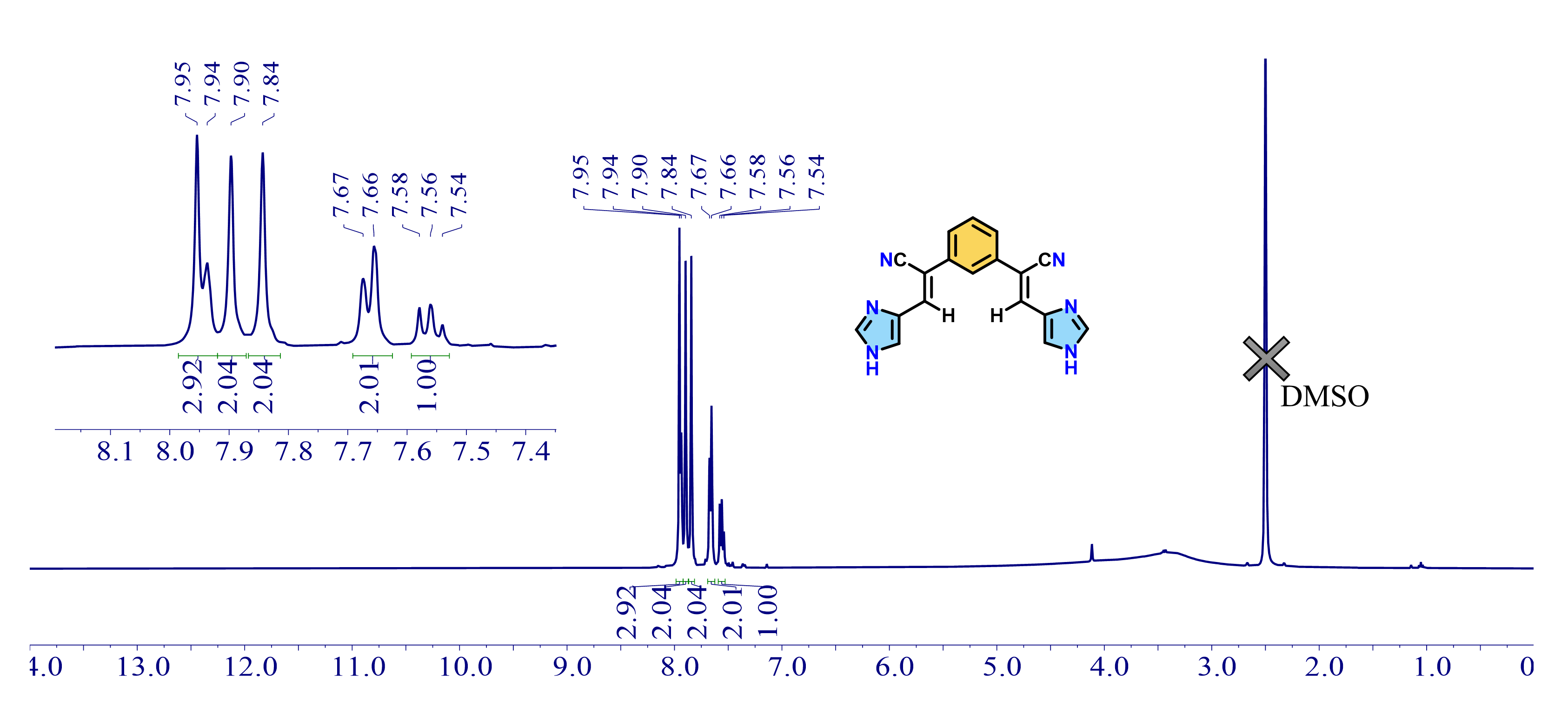


Figure S11. ^1^H-NMR spectrum (400 MHz, DMSO-*d6*) of transporter **CH6**.


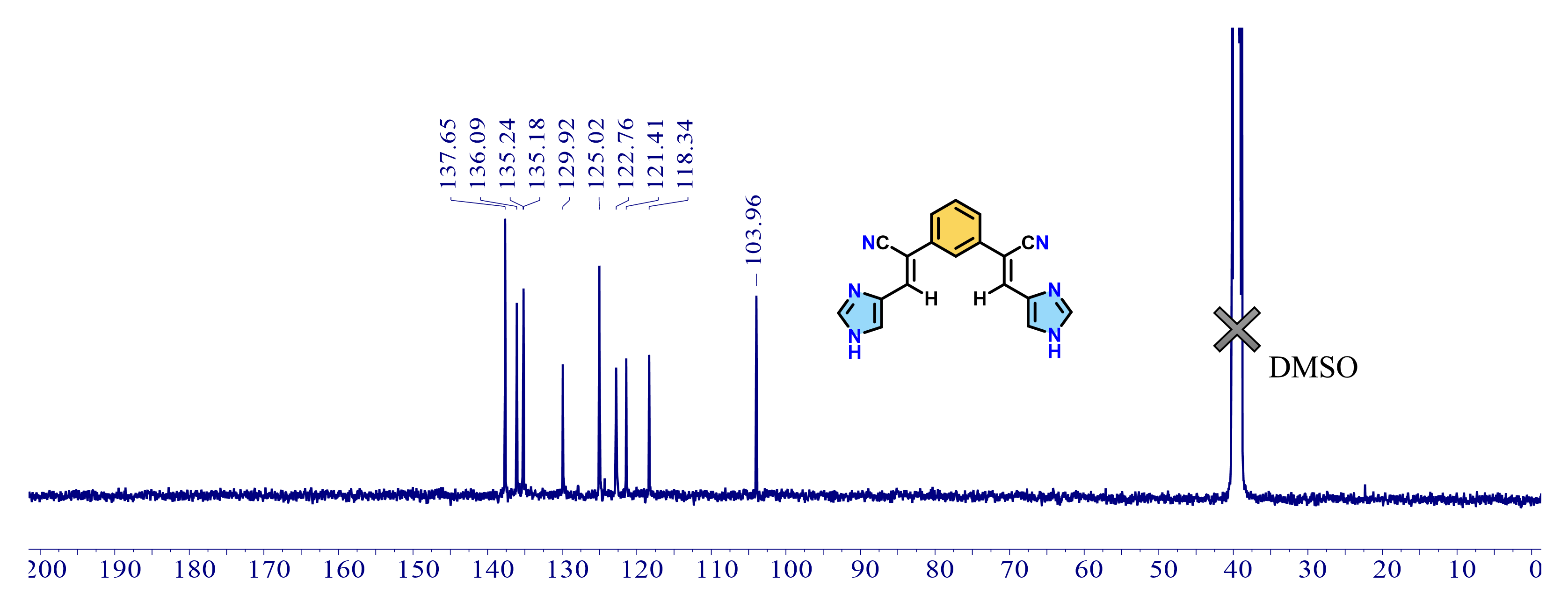


Figure S12. ^13^C-NMR spectrum (400 MHz, DMSO-*d6*) of transporter **CH6.**

**Procedure for the synthesis of the NH1 transporter**

**NH1** anionophore was synthesized (Scheme S2) according to the following procedure: A solution of isophthaloyl chloride (10 mmol) in acetonitrile (10 mL) was added dropwise over a solution of aniline (22 mmol) in acetonitrile (50 mL), and the reaction mixture was heated to reflux overnight. After cooling to room temperature, the formed precipitate was filtered, washed with acetonitrile and dried in vacuum.

Scheme S2. Synthesis of NH1 transporter.

**Isophthaloyl dichloride-*N^1^,N^3^*-diphenylisophthalamide-acetonitrile-aniline-*λ^1^*-azane (1/1/1/1/1), NH1.** Yield: 3 g, 95%. ^1^H NMR (400 MHz, DMSO) δ 10.44 (s, 2H), 8.55 (dd, *J* = 4.2, 1.9 Hz, 1H), 8.15 (d, *J* = 7.8 Hz, 2H), 7.81 (d, *J* = 6.6 Hz, 4H), 7.70 (t, *J* = 7.7 Hz, 1H), 7.42 – 7.33 (m, 4H), 7.12 (t, *J* = 7.4 Hz, 2H).


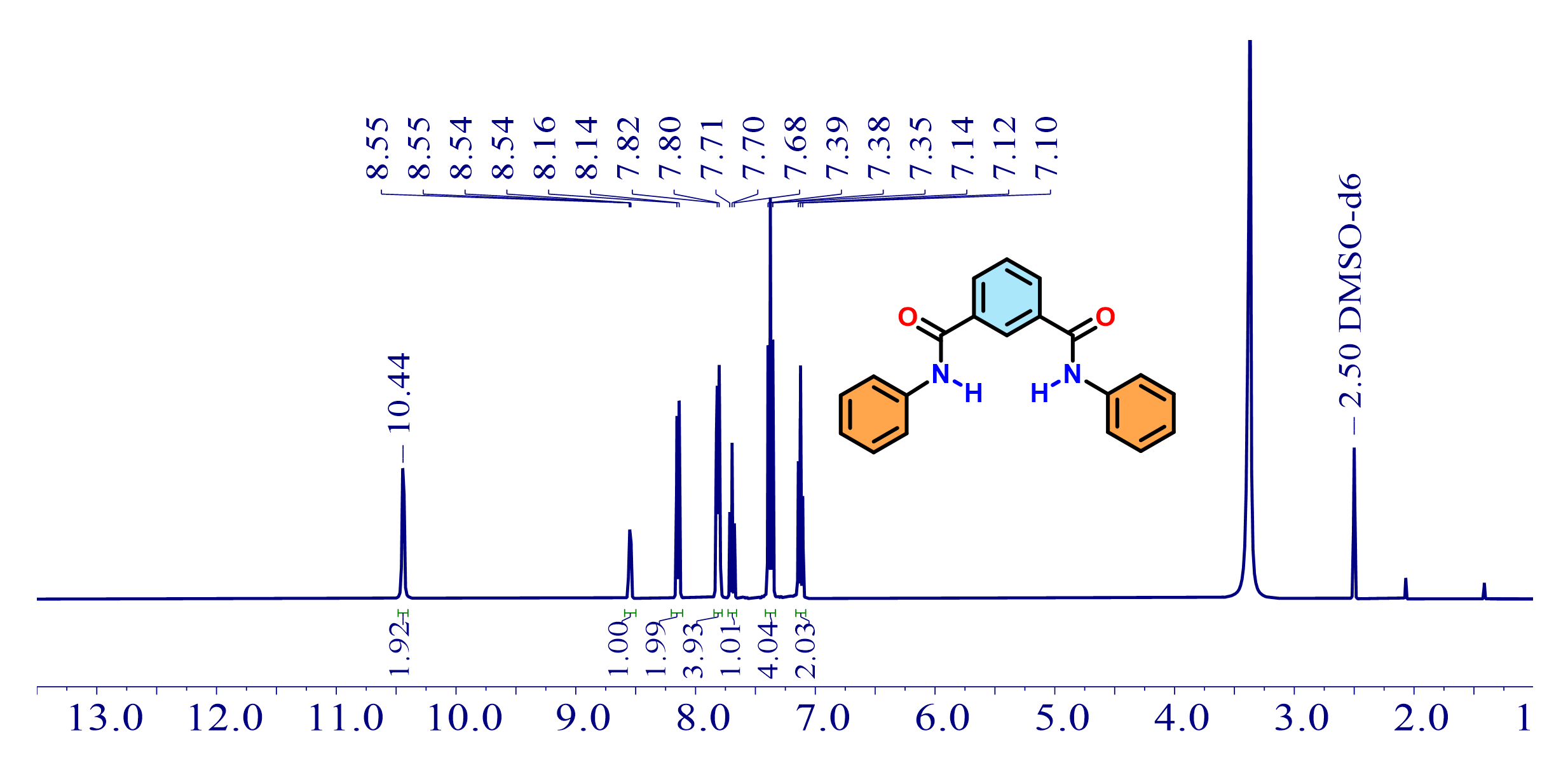


Figure S13. 1H NMR spectrum (400 MHz, DMSO-*d6*) of transporter **NH1**.

# X-Ray crystallography

The X-ray single-crystal structural data were collected at 173K on a Bruker D8 VENTURE equipped with a PHOTON II CPAD detector and a microfocus X-ray source with Cu-Kα radiation (λ = 1.54184 Å) operating at 50 kV and 1.1 mA. The frames were integrated with the Bruker SAINT software package using a narrow-frame algorithm.^[1]^ The structures were solved using Superflip^[2]^ employing default algorithm parameters.^[3]^ The structure refinement was carried out using CRYSTALS^[4]^ employing spherical scattering factors for all atoms on all reflections with *I*>-3σ(*I*). A possible pseudo-symmetry was investigated but found not to be present; no clear signs of twinning were observed.

|  | CH4⸧H2O |
| --- | --- |
| formula | C_22_H_16_N_4_O |
| moiety | C_22_H_14_N_4_,H_2_O |
| *T* (K) | 173 |
| spacegroup | *P*2_1_*/c* |
| crystal system | Monoclinic |
| *a* (Å) | 8.1579(6) |
| *b* (Å) | 34.103(3) |
| *c* (Å) | 12.6578(10) |
| *α* (º) | 90 |
| *β* (º) | 90.770(4) |
| *γ* (º) | 90 |
| *V* (Å^3^) | 3521.2(5) |
| *Z* | 8 |
| *ρ* (gcm^-3^) | 1.329 |
| *M*_r_ (gmol^-1^) | 352.39 |
| *μ* (mm^-1^) | 0.678 |
| *R*_int_ | 0.054 |
| *Θ*_max_ (º) | 67.124 |
| resolution (Å) | 0.84 |
| *N*_tot_ (measured) | 41106 |
| *N*_ref_ (unique) | 6187 |
| *N*_ref_ (*I*>2*σ*(*I*)) | 5604 |
| *N*_ref_ (least-squares) | 6187 |
| *N*_par_ | 503 |
| <*σ*(*I*)/*I*> | 0.0382 |
| *R*_1_ (*I*>2*σ*(*I*)) | 0.1441 |
| *wR*_2_ (*I*>2*σ*(*I*)) | 0.3149 |
| *R*_1_ (all) | 0.1483 |
| *wR*_2_ (all) | 0.3160 |
| GOF | 0.9120 |
| Δ*ρ* (eÅ^-3^) | -0.64/0.57 |
| crystal size (mm^3^) | 0.09x0.09x0.26 |

**
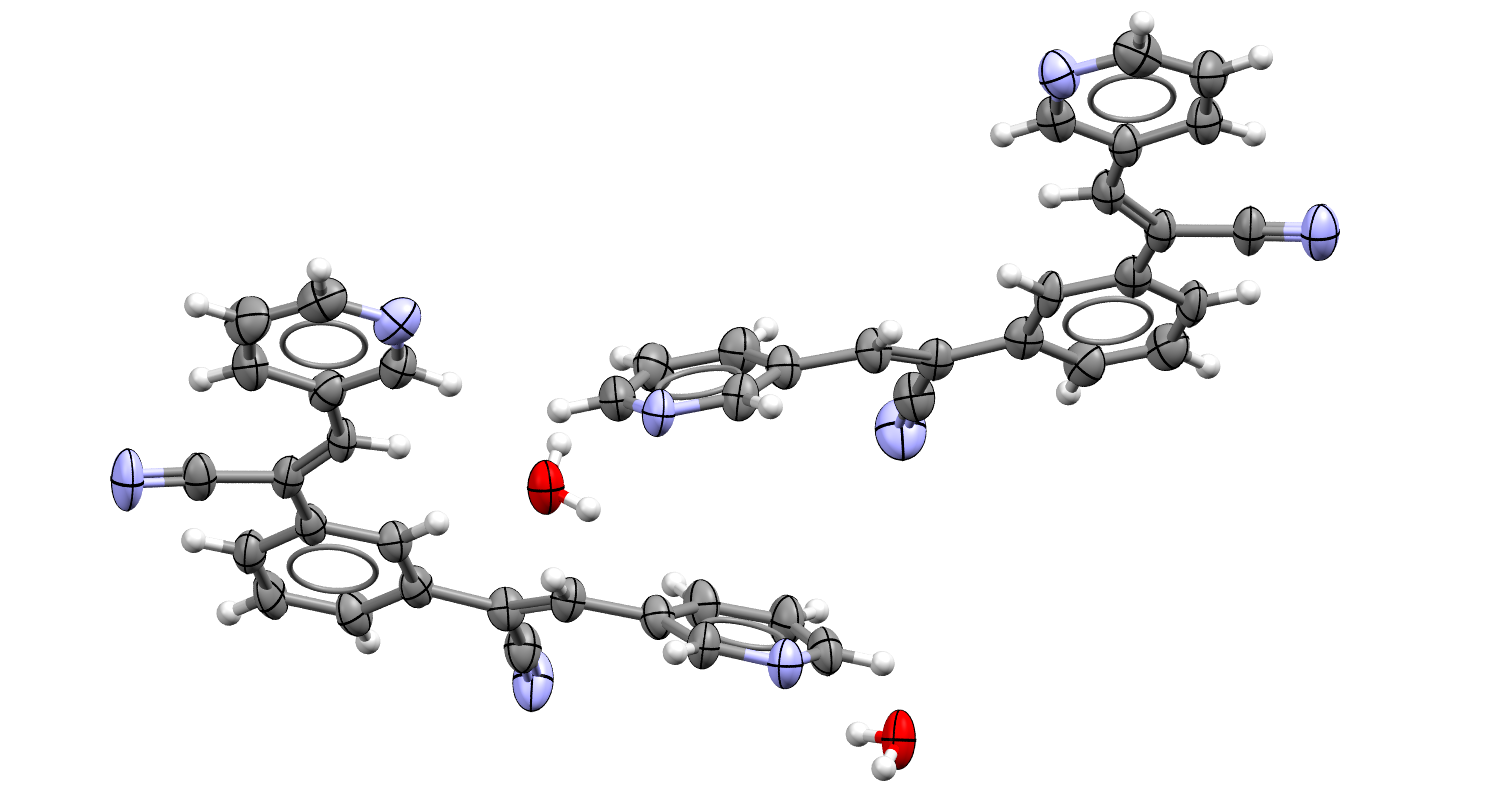
**

Figure S14. Molecular structure of the asymmetric unit of **CH4⸧H_2_O** in the solid state (50% probability level).

CCDC 2527308 (**CH4⸧H2O**) contains the supplementary crystallographic data for **CH4⸧H2O**. These data can be obtained free of charge from The Cambridge Crystallographic Data Centre via [www.ccdc.cam.ac.uk/structures](http://www.ccdc.cam.ac.uk/structures).

# Water Transport tests

**General procedure for the preparation of EYPC/PS/Cholesterol large unilamellar vesicles (LUVs)**

A mixture of PC/PS/Chl with a molar ratio of 4/1/5 in chloroform was added to a 10 mL round-bottomed flask, the solvent was slowly removed under a flow of argon, and the resulting thin film was further dried in a vacuum desiccator for a minimum of 3 h. The lipid film was hydrated with 1000 μL of PBS buffer (10 mM, pH = 6.4) containing 200 mM sucrose for 1h under gentle vertexing. Afterwards, the suspension was submitted to 10 freeze-thaw cycles (bathed in liquid nitrogen and water at 30 °C, respectively). The large multilamellar liposome suspension (1000 μL) was submitted to extrusion (21 extrusions) at room temperature through a 100 nm polycarbonate membrane to afford a suspension of LUVs with an average diameter of around 100 nm (as confirmed by dynamic light scattering measurements).

**Light scattering measurements to quantify the water transport**

The water permeability tests were conducted on a stopped-flow instrument (SFM3000 + MOS450, Bio-Logic SAS, Claix, France). The vesicles have been exposed to a hypertonic osmolyte (400 mM sucrose) in the same PBS buffer solution, resulting in the shrinkage of the LUVs due to an outwardly directed osmotic gradient. It should be noted that the extravesicular osmolarity at time t = 0, immediately after mixing the 200 and 400 mOsm sucrose solutions, is 300 mOsm, whereas the intravesicular osmolarity remains 200 mOsm. This creates an initial osmotic gradient of 100 mOsm between the external and internal media, which drives LUV shrinkage. The resulting vesicle volume changes were monitored by measuring variations in light scattering at an excitation wavelength of 345 nm.

For the stopped-flow experiments, 100 μL of the above-prepared stock lipid solution was diluted with 1880 μL of 200 mM sucrose in 10 mM PBS buffer solution. The compounds were injected in 20 μL aliquots in DMSO. The solution mix was thermostated at 20℃ for 30 min before exposure to hypertonic 400 mM sucrose solutions. Experiments have been conducted at two different molar compound-to-lipid ratio (mCLR%) concentrations (1.860 and 3.953 mol%, respectively). The experiments have been triplicated to obtain the average net permeability, which has been calculated as explained below.

According to the Rayleigh-Gans theory, the abrupt change of the vesicle size leads to variation in the light scattering at 90°, which could be fitted in the form of two exponential functions (Equation 1). The osmotic permeability (*P_f_*) was calculated according to the equation 2, where k_1_ is the exponential coefficient of the change in the light scattering; S and V_0_ are the initial surface area and volume of the vesicles (S/V_0_=60 µm^-1^); V_w_ is the molar volume of water (18*10^-6^ m^3^ mol^-1^) and Δ_osm_ is the osmolarity difference (100 Osm m^-3^). Net water permeability (Net*P_f_*) was calculated according to relation 3 and expressed in μm s^-1^.

$$Y=at+b+c_{1}e^{-k_{1}t}+c_{2}e^{-k_{2}t}(1)$$

$$P_{f}=\frac{k_{1}}{\frac{S}{V_{0}}+V_{w}+\Delta_{osm}} (2)$$

$${NetP}_{f}=P_{f}-P_{f}\left( control \right) (3)$$

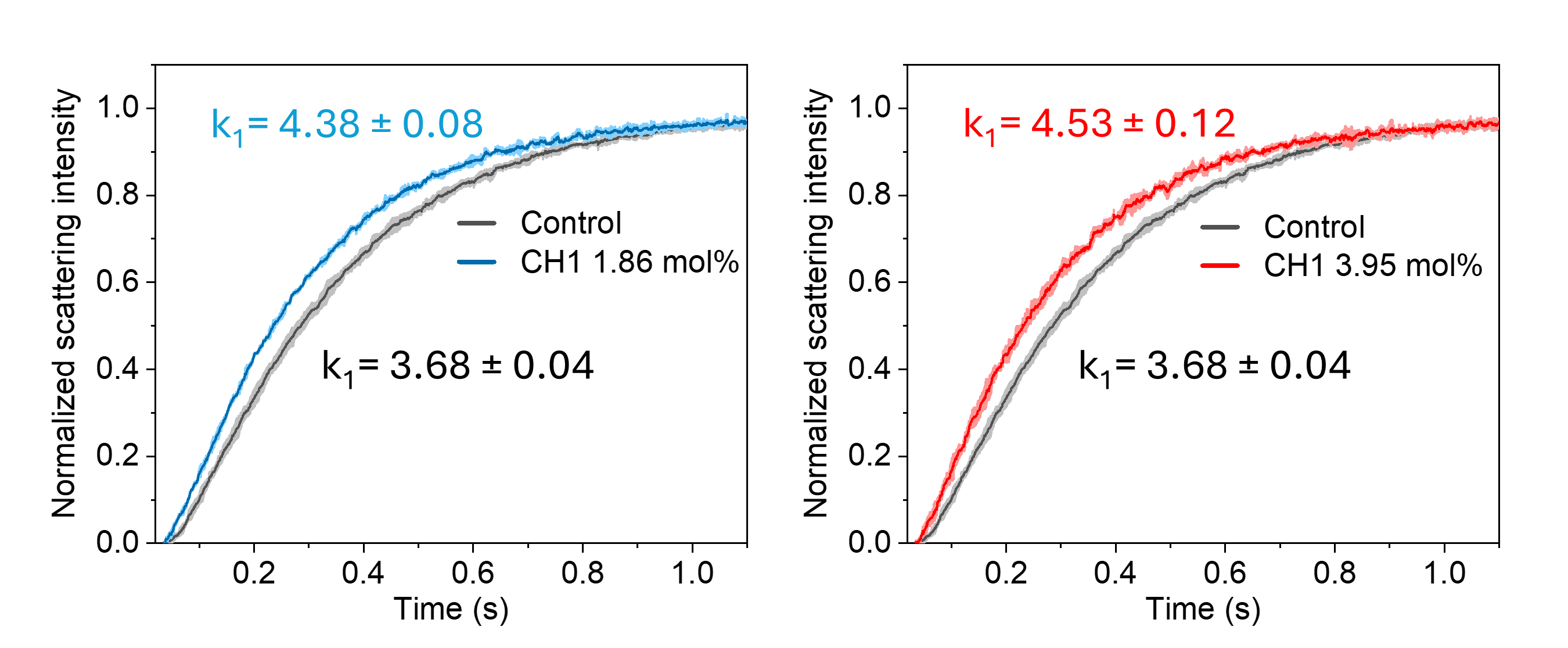


Figure S15. Stopped-flow light-scattering traces of LUVs in the absence and presence of **CH1** at 1.86 mol% and 3.95 mol%, respectively. The corresponding exponential coefficients (k₁) are also given.


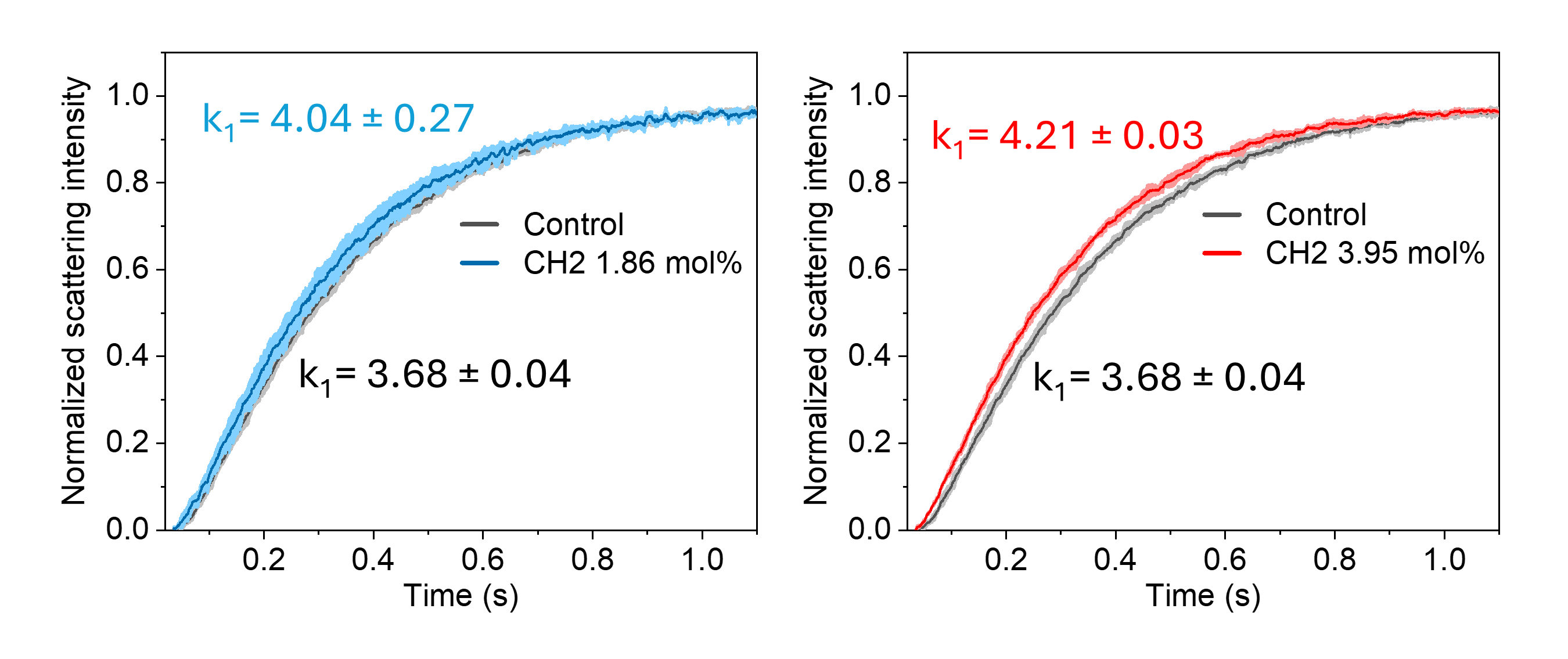


Figure S16. Stopped-flow light-scattering traces of LUVs in the absence and presence of **CH2** at 1.86 mol% and 3.95 mol%, respectively. The corresponding exponential coefficients (k₁) are also given.


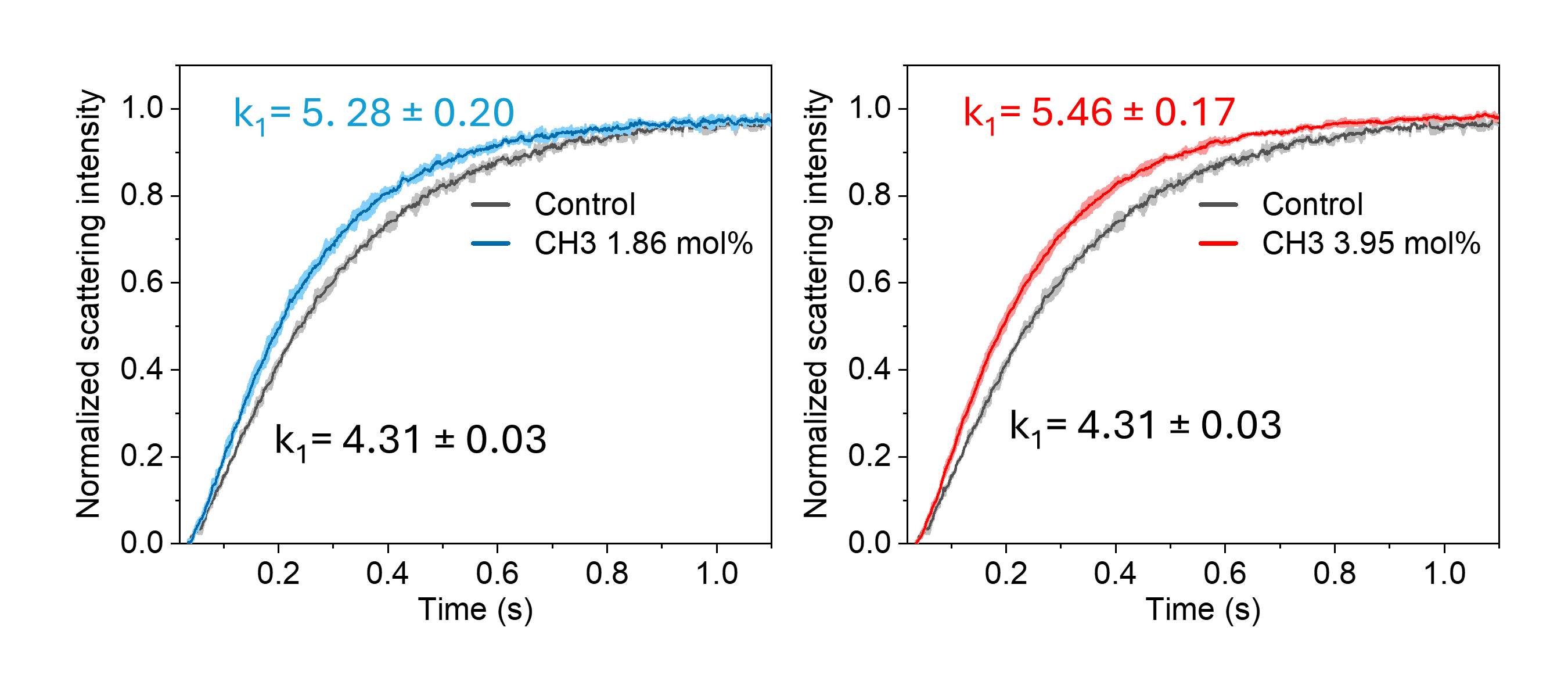


Figure S17. Stopped-flow light-scattering traces of LUVs in the absence and presence of **CH3** at 1.86 mol% and 3.95 mol%, respectively. The corresponding exponential coefficients (k₁) are also given.


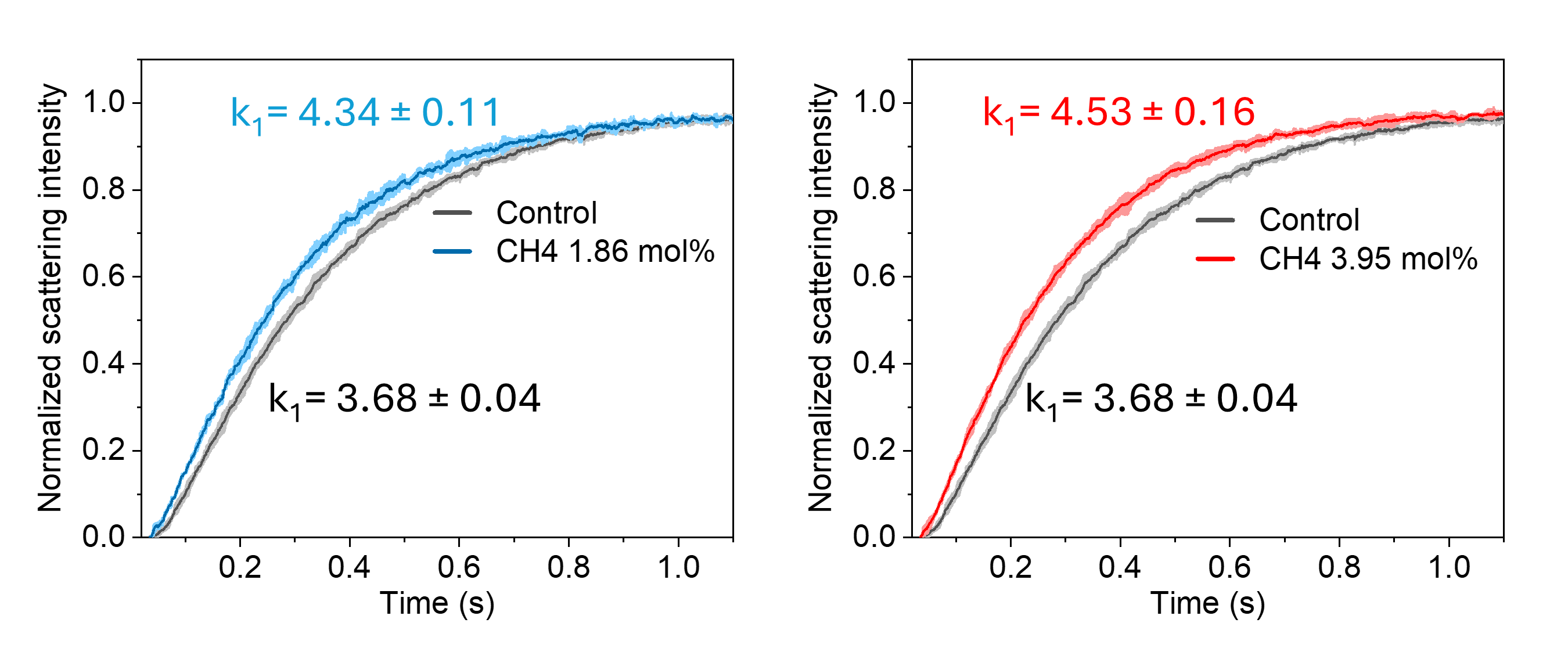


Figure S18. Stopped-flow light-scattering traces of LUVs in the absence and presence of **CH4** at 1.86 mol% and 3.95 mol%, respectively. The corresponding exponential coefficients (k₁) are also given.


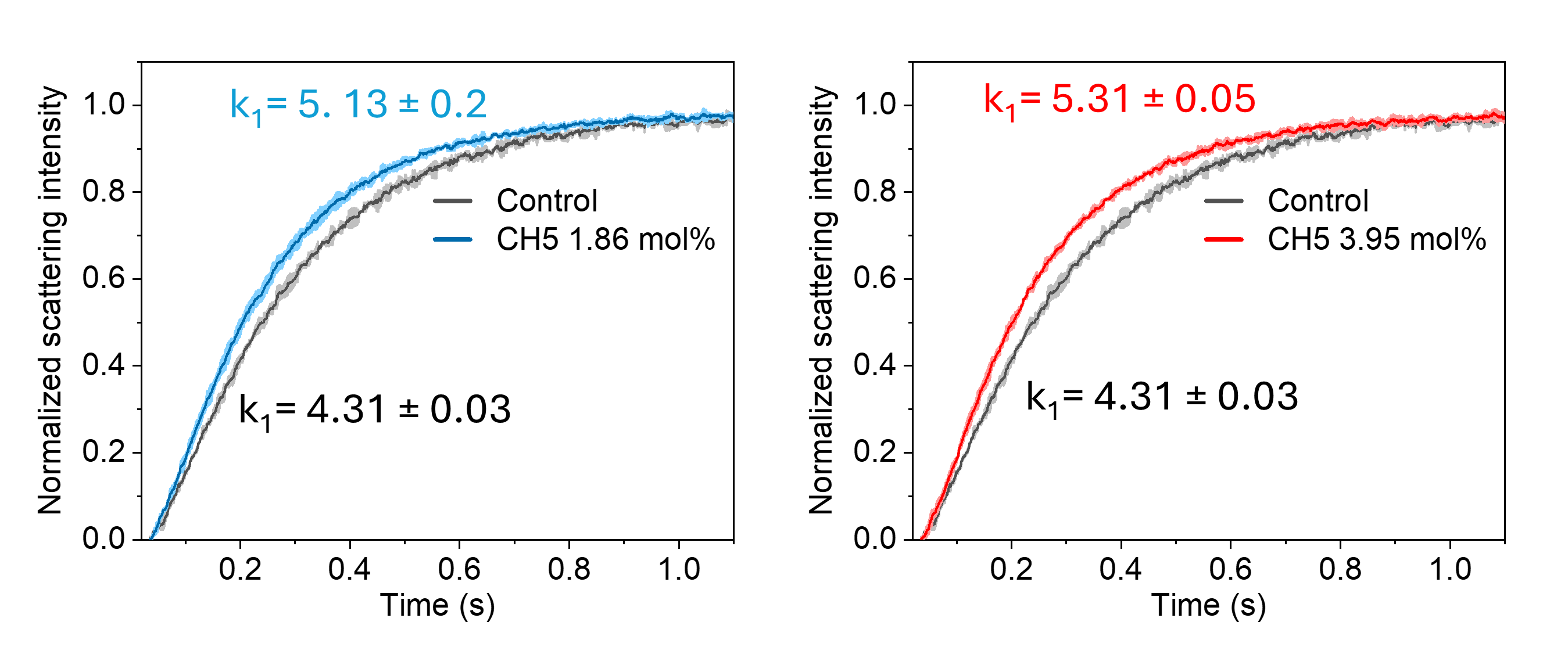


Figure S19. Stopped-flow light-scattering traces of LUVs in the absence and presence of **CH5** at 1.86 mol% and 3.95 mol%, respectively. The corresponding exponential coefficients (k₁) are also given.


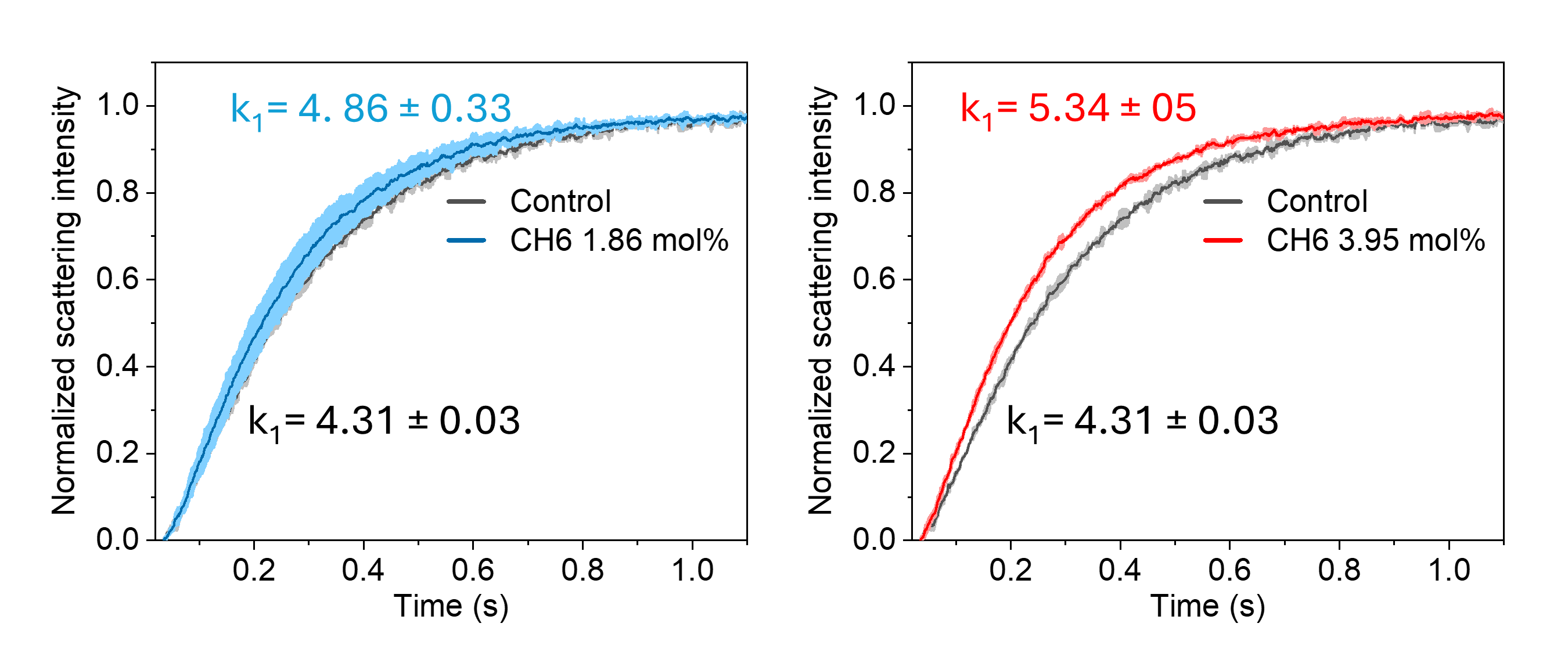


Figure S20. Stopped-flow light-scattering traces of LUVs in the absence and presence of **CH6** at 1.86 mol% and 3.95 mol%, respectively. The corresponding exponential coefficients (k₁) are also given.

# Molecular Dynamics (MD) simulations

The MD simulations were performed using YASARA software.^[5]^ The extracted self-assembly aggregate of **CH4⸧H2O** from the X-ray structure has been placed into POPC lipid bilayer and enclosed in a simulation cell of size 72x63.5x72 Å (Figure S21). The self-assembly aggregate was temporarily scaled by 0.9 along the XZ-axes, and then strongly clashing membrane lipids were deleted (lipids with an atom closer than 0.75 Å to **CH4⸧H2O** aggregate). The temporary scaling, which was needed to avoid the deletion of too many lipids around the protein, was then slowly removed during a short simulation at 298K in vacuo: the aggregate (with all atoms kept fixed) was scaled by 1.02 along the XZ-axes every 200 femtoseconds, while the membrane was allowed to move, but restrained to ideal geometry. The force field was YAMBER3 as implemented in YASARA.^[6,7]^  As soon as the aggregate had reached its original size again, the simulation cell was filled with water, 2% NaCl and the pH was set to 6.4. The main simulation was then run with PME and 8.0 Å cutoff for non-bonded real space forces, a 2.5 fs time-step, constrained hydrogen atoms, and at constant pressure and temperature (NPT ensemble), with thermostat and barostat coupled to time-average values as described previously^[7]^ the pressure values calculated along the X- and Z-axes (i.e. parallel to the membrane plane) were averaged before rescaling the cell, making sure that the membrane's aspect ratio stayed fixed (semi-isotropic approach). One must note that an initial equilibration period of 250 picoseconds has been run to ensure that the membrane can adapt to the newly embedded **CH4⸧H2O** aggregate. During the equilibration time, the membrane was restrained to avoid distortions while the simulation cell adapted to the pressure exerted by the membrane. Additionally, the water molecules were avoided from entering the bilayer during the equilibration period. The source code of this simulation protocol and visualizations of the individual steps can be found at [www.yasara.org/membranemd](http://www.yasara.org/membranemd).


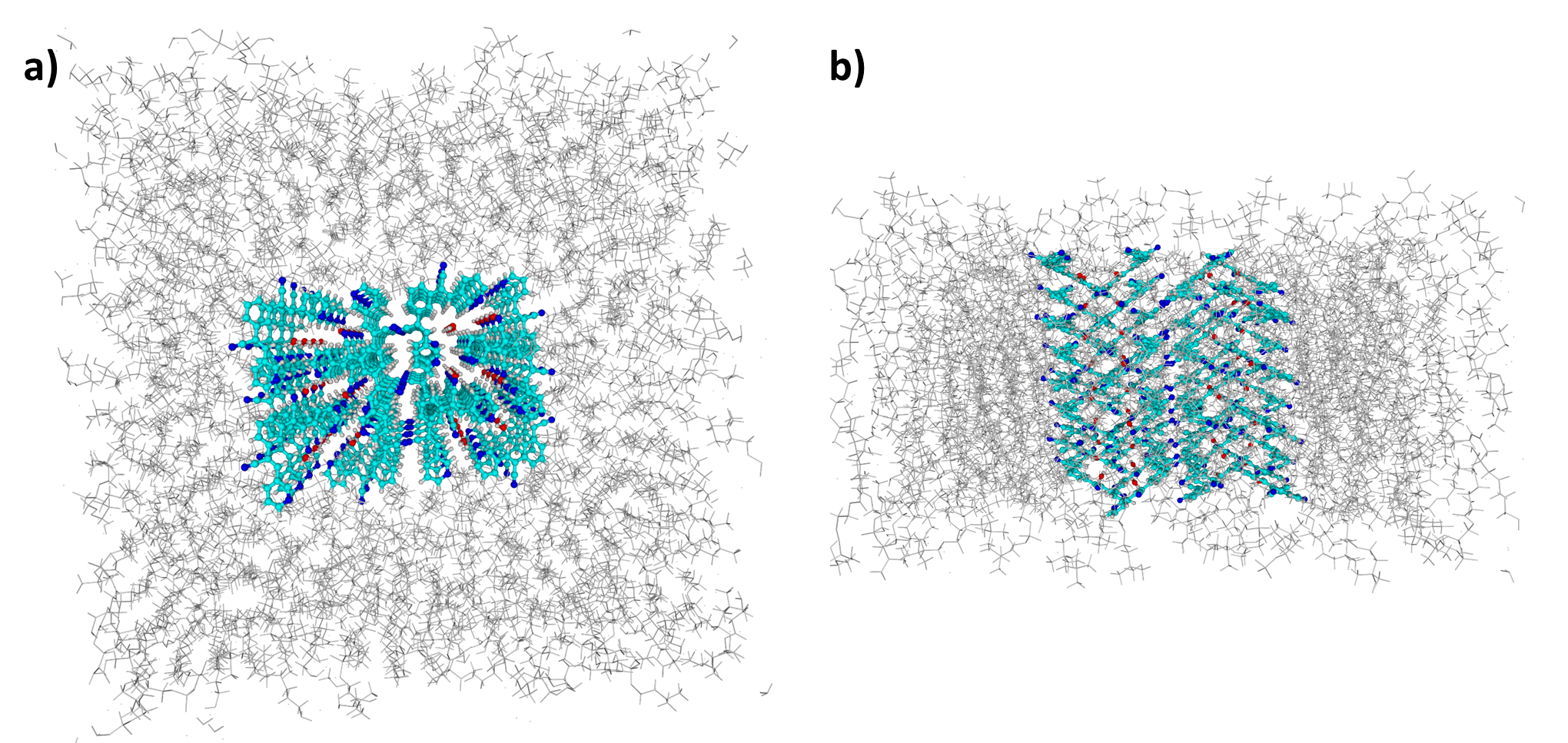


Figure S21. Top view (a) and side view (b) of the selected **CH4@H_2_O** self-assembled aggregate for membrane insertion.


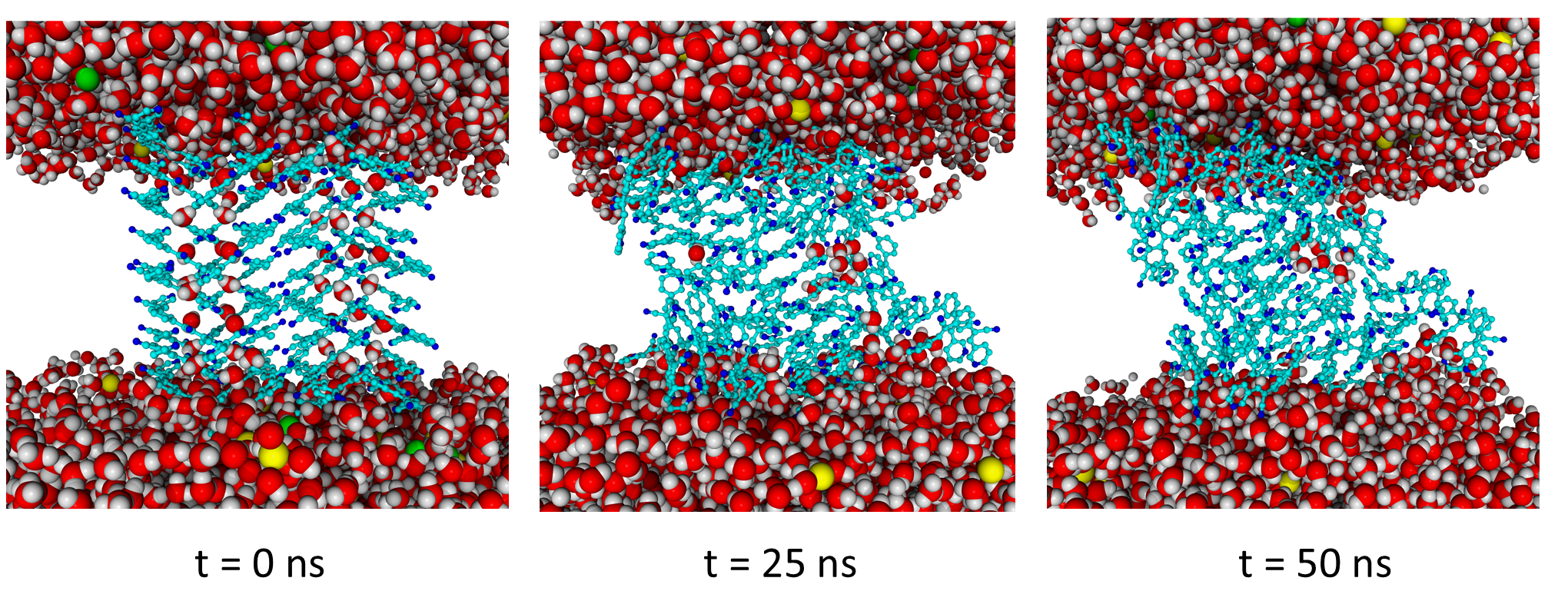


Figure S22. MD simulation snapshots at 0 ns, 25 ns and 50 ns, respectively. Membrane has been omitted for clarity

# Ion Transport tests

**General procedure for the preparation of EYPC large unilamellar vesicles (LUVs)**

Egg yolk L-α-phosphatidylcholine (EYPC, chloroform solution, 2000 μL) was added to a 10 mL round-bottomed flask, the solvent was slowly removed under a flow of argon, and the resulting thin film was further dried in a vacuum desiccator for a minimum of 3 h. The lipid film was hydrated with 1000 μL of different salt solutions (NaX_in_ 100 mM in phosphate buffer 10 mM, pH = 6.4) containing 10 μM HPTS (8-hydroxypyrene-1,3,6-trisulfonic acid trisodium salt) for 1h under periodic gentle vertexing. Afterwards, the suspension was submitted to 10 freeze-thaw cycles (bathed in liquid nitrogen and water at 30 °C, respectively). The large multilamellar liposome suspension (1000 μL) was submitted to extrusion (21 extrusions) at room temperature through a 100 nm polycarbonate membrane to afford a suspension of LUVs with an average diameter of around 100 nm (as confirmed by dynamic light scattering measurements). The LUV suspension was separated from extravesicular dye by size exclusion chromatography (stationary phase: Sephadex G-50, mobile phase: phosphate buffer, 100 mM NaX_in_) and diluted to 7 mL with the same 100 mM NaX_in_ in PBS buffer.

Depending on the transport mechanism under investigation, different assay configurations were employed, as detailed below for each case. The emission of HPTS at 510 nm was monitored with excitation wavelengths at 403 and 460 nm simultaneously, using a Perkin Elmer FL6500 spectrometer. Transport profiles were generated by calculating the ratiometric fluorescence response I_460_/I_403_ and subsequently normalizing the data according to Equation (4).

${y=I}_{f}=100\frac{I_{t}-I_{0}}{I_{1}-I_{0}}$ (4)

where *I_f_* = Fractional emission intensity, *I_t_* = Fractional intensity at time t, *I_1_* = Fluorescence intensity after Triton X-100 addition and *I_0_* = Initial fluorescence intensity.

**Fluorescence measurements to assess the electroneutral transport**

In a quartz fluorometric cuvette, 100 μL LUVs⊃HPTS with NaCl as NaX_in_ salt has been suspended in 1850 µL sodium phosphate (pH = 6.4) with 100 mM MCl, where M = Li^+^, Na^+^, K^+^, and placed in a fluorescence instrument equipped with a magnetic stirrer and thermostat set at 20 °C. The emission of HPTS at 510 nm was monitored with excitation wavelengths at 403 and 460 nm simultaneously, using a Perkin Elmer FL6500 spectrometer. During the experiment, 20 μL of each compound (4.31 mol% in DMSO) was added at t = -40 s (*i.e.*, 40 s before pH gradient creation). At t = 0 s, 27 μL of 0.5 M aqueous NaOH was injected, resulting in a pH gradient of around 1.5. Maximal possible changes in dye emission were obtained at t = 260 s by lysis of the liposomes with detergent (40 μL of 5 % aqueous Triton X100).


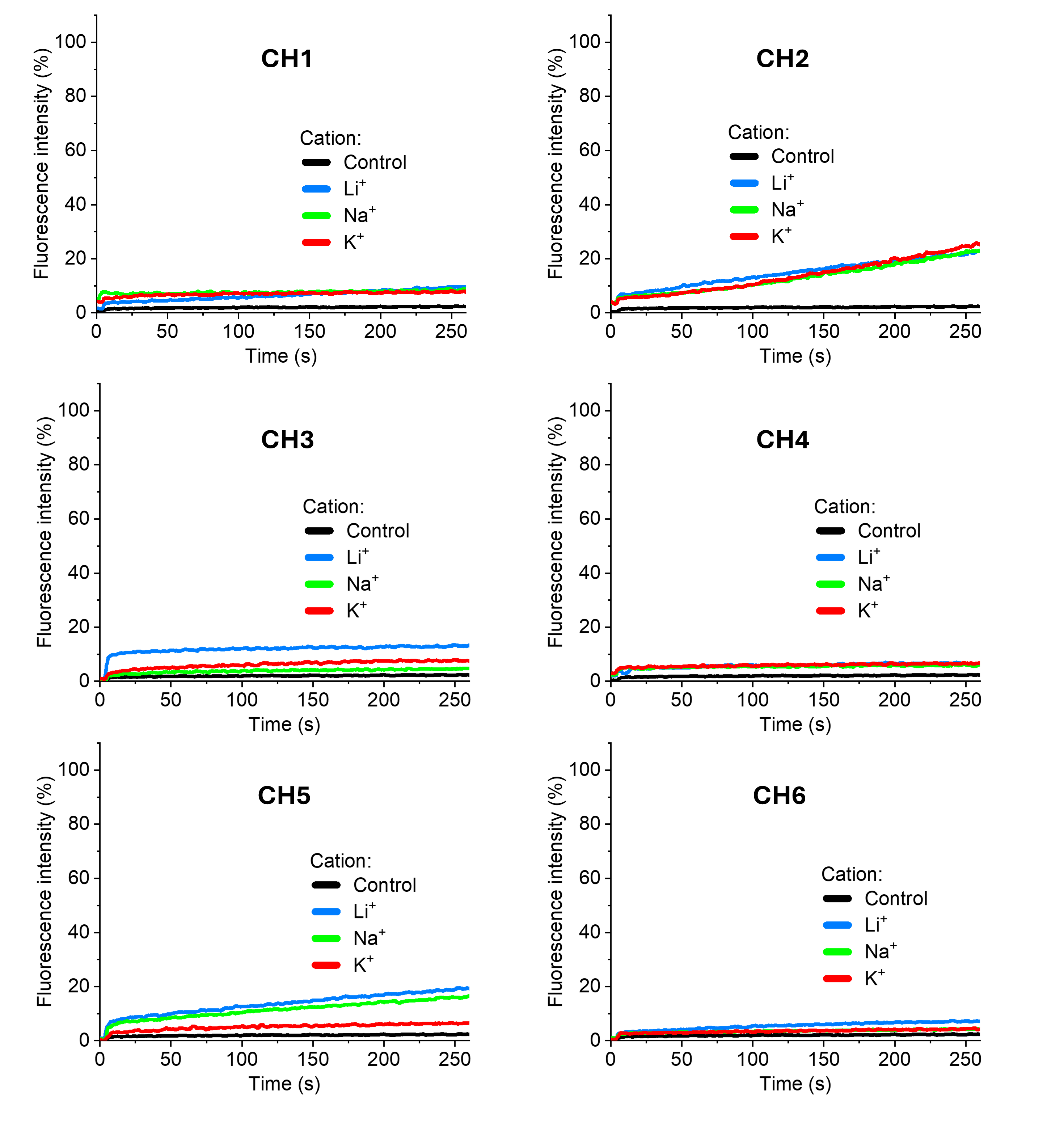


Figure S23. Time-dependent HPTS fluorescence intensity change (%) corresponding to electroneutral H^+^/Cl^-^ symport (1) or the equivalent OH^-^/Cl^-^ antiport (2) and/or M^+^/H^+^ antiport (3) or the equivalent M^+^/OH^-^ symport (4) for all studied **CH** derivatives at a 4.31 mol% loading concentration.

**Fluorescence measurements to quantify the H^+^ or OH^-^ uniport**

In a quartz fluorometric cuvette, 100 μL LUVs⊃HPTS with NaCl as NaX_in_ salt has been suspended in 1830 µL sodium phosphate (pH = 6.4) with 100 mM KCl and placed in a fluorescence instrument equipped with a magnetic stirrer and thermostat set at 20 °C. The emission of HPTS at 510 nm was monitored with excitation wavelengths at 403 and 460 nm simultaneously, using a Perkin Elmer FL6500 spectrometer. During the experiment, 20 μL of each compound (4.31 mol% in DMSO, or from 0 to 4.31 mol% for the dose-dependent tests) was added at t = -40 s (*i.e.*, 40 s before pH gradient creation). At t = -20 s (*i.e.*, 20 s before pH gradient creation), 20 μL of Valinomycin (0.108 mol% in DMSO) has been added, followed by 27 μL of 0.5 M aqueous NaOH at t = 0 s. Maximal possible changes in dye emission were obtained at t = 260 s by lysis of the liposomes with detergent (40 μL of 5 % aqueous Triton X100).


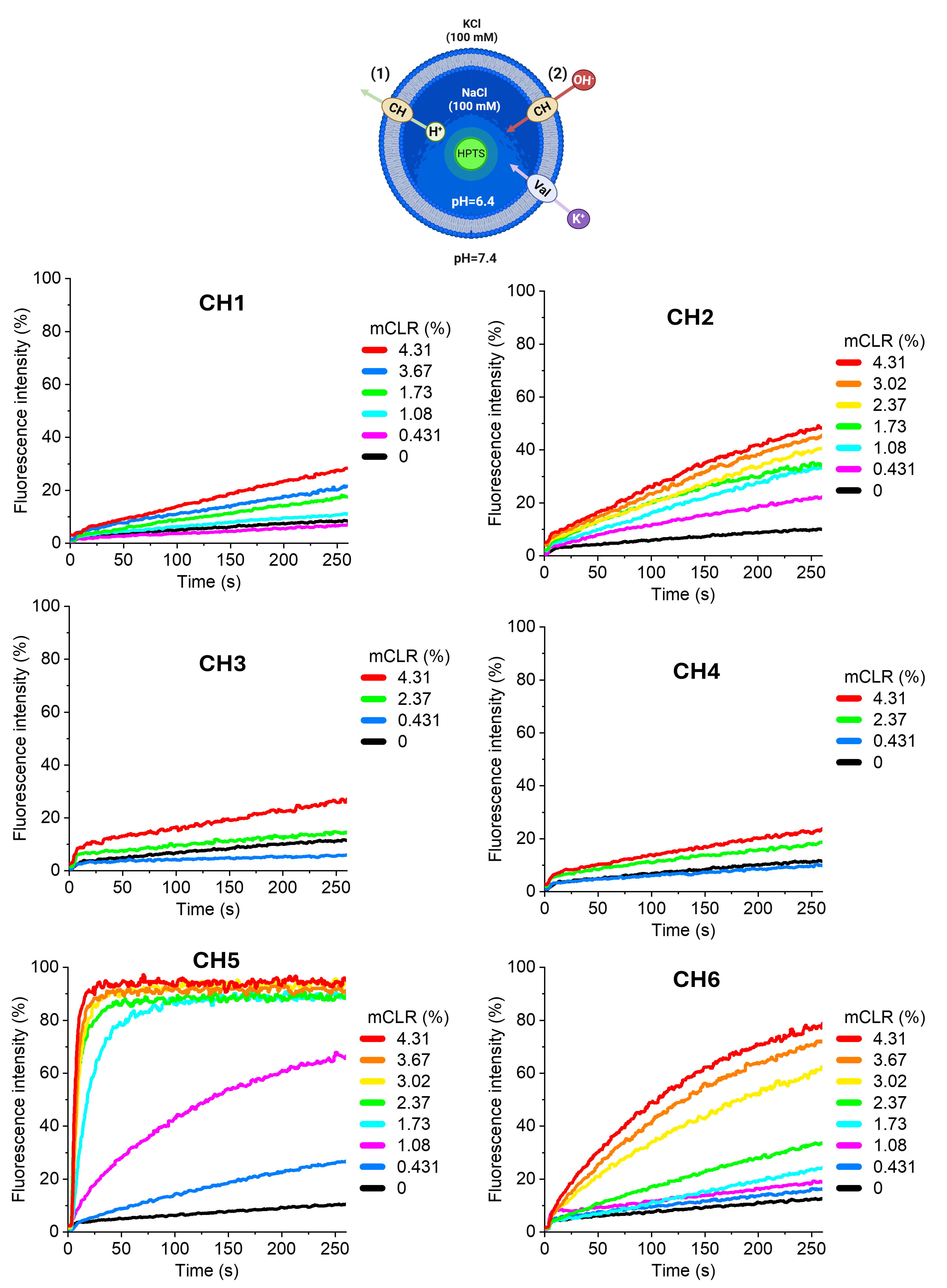


Figure S24. Time-dependent HPTS fluorescence intensity change (%) corresponding to H^+^ uniport (1) or the equivalent OH^-^ uniport (2) in the valinomycin-coupled assay for all studied **CH** derivatives at different loading concentrations (*i.e*., mCLR%).

**Fluorescence measurements to quantify the X^-^ electrogenic transport (uniport)**

In a quartz fluorometric cuvette, 100 μL LUVs⊃HPTS with NaX (X = Cl^-^, Br^-^, NO_3_^-^) as NaX_in_ salt has been suspended in 1830 µL sodium phosphate (pH = 6.4) with 100 mM NaX_out_ (X = Cl^-^, Br^-^, NO_3_^-^) and placed in a fluorescence instrument equipped with a magnetic stirrer and thermostat set at 20 °C. The emission of HPTS at 510 nm was monitored with excitation wavelengths at 403 and 460 nm simultaneously, using a Perkin Elmer FL6500 spectrometer. During the experiment, 20 μL of each compound (from 0 to 4.31 mol% in DMSO) was added at t = -40 s (*i.e.*, 40 s before pH gradient creation). At t = -20 s (*i.e.*, 20 s before pH gradient creation), 20 μL of FCCP (0.108 mol% in DMSO) has been added, followed by 27 μL of 0.5 M aqueous NaOH at t = 0 s. Maximal possible changes in dye emission were obtained at t = 260 s by lysis of the liposomes with detergent (40 μL of 5 % aqueous Triton X100).


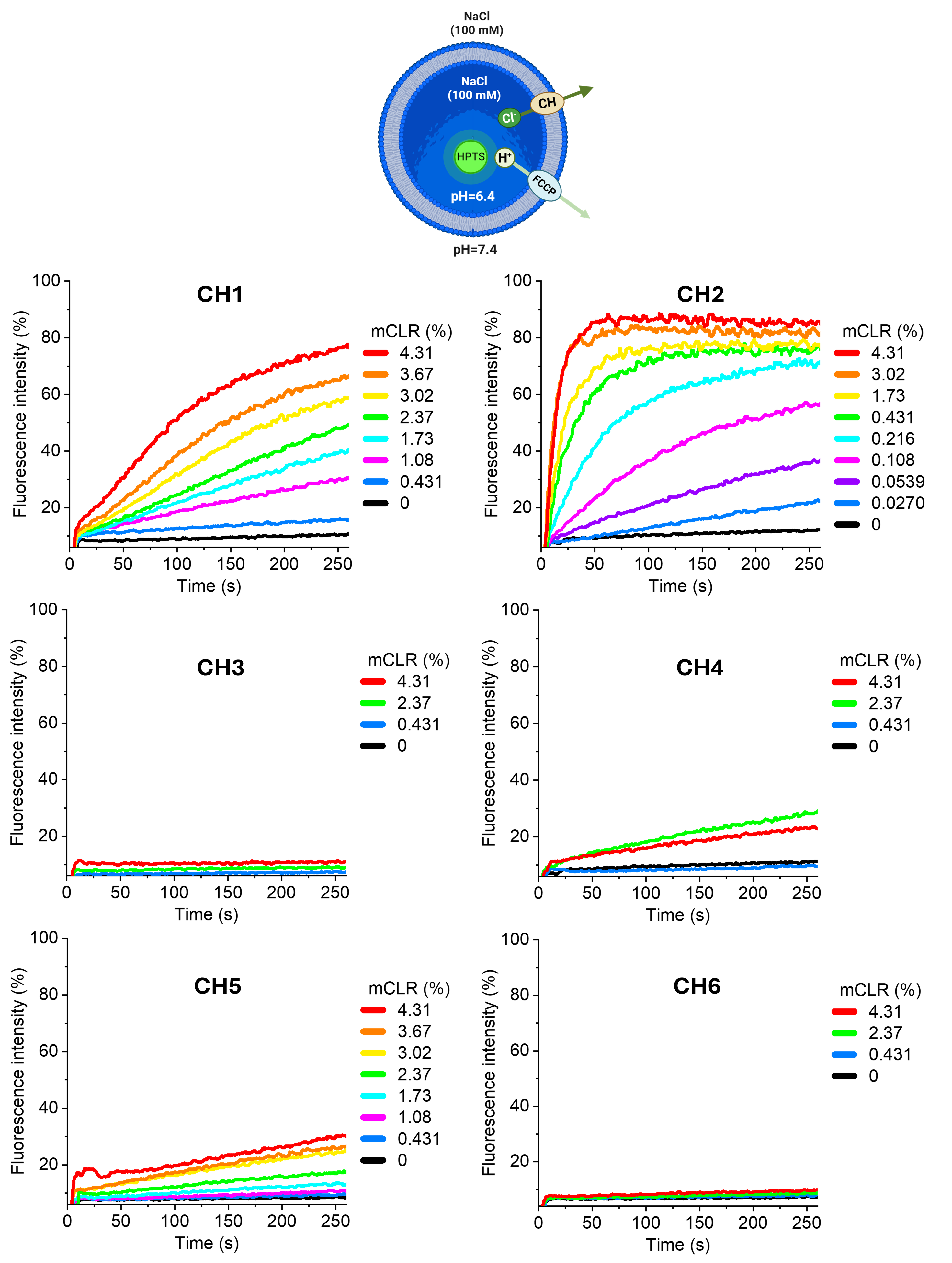


Figure S25. Time-dependent HPTS fluorescence intensity change (%) corresponding to Cl^-^ uniport mediated by the studied **CH** derivatives in the FCCP-coupled assay.


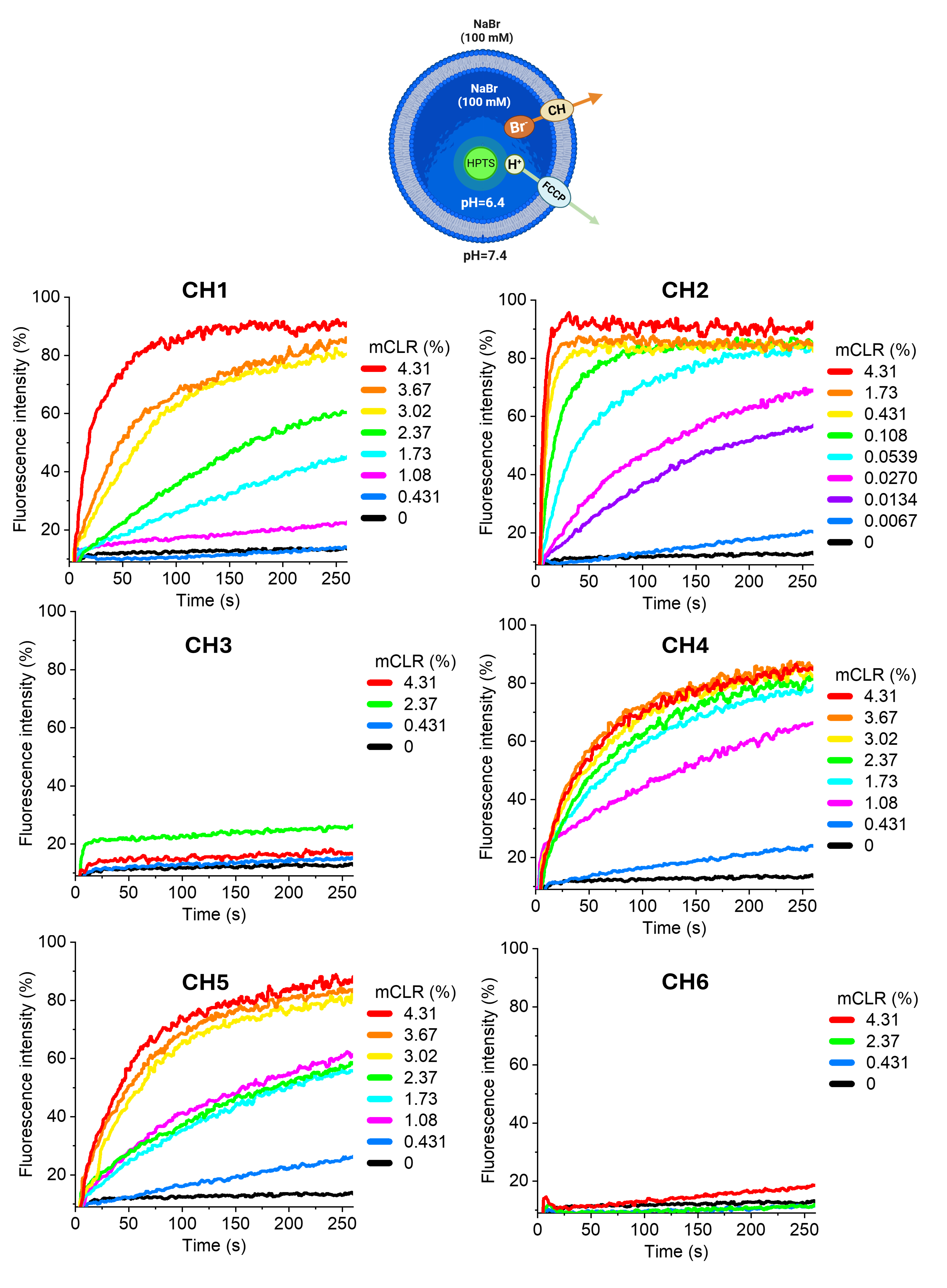


Figure S26. Time-dependent HPTS fluorescence intensity change (%) corresponding to Br^-^ uniport mediated by the studied **CH** derivatives in the FCCP-coupled assay.


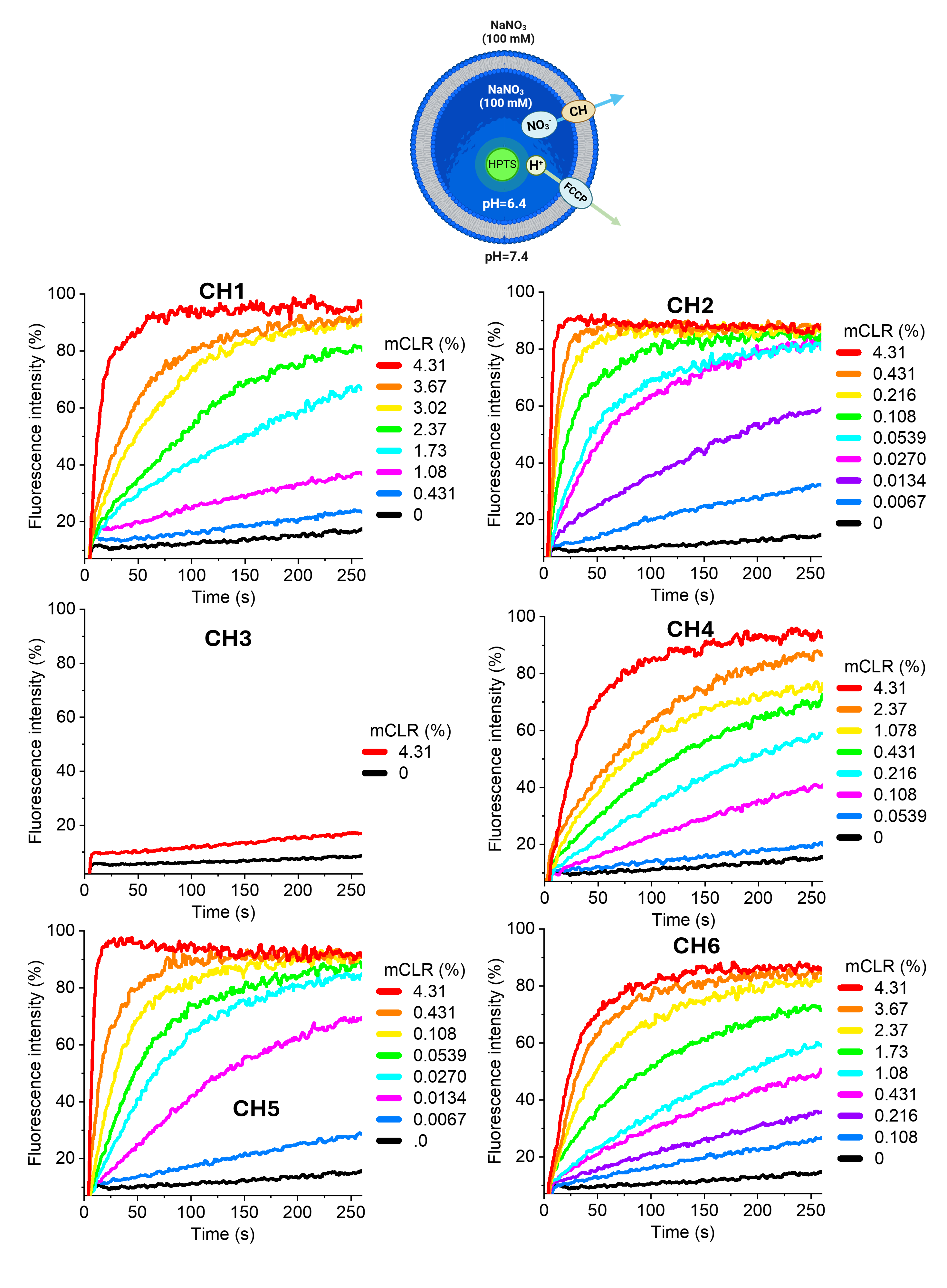


Figure S27. Time-dependent HPTS fluorescence intensity change (%) corresponding to NO_3_^-^ uniport mediated by the studied **CH** derivatives in the FCCP-coupled assay.

**Hill Analysis**

To quantify the anion transport activity of the tested compounds in terms of EC50 (i.e., the concentration required to achieve half-maximal transport), and to determine the degree of cooperativity required for translocation process in terms of Hill number (n), Hill plot analyses were performed by plotting the normalised fluorescence intensity (%) at 250 s as a function of the transporter concentration (mol%). The concentration profile data were fitted using Hill equation (Equation 5) to get k (i.e., *EC_50_*) value and Hill number (n).

$$I_{f}\left( 250 s \right)=y (250 s)=y_{0}+\left( y_{max}-y_{0} \right)\frac{x^{n}}{x^{n}+k^{n}} (5)$$

where y_0_ is the normalized fluorescence intensity of control (no added compound) at t=250 s, y_max_ is the maximum reached fluorescence intensity at t=250 s, x is the concentration (mol%) of the added compound, k is the EC_50_ (mol%), and n is the Hill number.


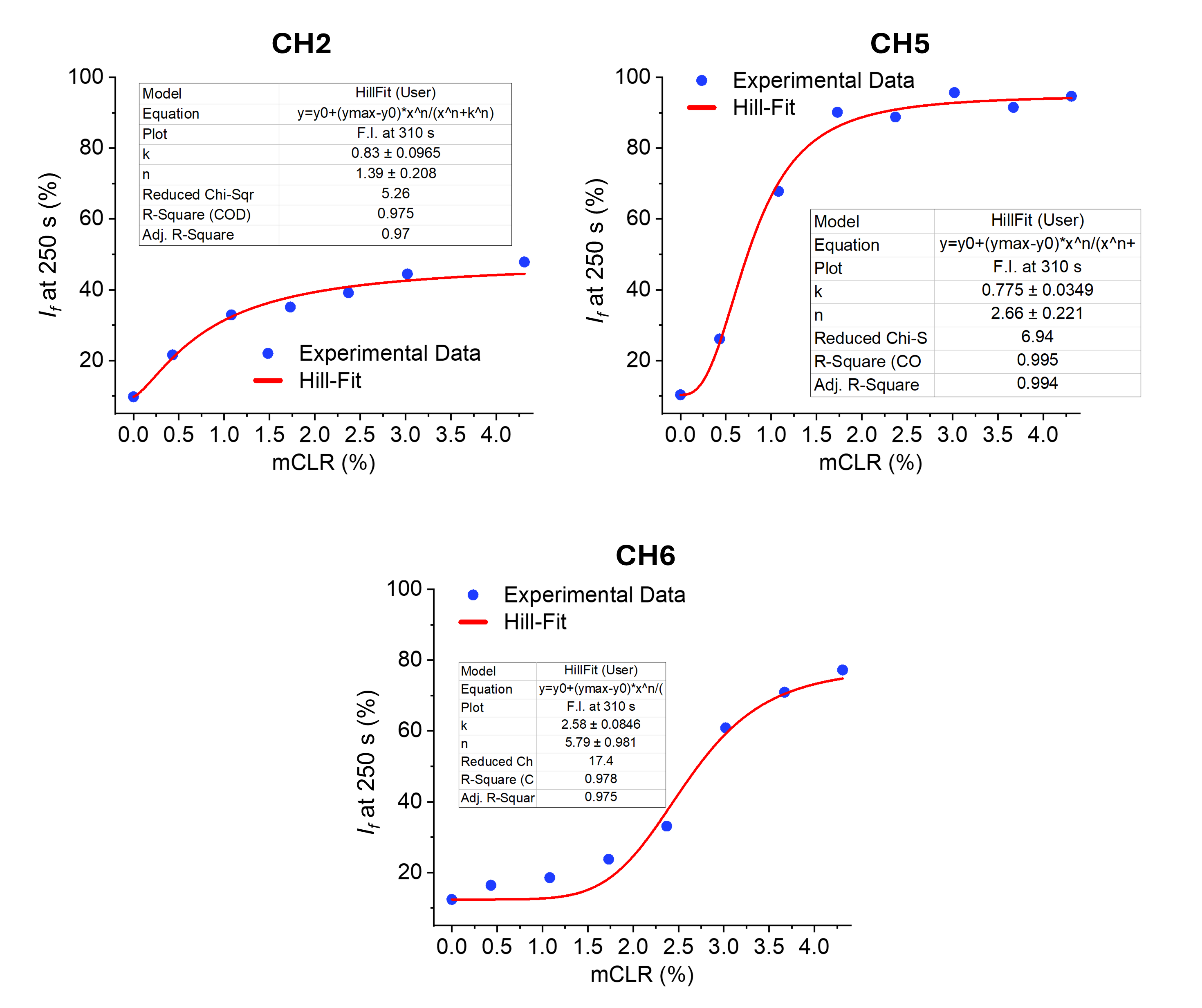


Figure S28. Hill analysis for **CH2, CH5** and **CH6** mediating H^+^ and/or OH^-^ uniport in the Valinomycin-coupled assay.


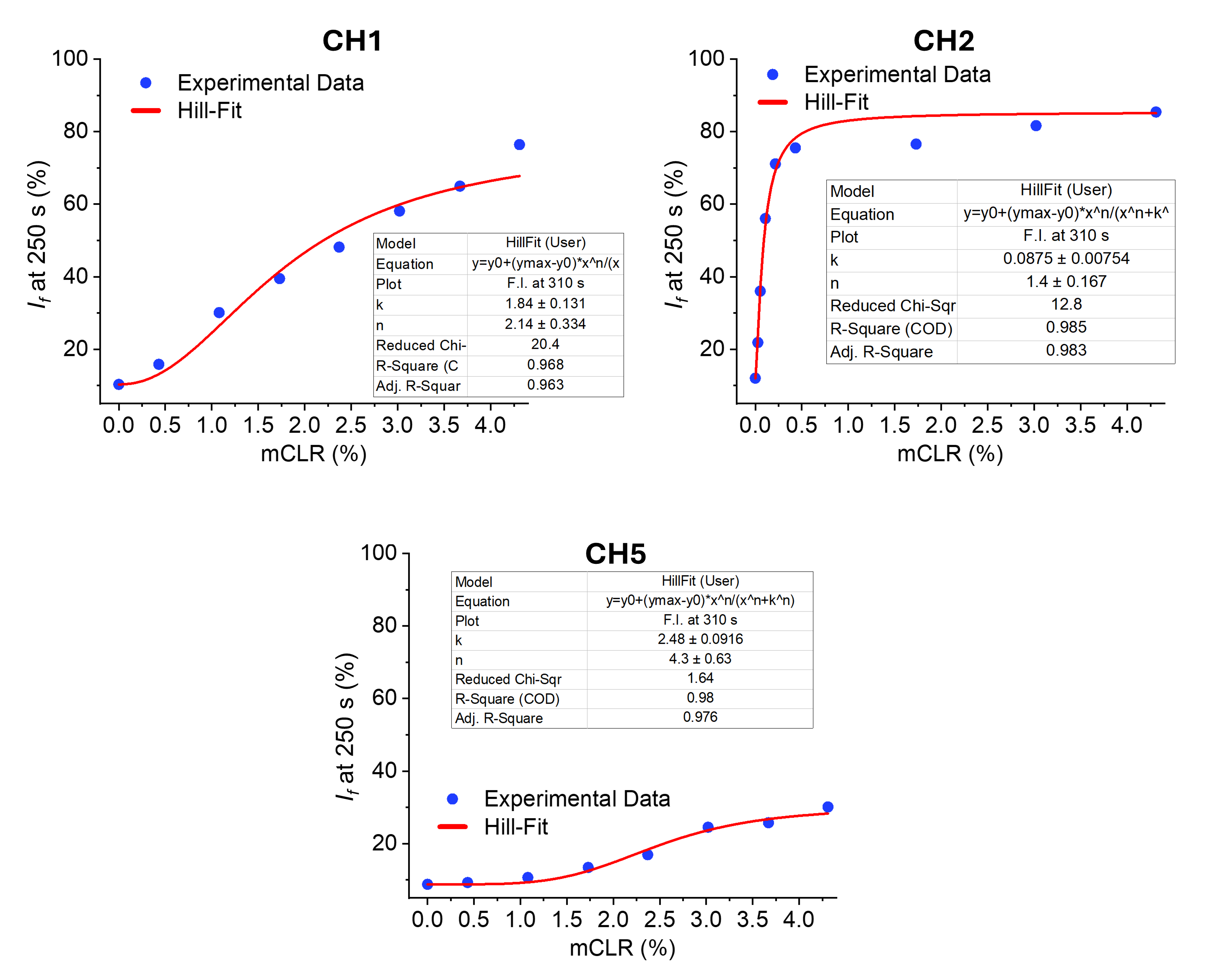


Figure S29. Hill analysis for **CH1, CH2** and **CH5** mediating Cl^−^ uniport.


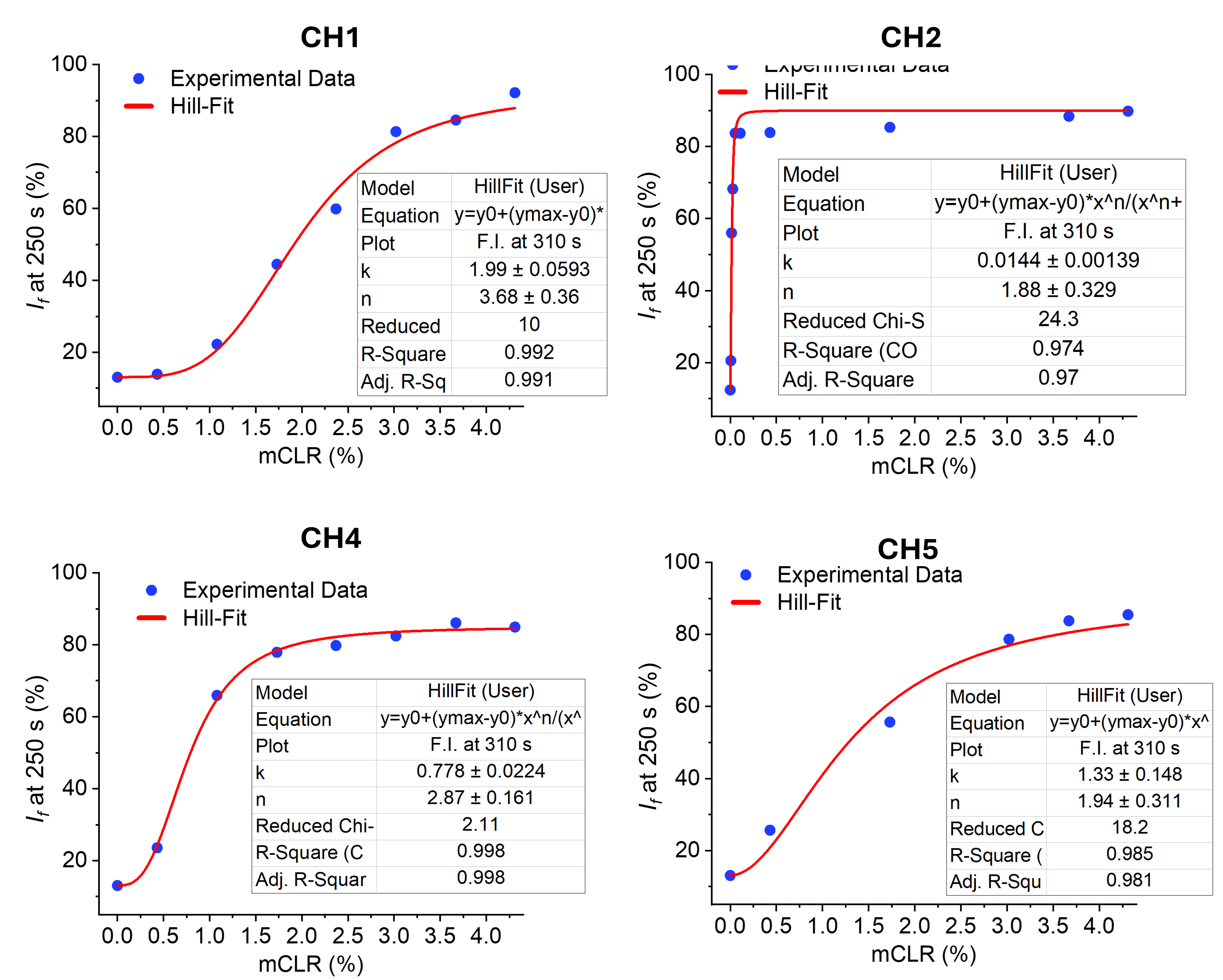


Figure S30. Hill analysis for **CH1, CH2, CH4** and **CH5** mediating Br^−^ uniport.


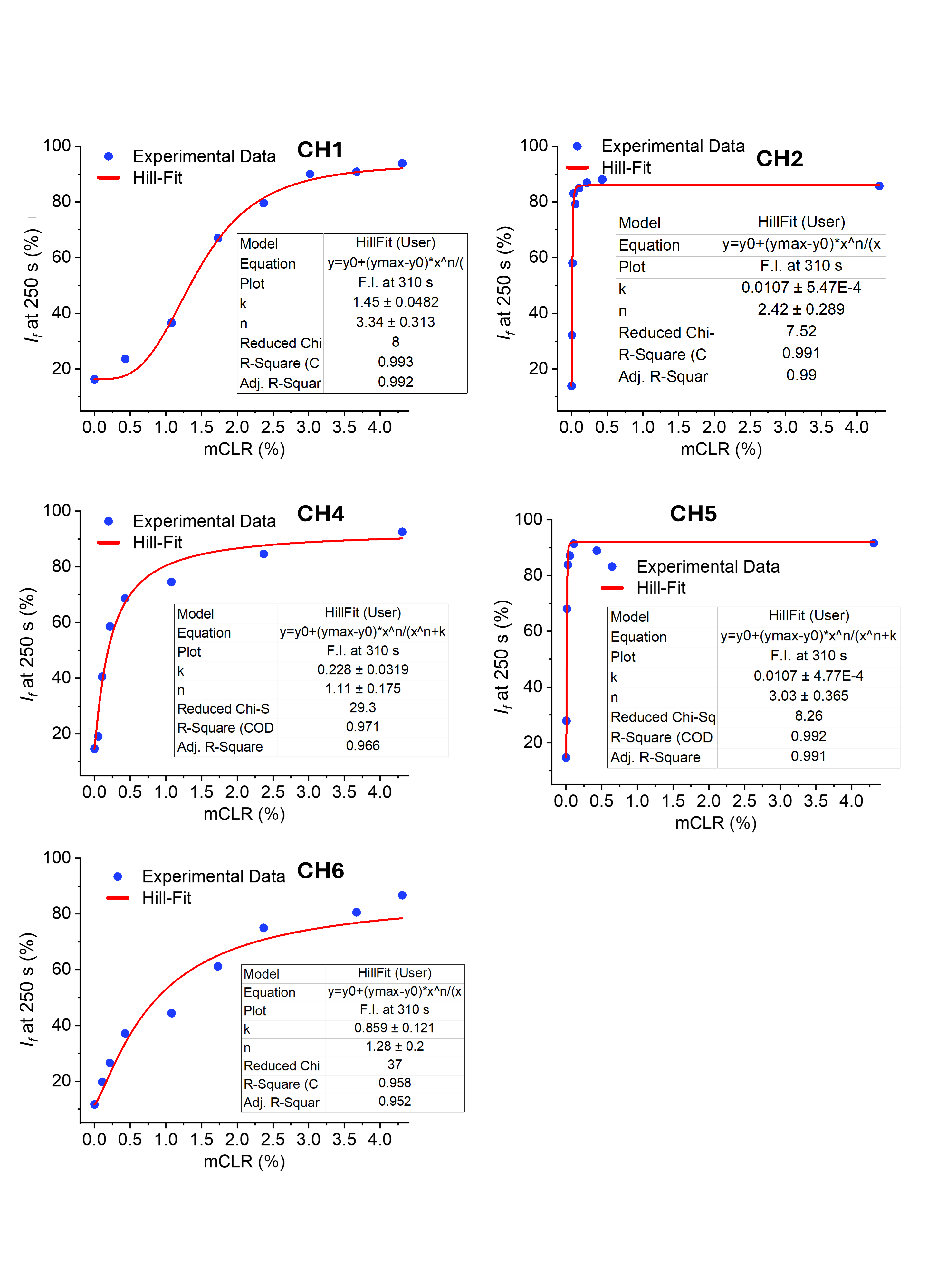


Figure S31. Hill analysis for **CH1, CH2, CH4, CH5** and **CH6** mediating NO_3_^−^ uniport.


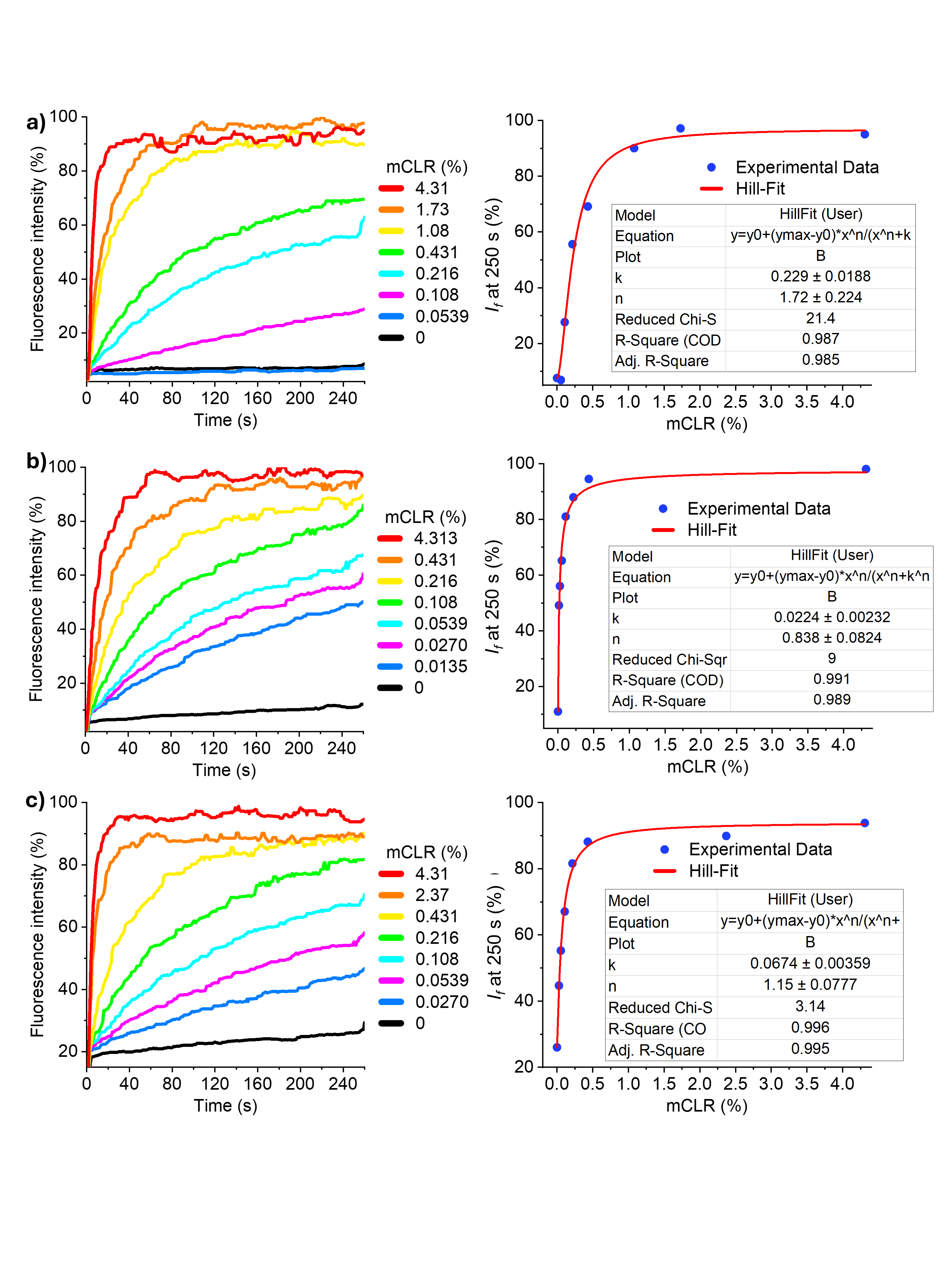


Figure S32. Hill analysis for **NH1** transporter mediating Cl^-^ uniport (a), Br^-^ uniport (b), and NO_3_^-^ uniport (c).

**Initial transport rates**

For the non-linear transport behaviour, to quantify the transport activity in terms of initial transport rates (k_0_), the I*_f_*=y=f(t) curve was fitted using the first-order exponential decay function (6).

$$y=y_{0}+A_{1}e^{-\frac{t}{\alpha}} (6)$$

The initial rate (k_0_) is the first-order derivative of y at t = 0 (7)

$$k_{0}=- \frac{A_{1}}{\alpha} (7)$$

**Transport selectivities**

Anion uniport selectivities were quantified using both EC50 ratios (Equation 8) and fractional ion transport activity ($R_{X^{-}})$ ratios (Equation 9). One should note that both EC50 and fractional ion transport activity ratios were calculated at 250 s (10 s before Triton X-100 addition).

$$S^{EC50} (X_{1}^{-}/X_{2}^{-})=\frac{\frac{1}{{EC}_{50}\left( X_{1}^{-} \right)}}{\frac{1}{{EC}_{50}\left( X_{2}^{-} \right)}} (8)$$

$$S^{R} (X_{1}^{-}/X_{2}^{-})=\frac{R_{X_{1}^{-}}}{R_{X_{2}^{-}}} (9)$$

For each anion, the fractional ion transport activity $R_{X^{-}}$(%) at a given transporter concentration was calculated according to Equation 10.

$$R_{X^{-}}=100\frac{I_{f}\left( Transporter \right)-I_{f}\left( Control \right)}{I_{f}\left( Triton \right)-I_{f}\left( Control \right)} (10)$$

where $I_{f}\left( Transporter \right)$=Fractional emission intensity reached at 250 s by transporter at a given concentration for a specific anion (X^-^), $I_{f}\left( Control \right)$=Fractional emission intensity reached at 250 s by control (no transporter added) for a specific anion (X^-^), $I_{f}\left( Triton \right)$=Fractional emission intensity reached after Triton X-100 addition.

Beside the$\frac{R_{{NO}_{3}^{-}}}{R_{{Cl}^{-}}}$ and $\frac{R_{{NO}_{3}^{-}}}{R_{{Br}^{-}}}$ at 4.31 mol% (Figure 6b in the main text), concentration-dependent $\frac{R_{{NO}_{3}^{-}}}{R_{{Cl}^{-}}}$ selectivities were also calculated Figure S33). As *a priori* expected, the selectivity reaches a maximum near the EC_50_(NO_3_^-^) concentration (see also Figure 6a in the main text) and then decreases as the transporter loading increases. This decline is pronounced for efficient chloride transporters such as **NH1** and **CH2**, whereas it is much less significant for weaker Cl⁻ transporters like **CH1**. In contrast, no decrease in selectivity is observed for the highly selective **CH6** nitrate transporter.


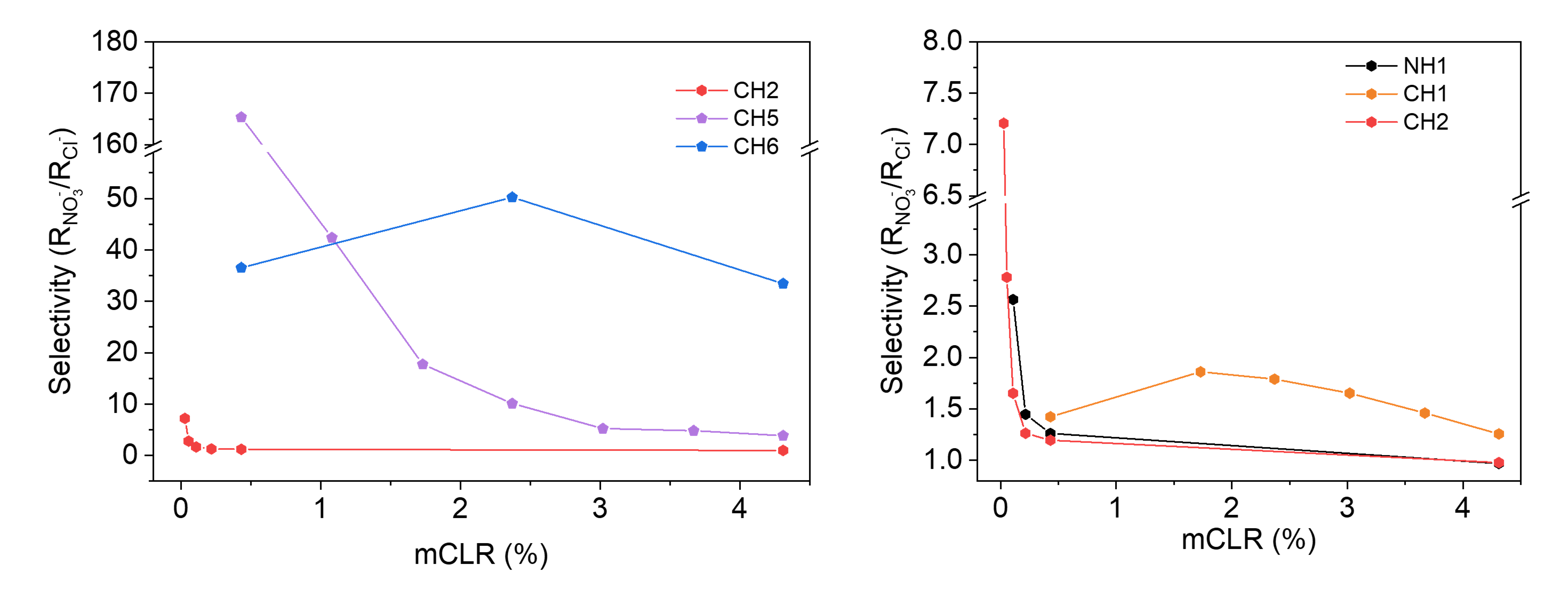


Figure S33. Concentration-dependent $\frac{R_{{NO}_{3}^{-}}}{R_{{Cl}^{-}}}$ selectivities.

**Fluorescence measurements to quantify the X^-^ passive transport**

In a quartz fluorometric cuvette, 100 μL LUVs⊃HPTS with NaX (X = Cl^-^, Br^-^, NO_3_^-^) as NaX_in_ salt has been suspended in 1860 µL sodium phosphate (pH = 6.4) with 100 mM NaGluconate and placed in a fluorescence instrument equipped with a magnetic stirrer and thermostat set at 20 °C. The emission of HPTS at 510 nm was monitored with excitation wavelengths at 403 and 460 nm simultaneously, using a Perkin Elmer FL6500 spectrometer. During the experiment, 20 μL of FCCP (0.108 mol% in DMSO) was added, followed by 20 μL of each compound (4.31 mol% in DMSO) to initiate the transport.


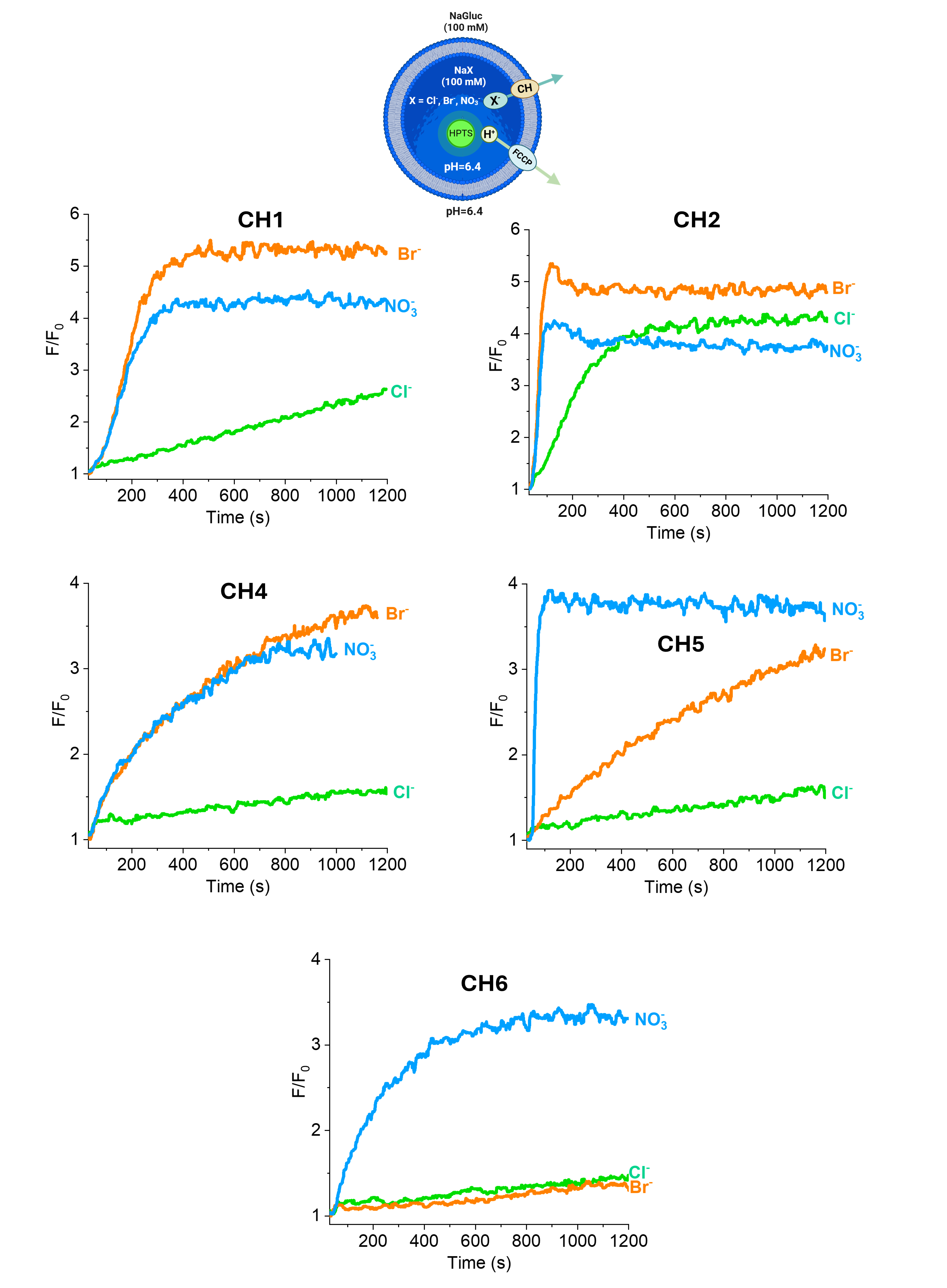


Figure S34. Time-dependent HPTS fluorescence intensity change (F/F_0_) corresponding to anion passive transport in the absence of any pH gradient for CH donors at 4.31 mol%.

**Fluorescence measurements for the competitive anion transport tests**

In a quartz fluorometric cuvette, 100 μL LUVs⊃HPTS with NaNO_3_ as NaX_in_ salt has been suspended in 1880 µL sodium phosphate (pH = 6.4) with 100 mM NaCl, 100 mM NaHCO_3_, or 66.67 mM Na_2_SO_4_ and placed in a fluorescence instrument equipped with a magnetic stirrer and thermostat set at 20 °C. The emission of HPTS at 510 nm was monitored with excitation wavelengths at 403 and 460 nm simultaneously, using a Perkin Elmer FL6500 spectrometer. During the experiment, 20 μL of FCCP (0.108 mol% in DMSO) was added, followed by 20 μL of each compound (4.31 mol% in DMSO) to initiate the transport.


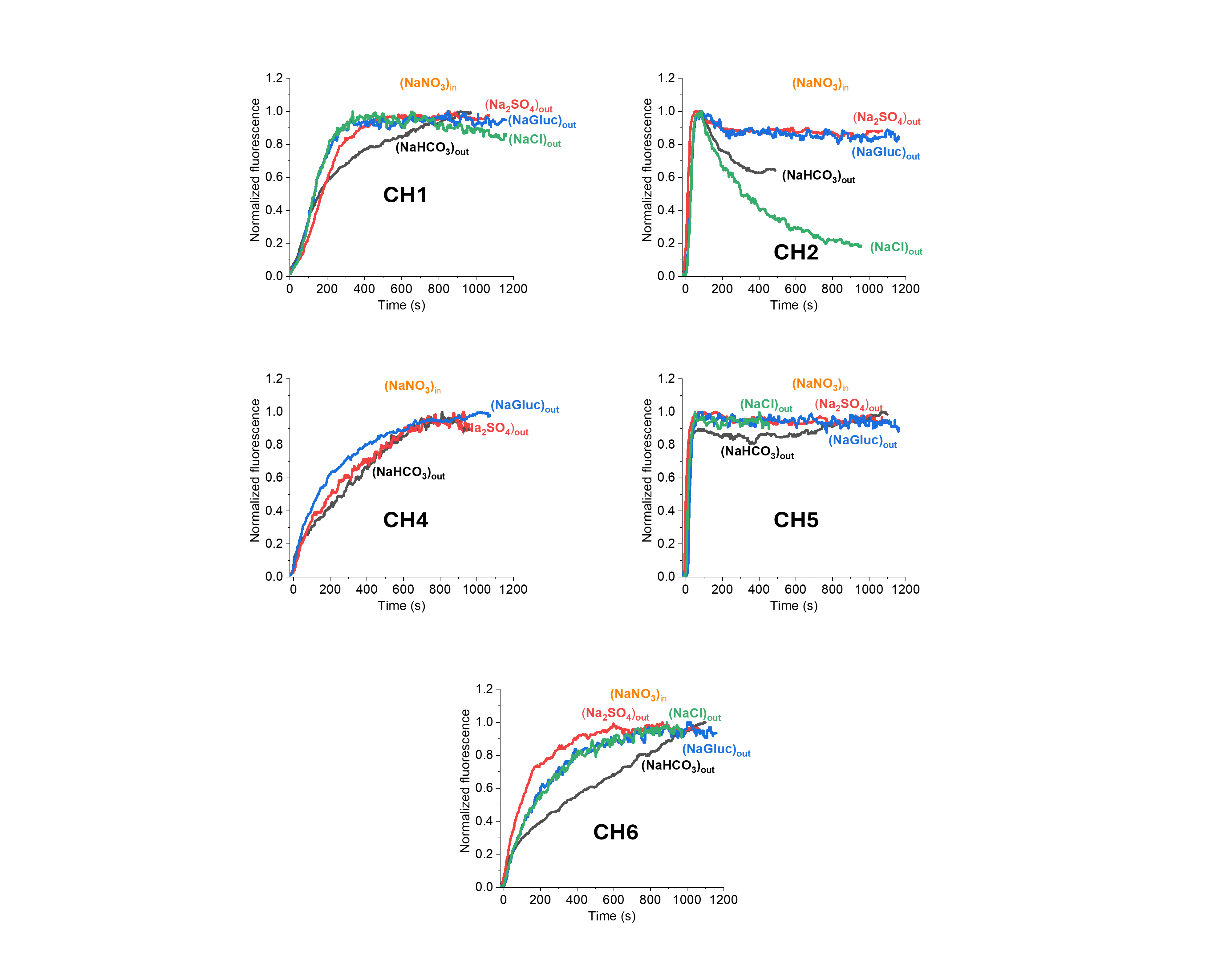


Figure S35. Time-dependent HPTS fluorescence intensity change (F/F_0_) corresponding to NO_3_^-^ efflux (accompanied by H+ efflux through FCCP pathway) in presence of different external anions. An external anion (A^-^) influx (accompanied by H+ influx through FCCP pathway) results in HPTS fluorescence decay proportional to the A^-^ influx. Very slow Cl⁻ influx is observed for **CH1**, while **CH2** translocate Cl- much faster. Significant HCO_3_^-^ transport (influx) is observed only in the case of **CH2**, while SO_4_^2-^ influx is not mediated by any of the studied CH donors.

# Carboxyfluorescein (CF) leakage assay

**LUVs preparation** Egg yolk L-α-phosphatidylcholine (EYPC, chloroform solution, 2000 μL) was added to a 10 mL round-bottomed flask, the solvent was slowly removed under a flow of argon, and the resulting thin film was further dried in a vacuum desiccator for a minimum of 3 h. The lipid film was hydrated with 1000 μL of CF (carboxyfluorescein) 35 mM and 100 mM NaCl buffered in HEPES (10 mM) at pH = 7 for 1h under periodic gentle vertexing. Afterwards, the suspension was submitted to 10 freeze-thaw cycles (bathed in liquid nitrogen and water at 30 °C, respectively). The large multilamellar liposome suspension (1000 μL) was submitted to extrusion (21 extrusions) at room temperature through a 100 nm polycarbonate membrane to afford a suspension of LUVs with an average diameter of around 100 nm (as confirmed by dynamic light scattering measurements). The LUV suspension was separated from extravesicular dye by size exclusion chromatography (stationary phase: Sephadex G-50, mobile phase: HEPES, 100 mM NaCl, pH = 7) and diluted to 7 mL with the same 100 mM NaCl in HEPES buffer.

**Fluorescence measurements for the CF leakage assay** In a quartz fluorometric cuvette, 100 μL LUVs⊃CF with NaCl as internal salt has been suspended in 1880 µL HEPES (pH = 7) with 100 mM NaCl as external salt, placed in a fluorescence instrument equipped with a magnetic stirrer and thermostat set at 20 °C. The emission of carboxyfluorescein at 519 nm was monitored with excitation wavelengths at 470 nm using a Perkin Elmer FL6500 spectrometer. During the experiment, 20 μL of each compound (4.31 mol% in DMSO) was added at t = 20 s. Maximal possible changes in dye emission were obtained at t = 320 s by lysis of the liposomes with detergent (40 μL of 5 % aqueous Triton X100).

Figure S36. Time-dependent CF fluorescence intensity change (%) showing no detectable leakage induced by any of the studied transporters at a compound-to-lipid molar ratio of 4.31 mol%.

# Planar bilayer experiments

The channel openings were recorded by using BC-535A bilayer clamp (Warner Instrument Corp.) The lipid bilayer was made by using 100 μL DOPC (1,2-di-(9Z-octadecenoyl)-sn glycero-3-phosphocholine, solution in chloroform, 25 mg/mL) was evaporated under vacuum for 2 hours. The resulting film is redissolved in decane, further applied to the aperture of chamber to form bilayer membrane with a capacitance value ranging between 50 and 200 pF. The electrodes (Ag/AgCl) were attached and immersed in 1 M KCl solutions which were bridged with trans and cis chambers. Current traces for the formed channels were recorded at different voltages in 1 M symmetrical baths of NaNO_3_ (10 mM HEPES, 10 mM Tris) or in unsymmetrical baths (Trans = NaNO_3_ 1M, 10 mM HEPES, 10 mM Tris, Cis = NaCl 1M, 10 mM HEPES, 10 mM Tris), and the collected data were processed in Origin using a linear regression of the form y = ax + b, where the slope (a) represents the conductance value (γ), unit: pS.

Nitrate/Chloride selectivity (i.e., *P*_NO3_/*P*_Cl_) was determined using unsymmetrical bath solutions (i.e., 1 M NaCl buffer was used in the cis chamber, and the trans chamber was still filled with 1 M NaNO_3_ buffer). The P_NO3_/P_Cl_ selectivity was calculated using the Goldman-Hodgkin-Katz equation: ε_rev_ = RT/F×In(*P*_cis_/*P_t_*_rans_), where R = universal gas constant (8.314 J·K^-1^·mol^-1^), T = 300 K, F = Faraday's constant (96485 C·mol^-1^), and *P* is the ion permeability in the cis and trans chamber, respectively. The reversal potential (ε_rev_) was determined by fitting the current–voltage (I–V) data using a linear (y=ax+b) where -intercept/slope (that is, -b/a) is the reverse potential value (ε_rev_).


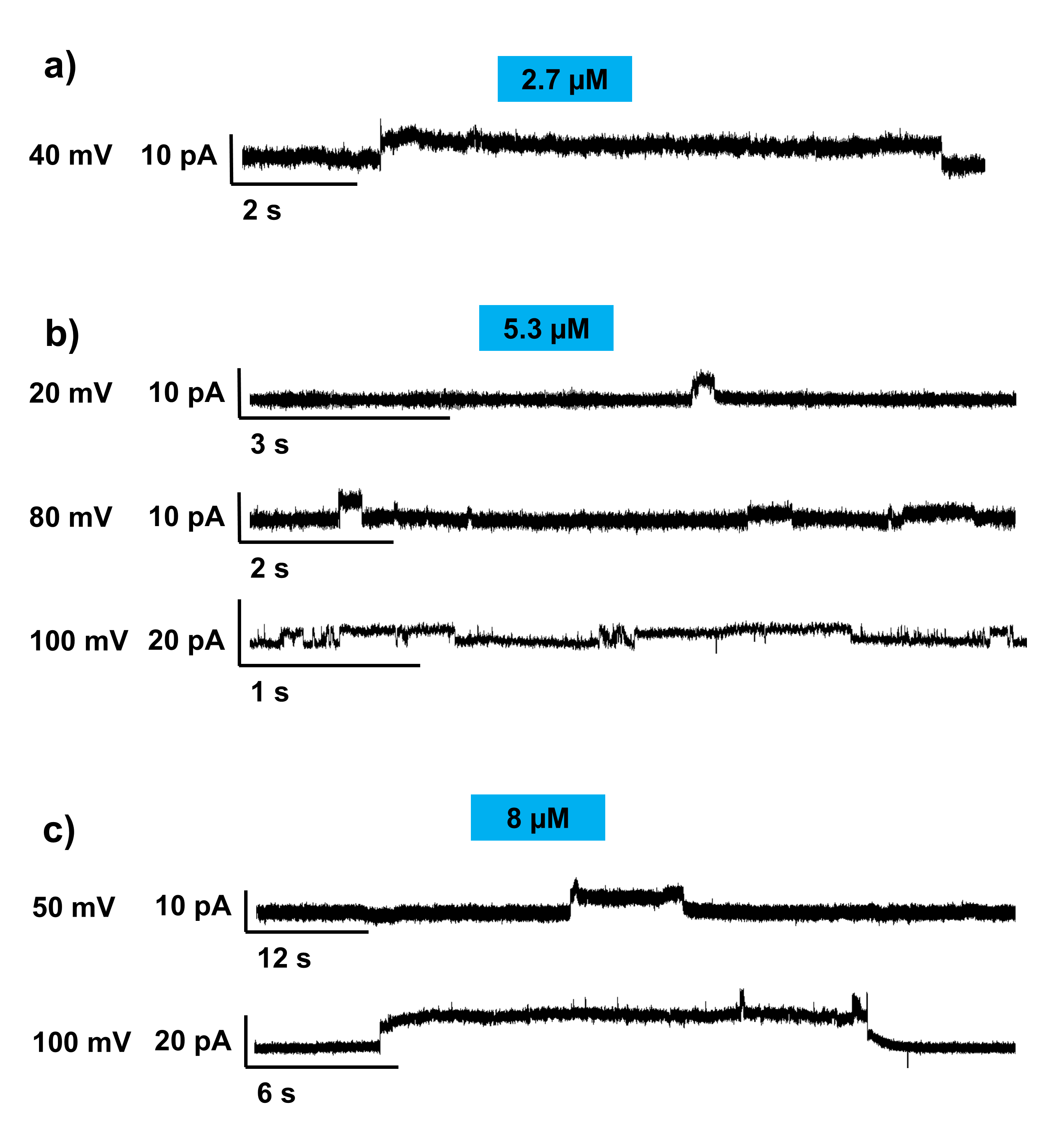


Figure S37. Single channel current of **CH5** at 2.7, 5.3 and 8 µM recorded in symmetrical baths (*trans* = *cis* = 1 M NaNO_3_).


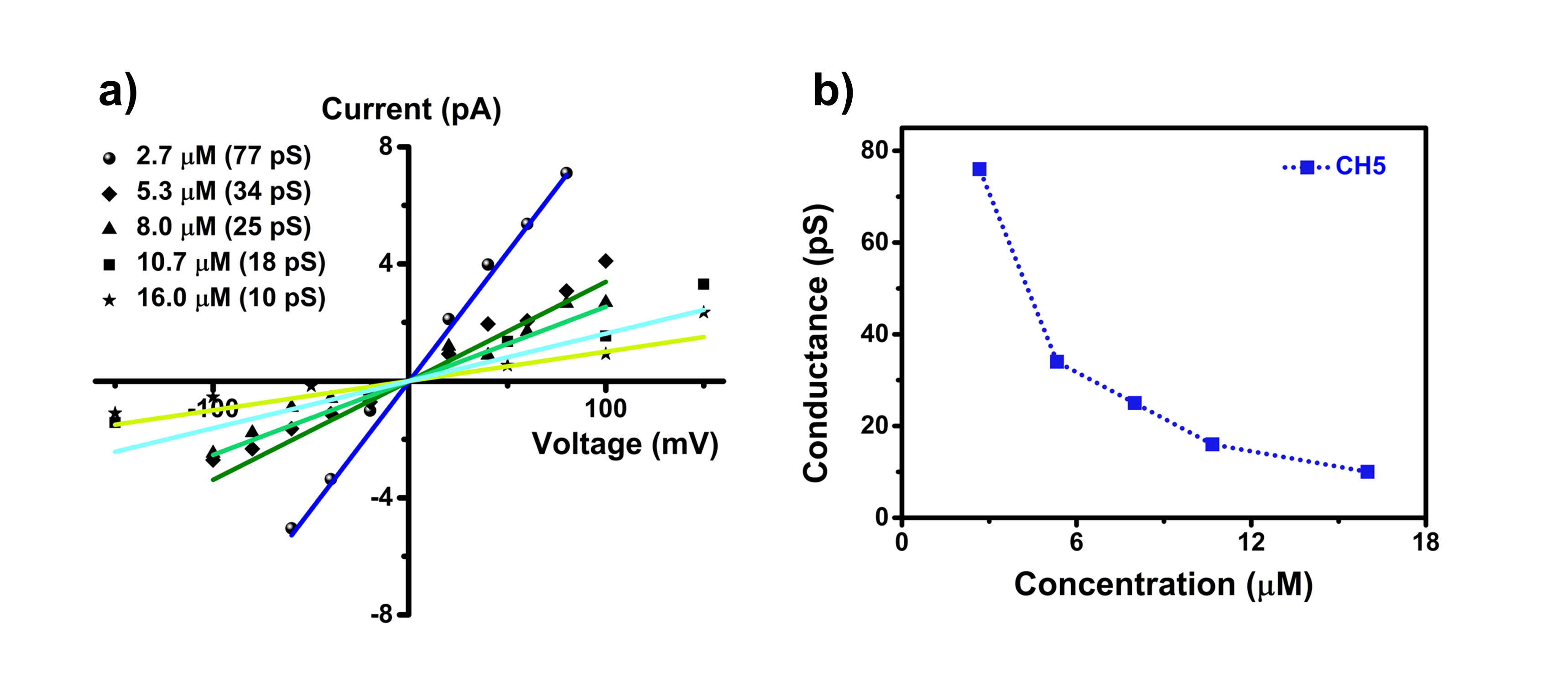


Figure S38. (a) I-V plots of **CH5** at different concentrations in symmetrical baths (*trans* = *cis* = 1 M NaNO_3_.). (b) Nitrate conductance of **CH5** at different concentrations.


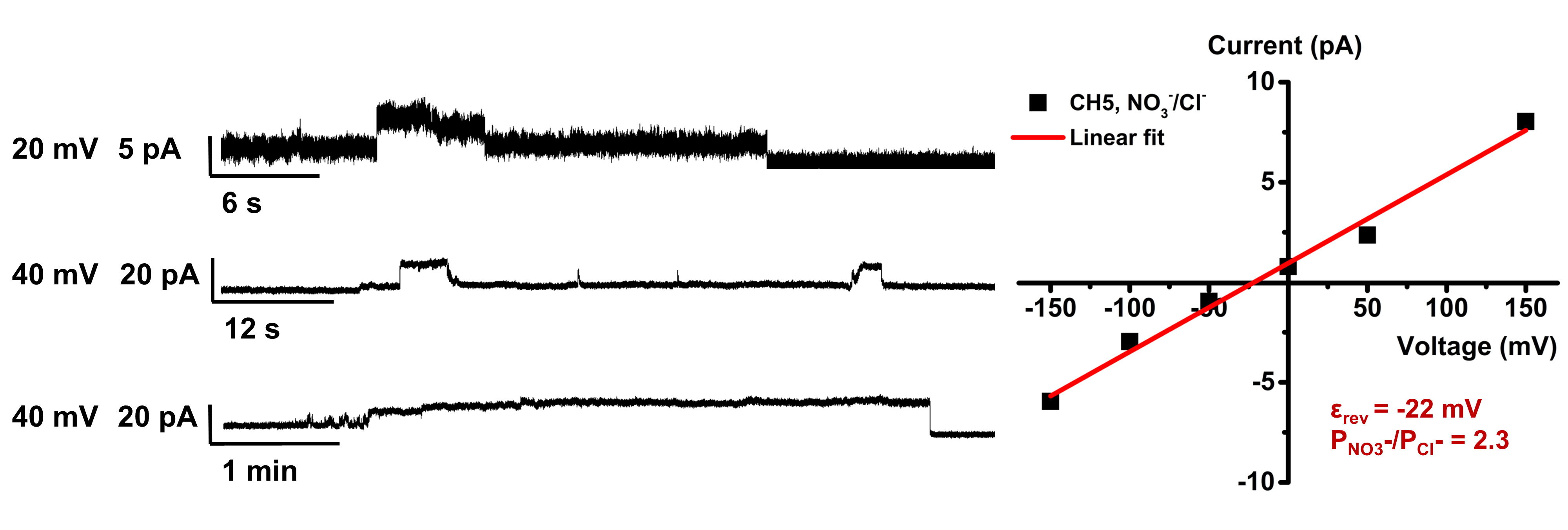


Figure S39. Single channel current and I-V plots of **CH5** recorded in unsymmetrical baths (*trans* = 1 M NaNO3, *cis* = 1 M NaCl).


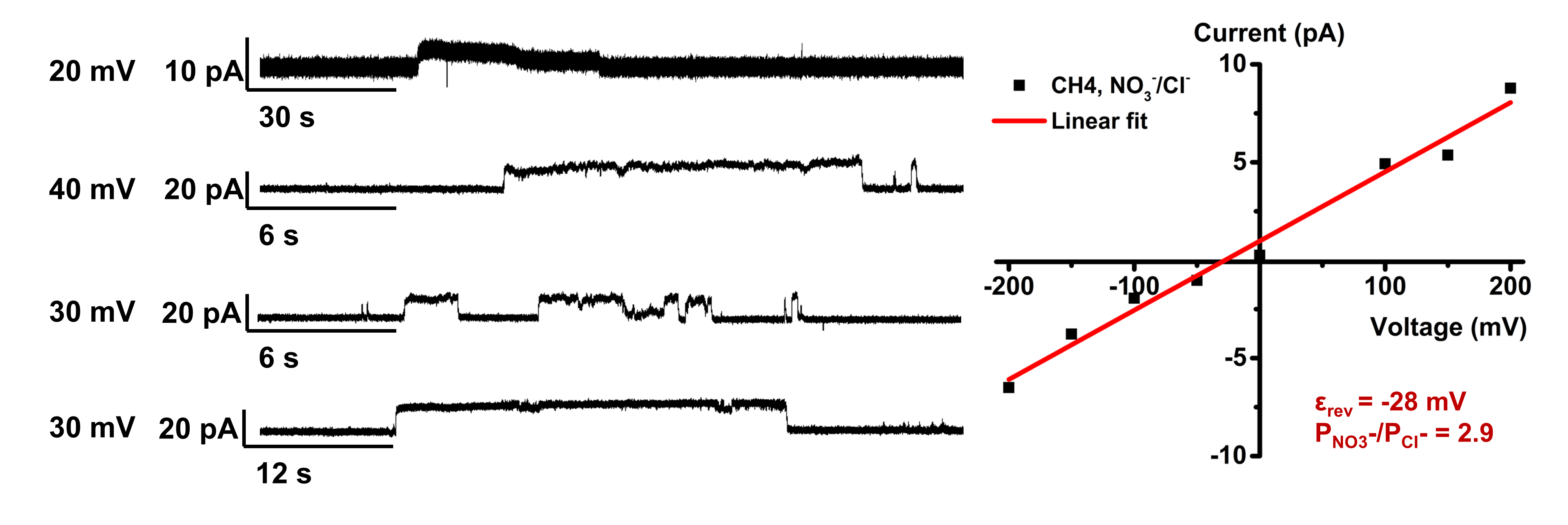


Figure S40. Single channel current and I-V plots of **CH4** recorded in unsymmetrical baths (*trans* = 1 M NaNO3, *cis* = 1 M NaCl).


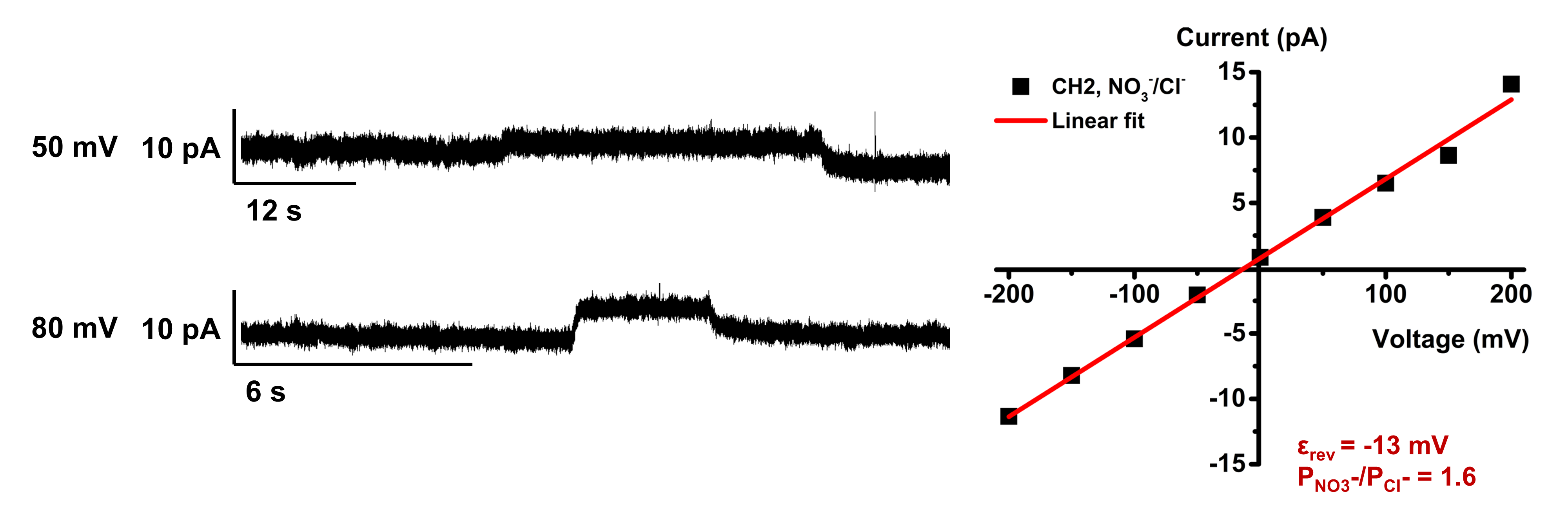


Figure S41. Single channel current and I-V plots of **CH2** recorded in unsymmetrical baths (*trans* = 1 M NaNO3, *cis* = 1 M NaCl).


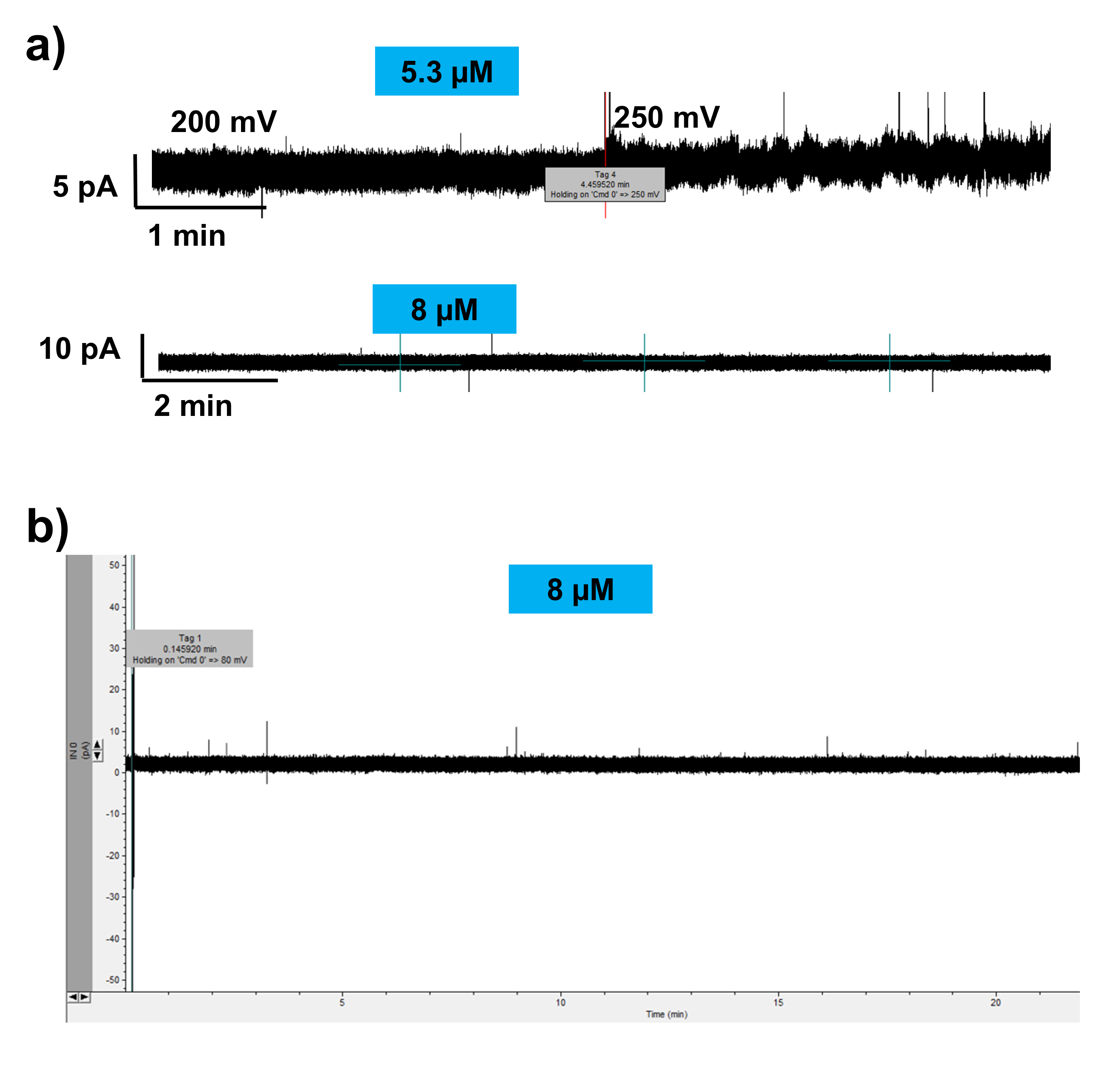


Figure S42. Current scanning of (a) **CH6** and (b) **CH1** in symmetrical baths (trans = cis = 1 M NaNO3).

# DPPC experiments

**General procedure for the preparation of DPPC large unilamellar vesicles (LUVs)**

Dipalmitoylphosphatidylcholine (DPPC, chloroform solution, 2000 μL) was added to a 10 mL round-bottomed flask, the solvent was slowly removed under a flow of argon, and the resulting thin film was further dried in a vacuum desiccator for a minimum of 3 h. The lipid film was hydrated with 1000 μL of NaNO_3_ (100 mM in phosphate buffer 10 mM, pH = 6.4) containing 10 μM HPTS (pyranine, 8-hydroxypyrene-1,3,6-trisulfonic acid trisodium salt) for 1h at 60 °C with gentle vortexing from time to time. Afterwards, the suspension was submitted to 10 freeze-thaw cycles (bathed in liquid nitrogen and water at 60 °C, respectively). The large multilamellar liposome suspension (1000 μL) was submitted to extrusion (21 extrusions) at 60 °C through a 100 nm polycarbonate membrane to afford a suspension of LUVs with an average diameter of around 100 nm. The LUV suspension was separated from extravesicular dye by size exclusion chromatography (stationary phase: Sephadex G-50, mobile phase: phosphate buffer, 100 mM NaNO_3_r) at 50 °C and diluted to 7 mL with the same 100 mM NaNO_3_ in PBS buffer.

**Fluorescence measurements**

In a quartz fluorometric cuvette, 100 μL of the above prepared DPPC⊃HPTS containing NaNO_3_ salt (100 mM) has been suspended in 1830 µL sodium phosphate (pH = 6.4) with 100 mM NaNO_3_ and placed in a fluorescence instrument equipped with a magnetic stirrer and thermostat set at 35 °C or 45 °C. The emission of HPTS at 510 nm was monitored with excitation wavelengths at 403 and 460 nm simultaneously, using a Perkin Elmer FL6500 spectrometer. During the experiment, 20 μL of each compound (4.31 mol% relative to lipids, in DMSO) was added at t = -40 s (*i.e.*, 40 s before pH gradient creation). At t = -20 s (*i.e.*, 20 s before pH gradient creation), 20 μL of FCCP (0.108 mol% in DMSO) has been added, followed by 27 μL of 0.5 M aqueous NaOH at t = 0 s. Maximal possible changes in dye emission were obtained at t = 260 s by lysis of the liposomes with detergent (40 μL of 5 % aqueous Triton X100).


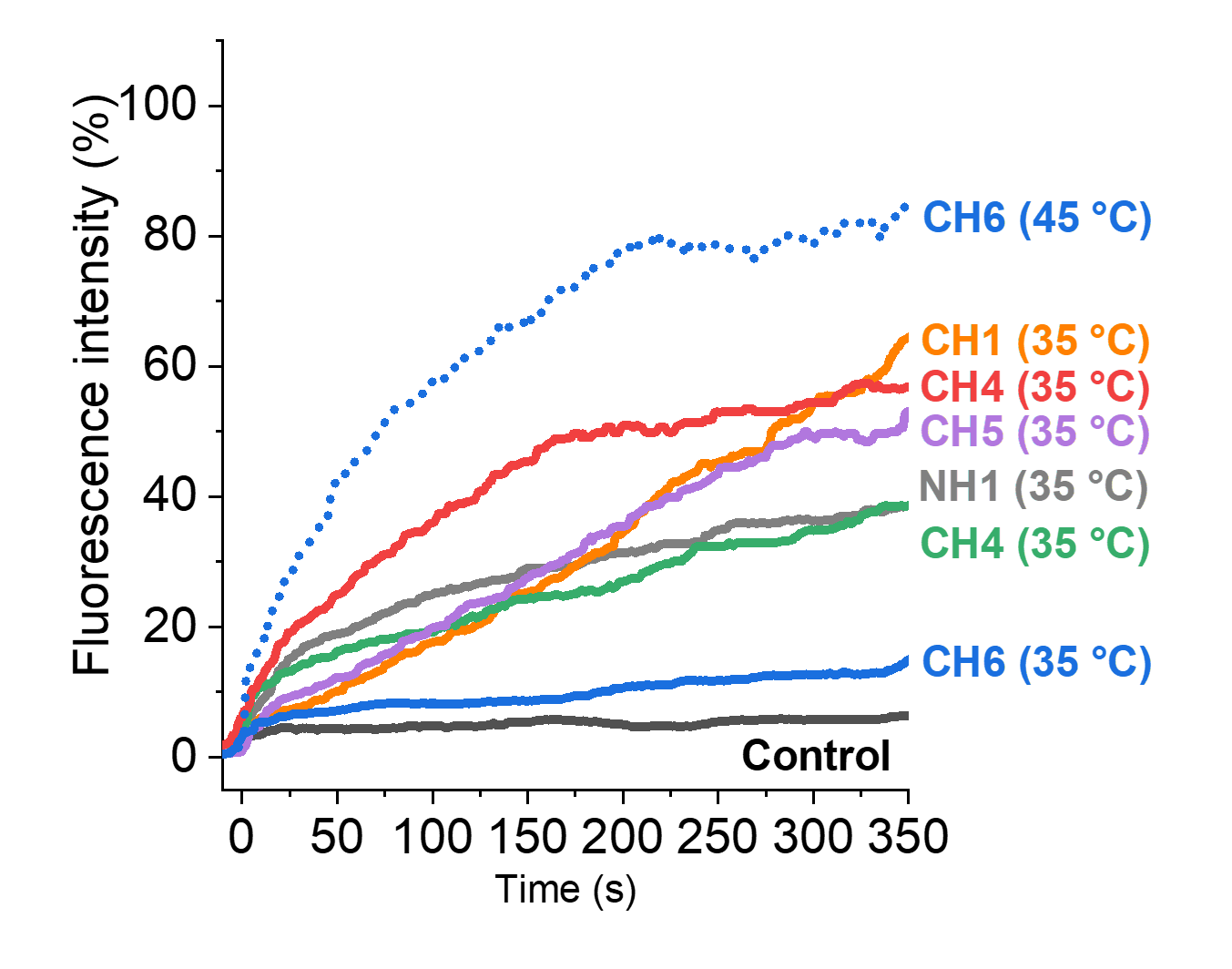


Figure S43. Time-dependent HPTS normalized fluorescence intensity change corresponding to nitrate transport in the DPPC assay at 35 (solid lines) and 45 °C (dashed line).

# Cholesterol-dependent tests

**General procedure for LUVs preparation**

Egg yolk L-α-phosphatidylcholine (EYPC, chloroform solution, 2000 μL) and Cholesterol (0, 15, 35 and 45 mol% relative to EYPC lipids) were added to a 10 mL round-bottomed flask, the solvent was slowly removed under a flow of argon, and the resulting thin film was further dried in a vacuum desiccator for a minimum of 3 h. The lipid film was hydrated with 1000 μL of NaNO_3_ (100 mM in phosphate buffer 10 mM, pH = 6.4) containing 10 μM HPTS (8-hydroxypyrene-1,3,6-trisulfonic acid trisodium salt) for 1h under periodic gentle vertexing. Afterwards, the suspension was submitted to 10 freeze-thaw cycles (bathed in liquid nitrogen and water at 30 °C, respectively). The large multilamellar liposome suspension (1000 μL) was submitted to extrusion (21 extrusions) at room temperature through a 100 nm polycarbonate membrane to afford a suspension of LUVs with an average diameter of around 100 nm (as confirmed by dynamic light scattering measurements). The LUV suspension was separated from extravesicular dye by size exclusion chromatography (stationary phase: Sephadex G-50, mobile phase: phosphate buffer, 100 mM NaNO_3_) and diluted to 7 mL with the same 100 mM NaNO_3_ in PBS buffer.

**Fluorescence measurements**

In a quartz fluorometric cuvette, 100 μL of the above-prepared LUVs has been suspended in 1860 µL sodium phosphate (pH = 6.4) with 100 mM NaGluconate and placed in a fluorescence instrument equipped with a magnetic stirrer and thermostat set at 20 °C. The emission of HPTS at 510 nm was monitored with excitation wavelengths at 403 and 460 nm simultaneously, using a Perkin Elmer FL6500 spectrometer. During the experiment, 20 μL of FCCP (0.108 mol% in DMSO) was added, followed by 20 μL of each compound (4.31 mol% in DMSO) to initiate the transport.


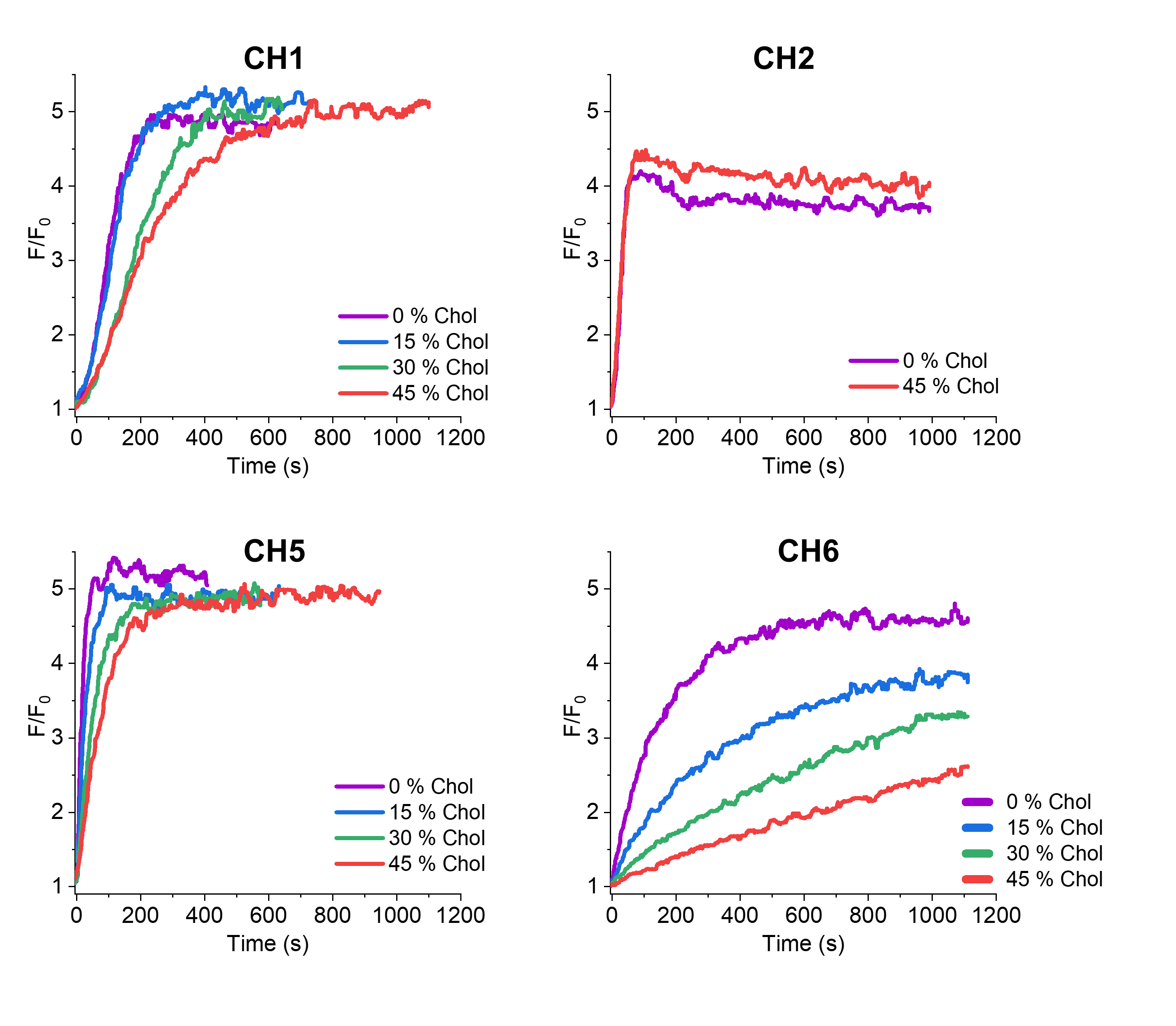


Figure S44. Time-dependent HPTS fluorescence intensity change (F/F_0_) corresponding to nitrate passive transport in the cholesterol-dependent assay for CH donors at 4.31 mol%.

# Variable-temperature nitrate transport experiments

**Fluorescence measurements**

In a quartz fluorometric cuvette, 100 μL of LUVs⸧HPTS and NaNO_3_ 100 mM as the internal salt has been suspended in 1860 µL sodium phosphate (pH = 6.4) with 100 mM NaGluconate and placed in a fluorescence instrument equipped with a magnetic stirrer and thermostat set at different temperatures (from 10 to 45 °C). The emission of HPTS at 510 nm was monitored with excitation wavelengths at 403 and 460 nm simultaneously, using a Perkin Elmer FL6500 spectrometer. During the experiment, 20 μL of FCCP (0.108 mol% in DMSO) was added, followed by 20 μL of each compound (4.31 mol% in DMSO) to initiate the transport.


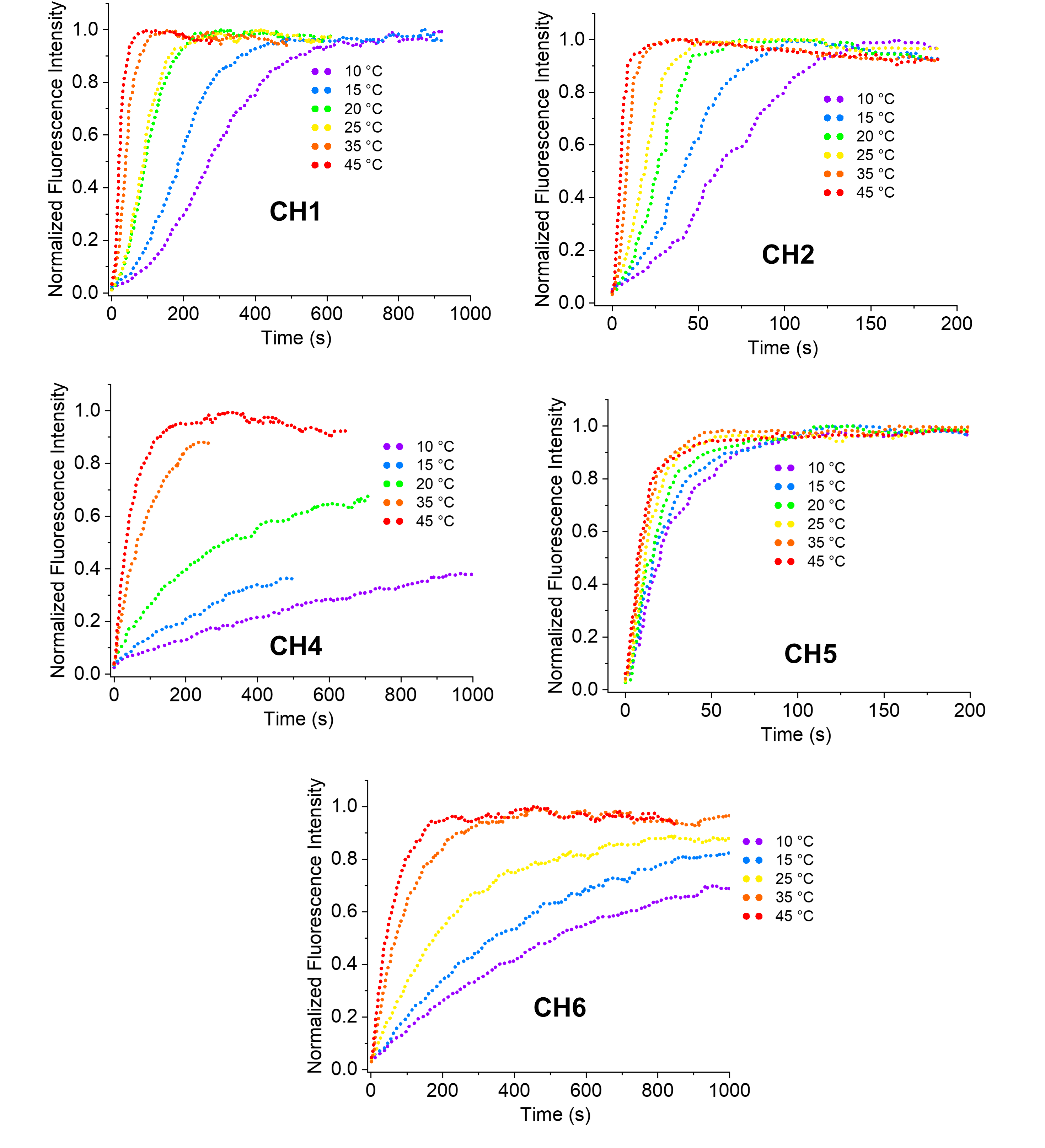


Figure S45. Time-dependent HPTS fluorescence intensity corresponding to variable-temperature nitrate transport.

**Kinetic analysis**

The variable-temperature transport profiles of **CH4**, **CH5** and **CH6** have been fitted to an exponential decay equation (eq. 11).

$$y=y_{0}+A_{1}e^{-\frac{x}{t1}} (11)$$

The pseudo-first-order observed rate constant is then: $k_{obs}=\frac{1}{t1}$

From the Arrhenius equation (eq. 12), plotting ln(*k*_obs_) as a function of 1/T, and using the slope of the linear fitted data, activation energy (*E_a_*) was then calculated using eq. 13.

$$k_{obs}=Ae^{-\frac{Ea}{RT}} (12)$$

$Ea=-slope*R (J{mol}^{-1})$ (13)

Similarly, from the Eyring equation (eq. 14), plotting ln(*k*_obs_/T ) as a function of 1/T, and using the slope and the intercept of the linear fitted data, the activation enthalpy (ΔH^#^) and entropy (ΔS^#^) were calculated using relation 15 and 16, respectively.

$$k_{obs}=\frac{k_{B}T}{h}e^{-\frac{{\Delta H}^{\#}-T{\Delta S}^{\#}}{RT}} (14)$$

${\Delta H}^{\#}=-slope*R \left( J{mol}^{-1} \right) (15)$

$${\Delta S}^{\#}=R\left( intercept-23.76 \right) \left( J{mol}^{-1}K^{-1} \right) (16)$$

Table S1. Activation parameters calculated for **CH4**, **CH5**, and **CH6**.

| Transporter | *Ea* (kJ mol^-1^) | $\boldsymbol{\Delta H}^{\boldsymbol{\#}}$(kJ mol^-1^) | $\boldsymbol{\Delta S}^{\boldsymbol{\#}}$ (J mol^-1^K^-1^) |
| --- | --- | --- | --- |
| CH4 | 63.6 | 61.1 | -84.5 |
| CH5 | 24.7 | 22.3 | -192.6 |
| CH6 | 52.8 | 50.3 | -120.5 |


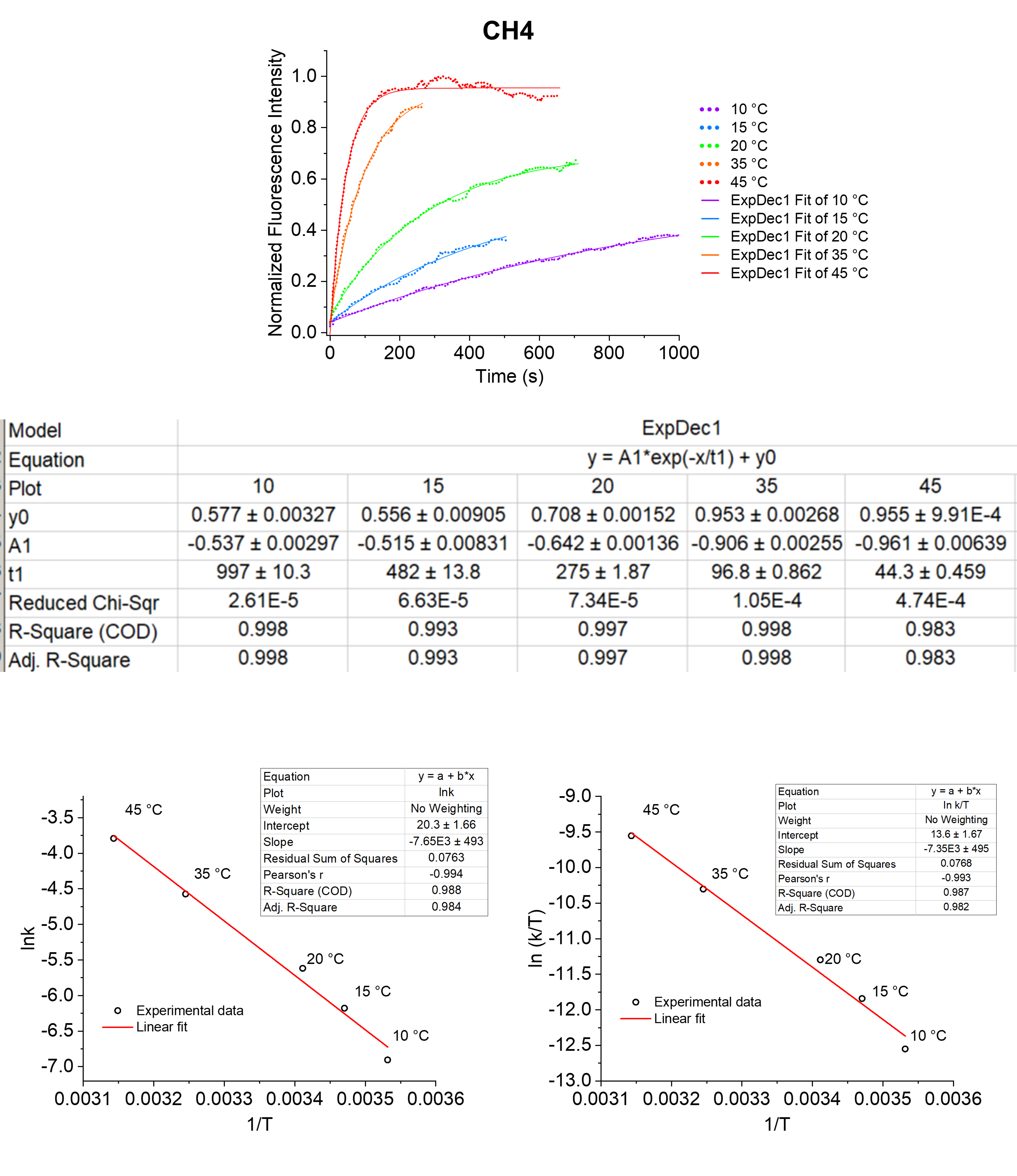


Figure S46. Kinetic analysis for **CH4** mediating nitrate transmembrane transport.


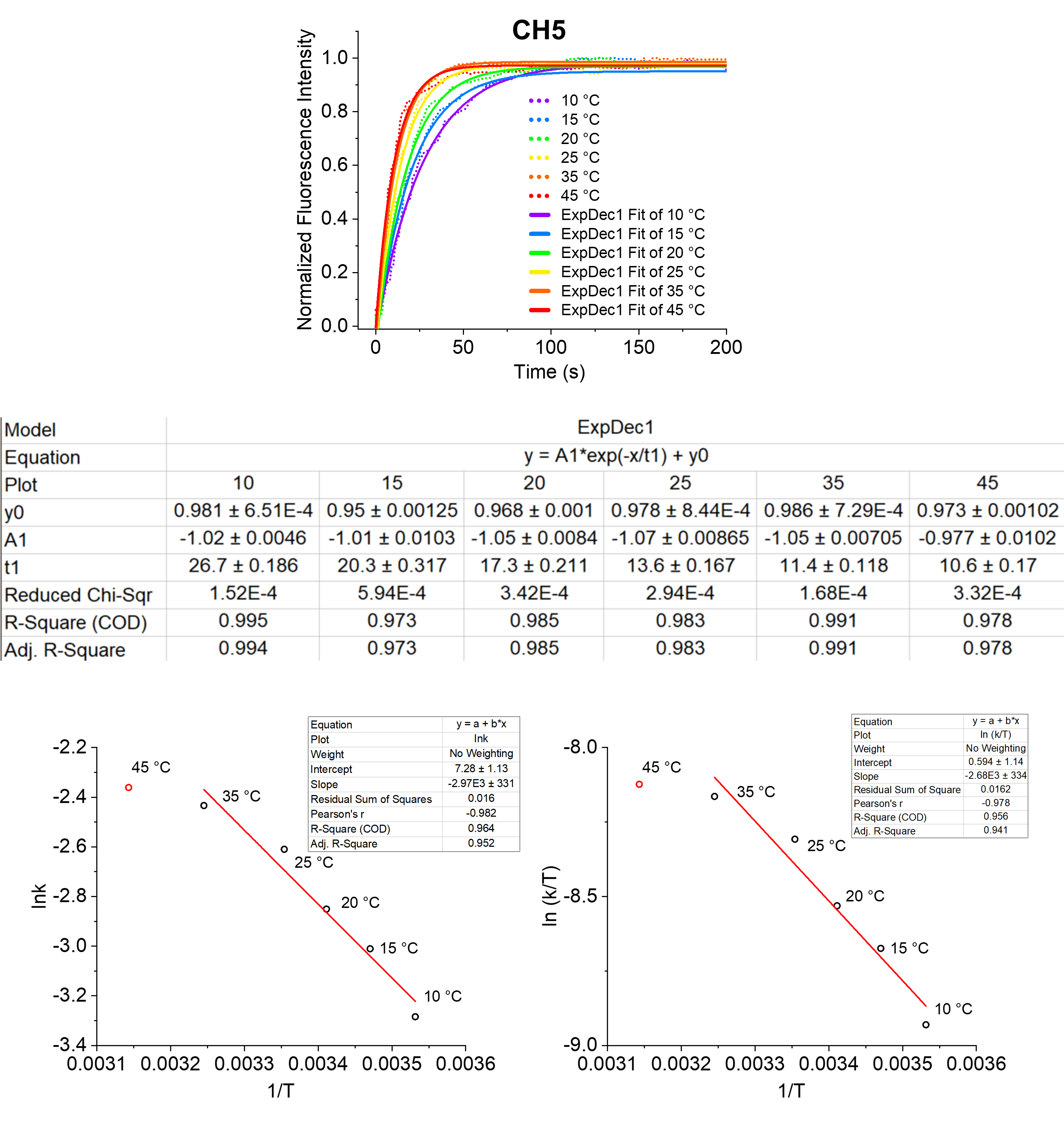


Figure S47. Kinetic analysis for **CH5** mediating nitrate transmembrane transport. Due to reaching transport saturation, the point at 45 °C was excluded.


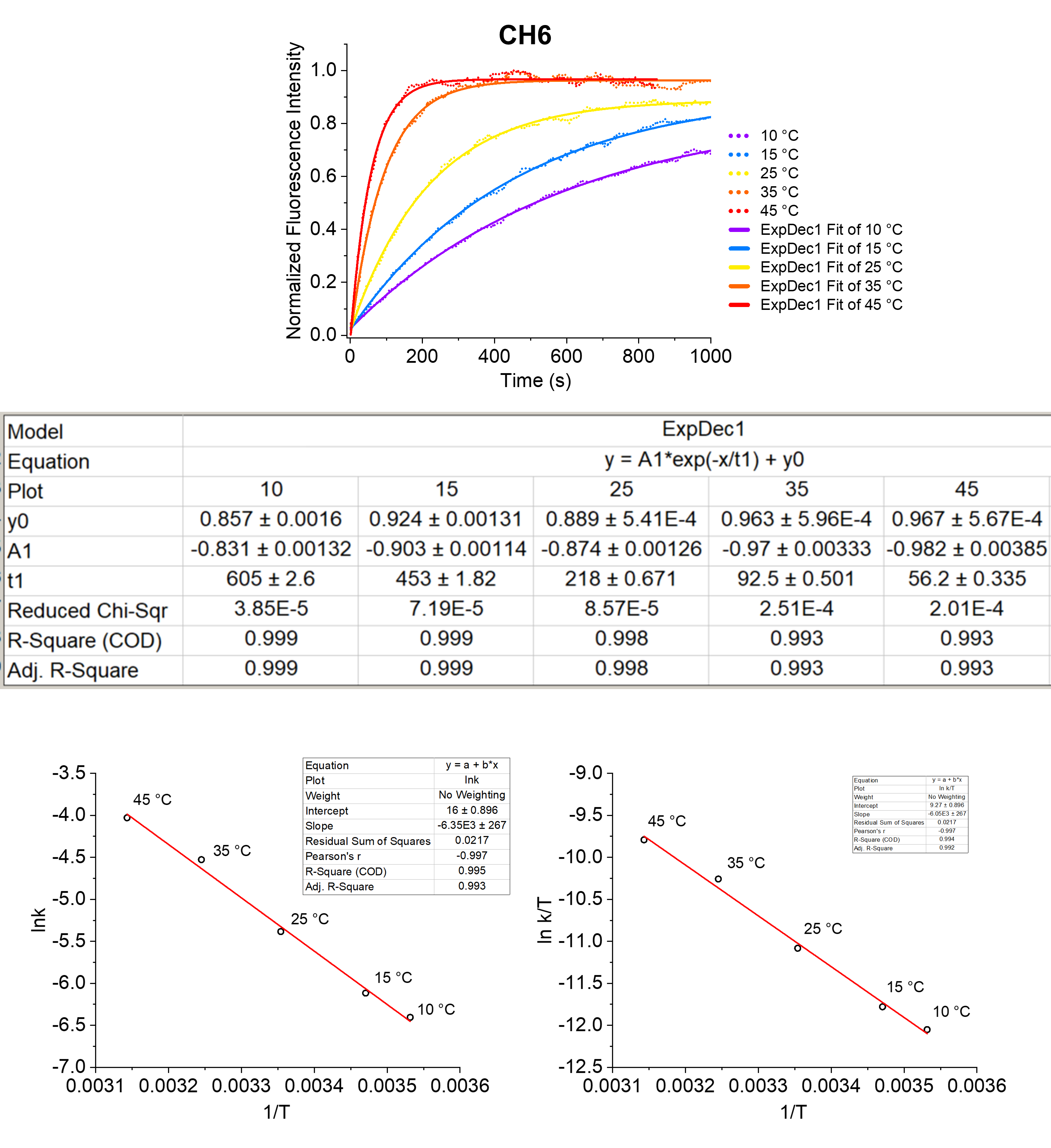


Figure S48. Kinetic analysis for **CH6** mediating nitrate transmembrane transport.

# ^1^H-NMR titration experiments

^1^H NMR titration experiments were conducted on a Bruker Avance III 400 MHz NMR spectrometer in DMSO-*d_6_*, the residual solvent peak (δ = 2.50 ppm) was used as reference.

The anion (chloride, bromide and nitrate) was successively added as *n*-tetrabutylammonium salt to a solution of the receptor (500 µL, 3 mM). Explicitly, the addition of 5 µL of titrant solution increases the amount of TBAX by 2.5 equivalents. Furthermore, an initial ^1^H NMR spectrum of each receptor was recorded prior to the addition of the salt, as a reference. Upon addition of salt, the chemical shift of protons **a** and **b** was monitored during the experiments, and titration was performed until no significant change in the chemical shift of these protons was observed. Compound **CH3,** being completely inactive as an ion transporter, was not subjected to titration.

The values of the chemical shifts were collected from registered spectra, and have been plotted as functions of Host/Guest molar ratio and fitted in the framework of the Nelder-Mead method, using the supramolecular.org platform.^[8,9]^ Complexation constants have been computed in 1:1 receptor to anion binding model.


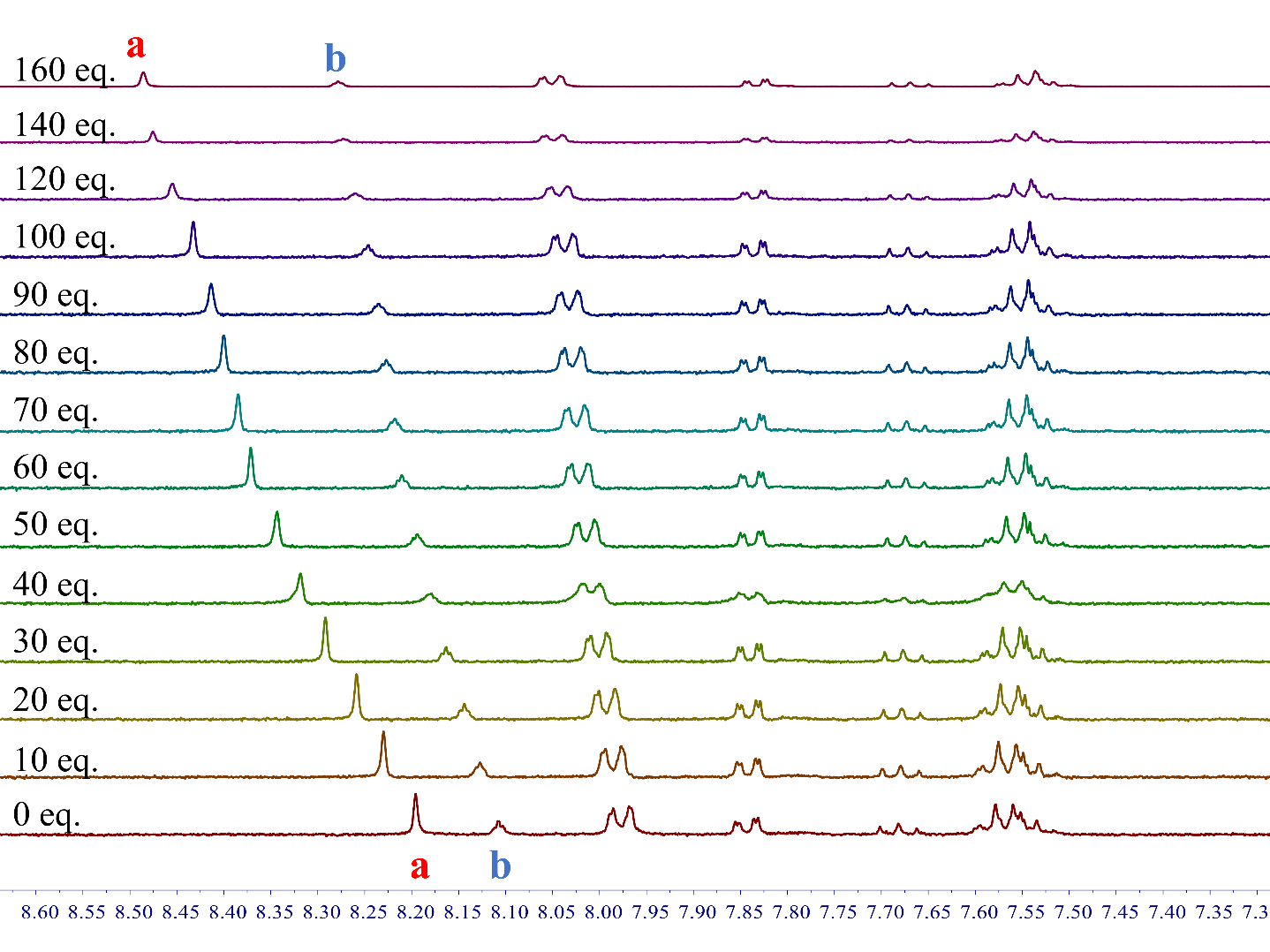


Figure S49: Stacked spectra for the ^1^H NMR titration (400 MHz, DMSO-*d6*) of compound **CH1** (3 mM) with the successive addition of TBACl.


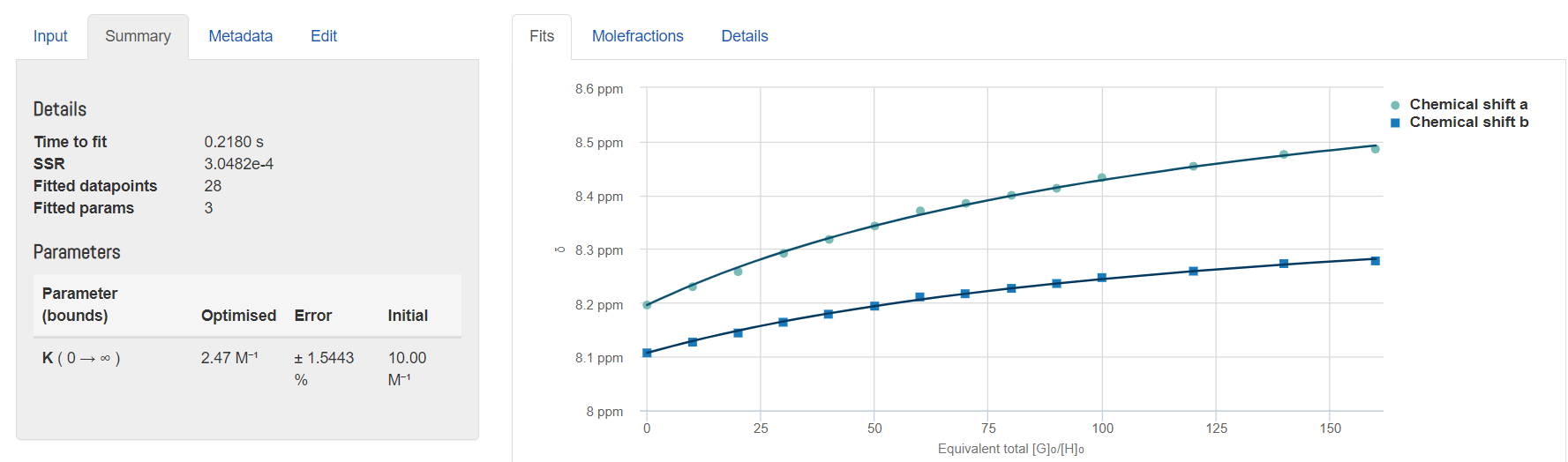


Figure S50: Screenshot of the fitted data plot from supramolecular.org for the titration of **CH1** with TBACl. The binding constant was found to be 2.47 M^-1^ ± 1.54% in 1:1 receptor to anion binding model (left side). The changing pattern of chemical shift with the increasing equivalent TBACl (right side). The Bindfit URL for this experiment is: <http://app.supramolecular.org/bindfit/view/ca652c46-16b3-4676-80a3-bd920cfc88f6>


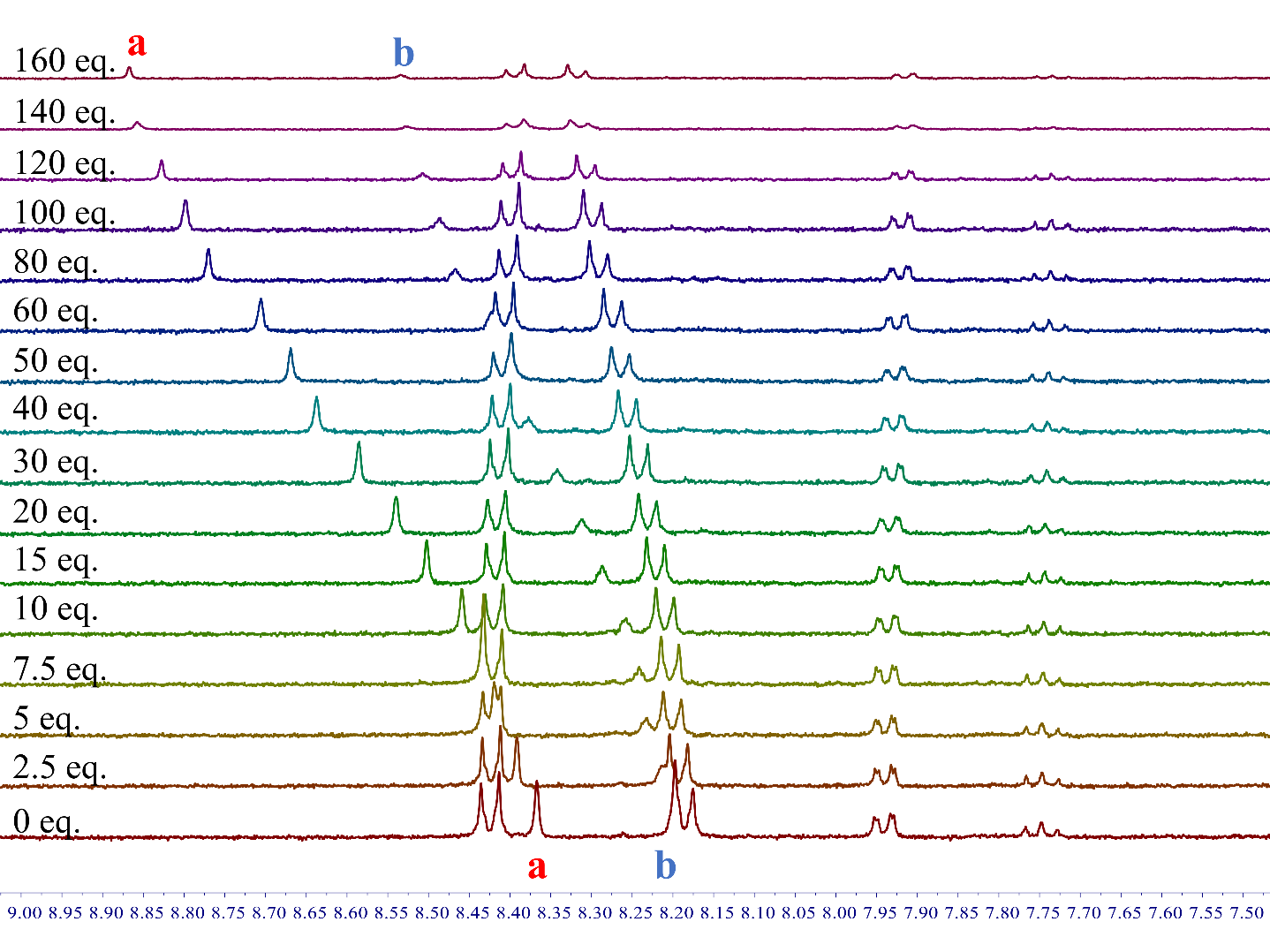


Figure S51: Stacked spectra for the ^1^H NMR titration (400 MHz, DMSO-*d6*) of compound **CH2** (3 mM) with the successive addition of TBACl.


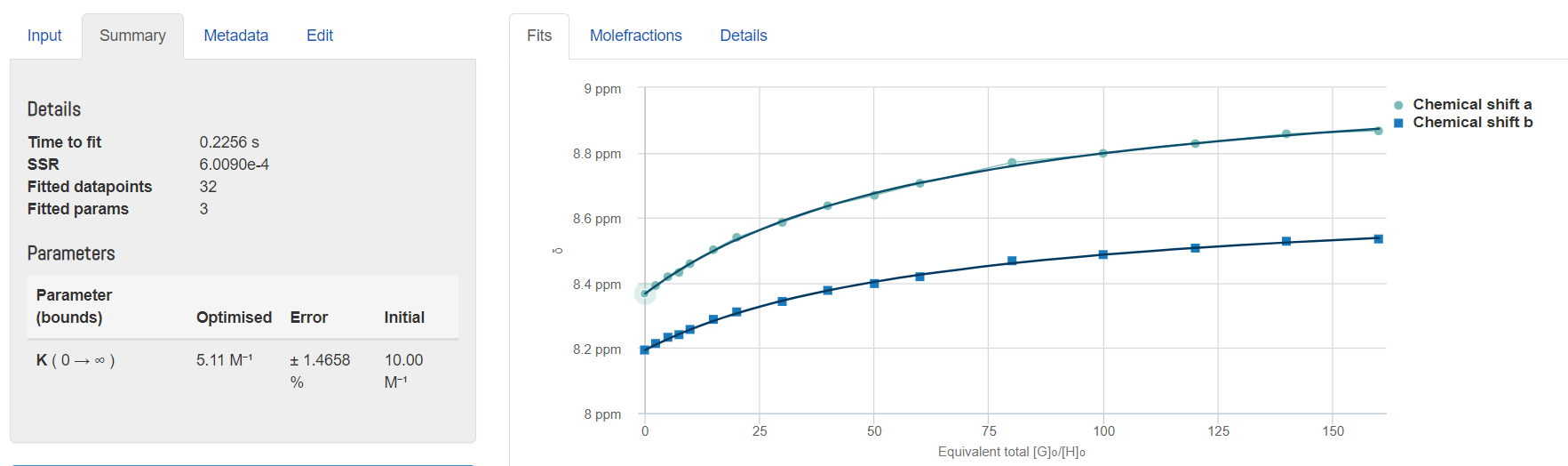


Figure S52: Screenshot of the fitted data plot from supramolecular.org for the titration of **CH2** with TBACl. The binding constant was found to be 5.11 M^-1^ ± 1.47% in 1:1 receptor to anion binding model (left side). The changing pattern of chemical shift with the increasing equivalent TBACl (right side). The Bindfit URL for this experiment is: <http://app.supramolecular.org/bindfit/view/e9539b6a-6f5c-4a06-98b5-d6e52888d220>


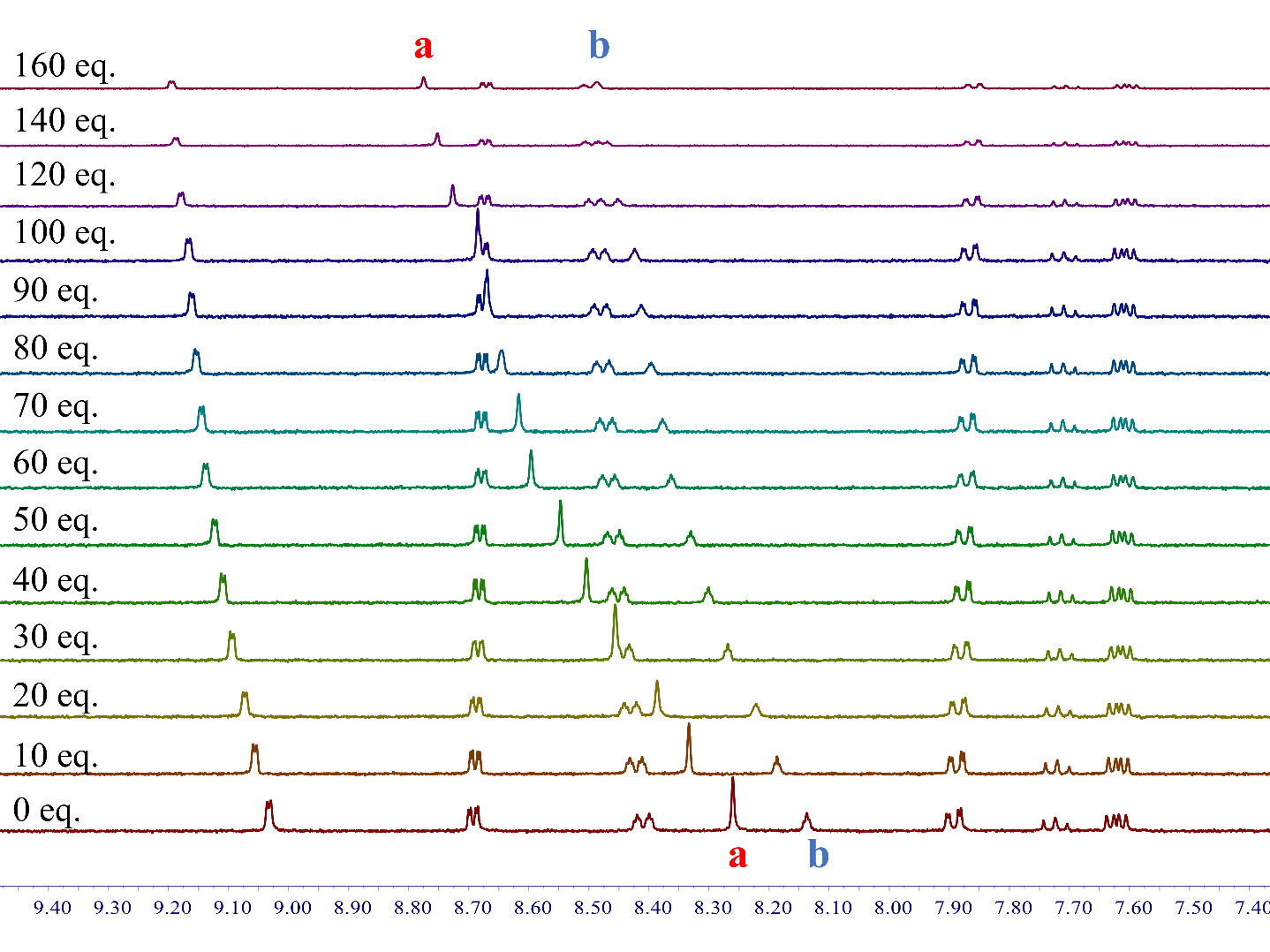


Figure S53: Stacked spectra for the ^1^H NMR titration (400 MHz, DMSO-*d6*) of compound **CH4** (3 mM) with the successive addition of TBACl.


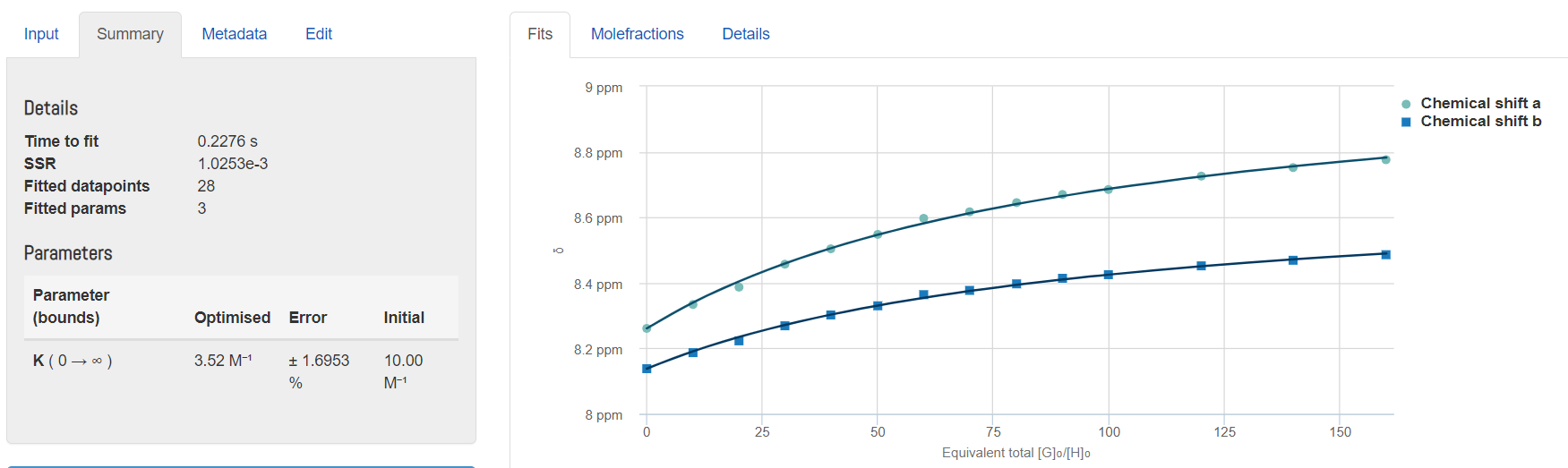


Figure S54: Screenshot of the fitted data plot from supramolecular.org for the titration of **CH4** with TBACl. The binding constant was found to be 3.52 M^-1^ ± 1.70% in 1:1 receptor to anion binding model (left side). The changing pattern of chemical shift with the increasing equivalent TBACl (right side). The Bindfit URL for this experiment is: <http://app.supramolecular.org/bindfit/view/b0661e3e-ac1a-4031-9b98-421e7abbf95d>


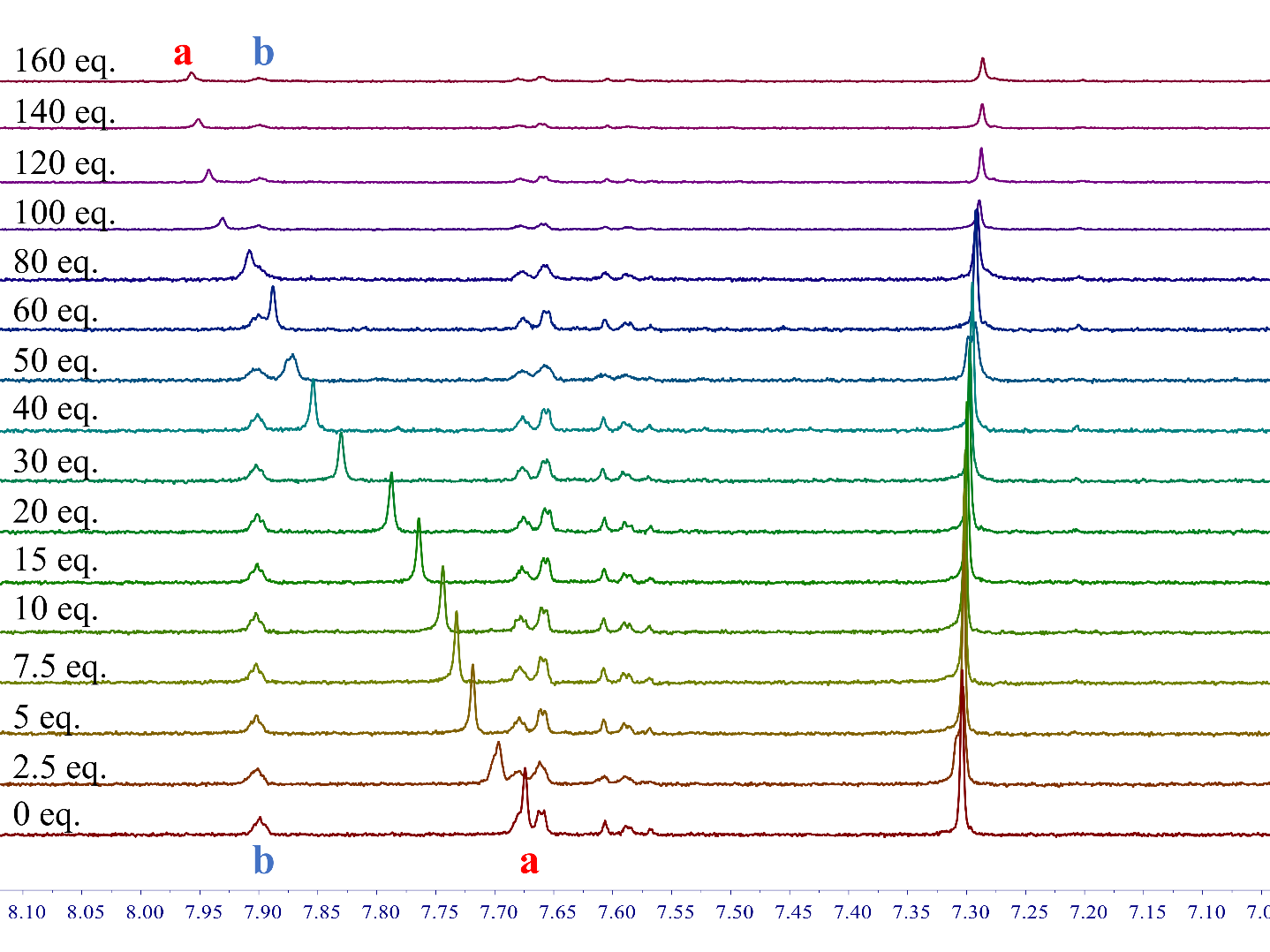


Figure S55: Stacked spectra for the ^1^H NMR titration (400 MHz, DMSO-*d6*) of compound **CH5** (3 mM) with the successive addition of TBACl.


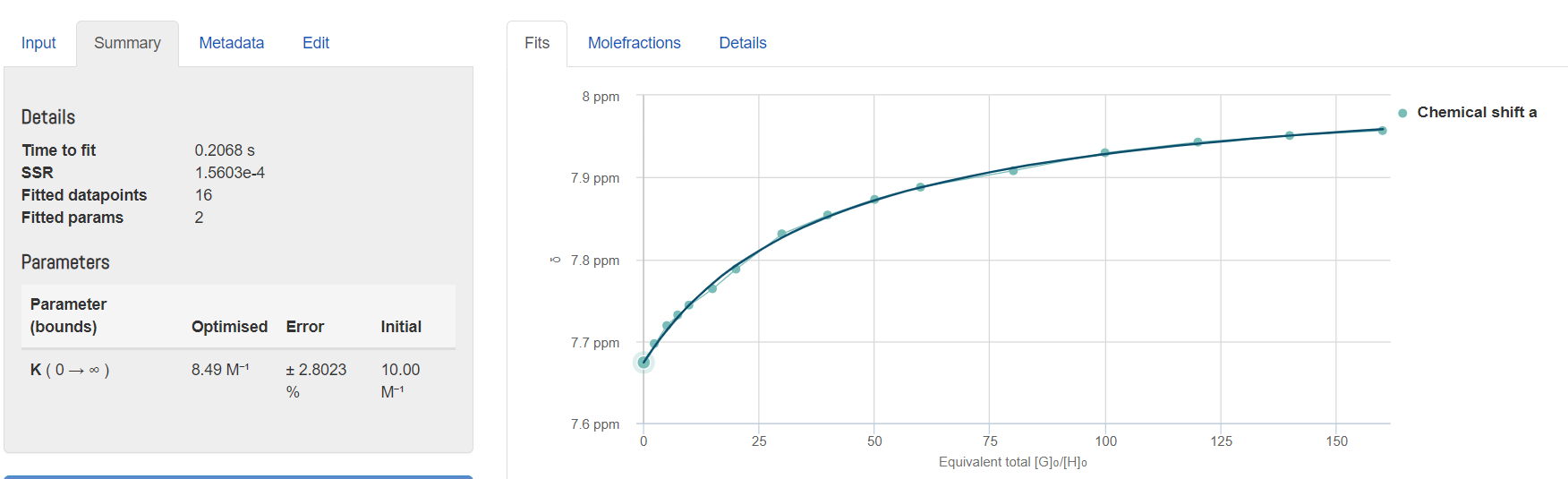


Figure S56: Screenshot of the fitted data plot from supramolecular.org for the titration of **CH5** with TBACl. The binding constant was found to be 8.49 M^-1^ ± 2.80% in 1:1 receptor to anion binding model (left side). The changing pattern of chemical shift with the increasing equivalent TBACl (right side). The Bindfit URL for this experiment is: <http://app.supramolecular.org/bindfit/view/82344861-9234-4e94-b8c5-30a7c291b296>


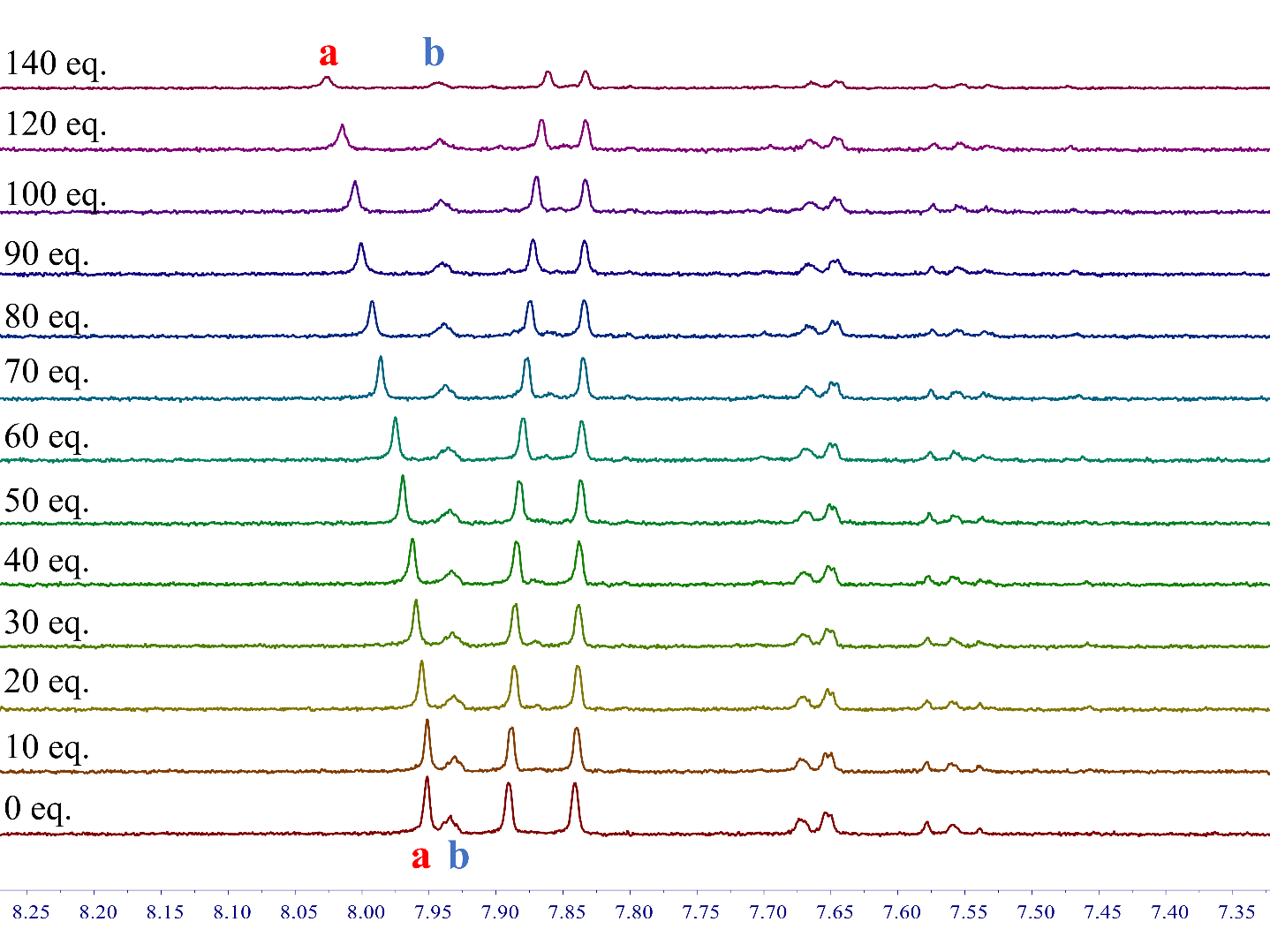


Figure S57: Stacked spectra for the ^1^H NMR titration (400 MHz, DMSO-*d6*) of compound **CH6** (3 mM) with the successive addition of TBACl.


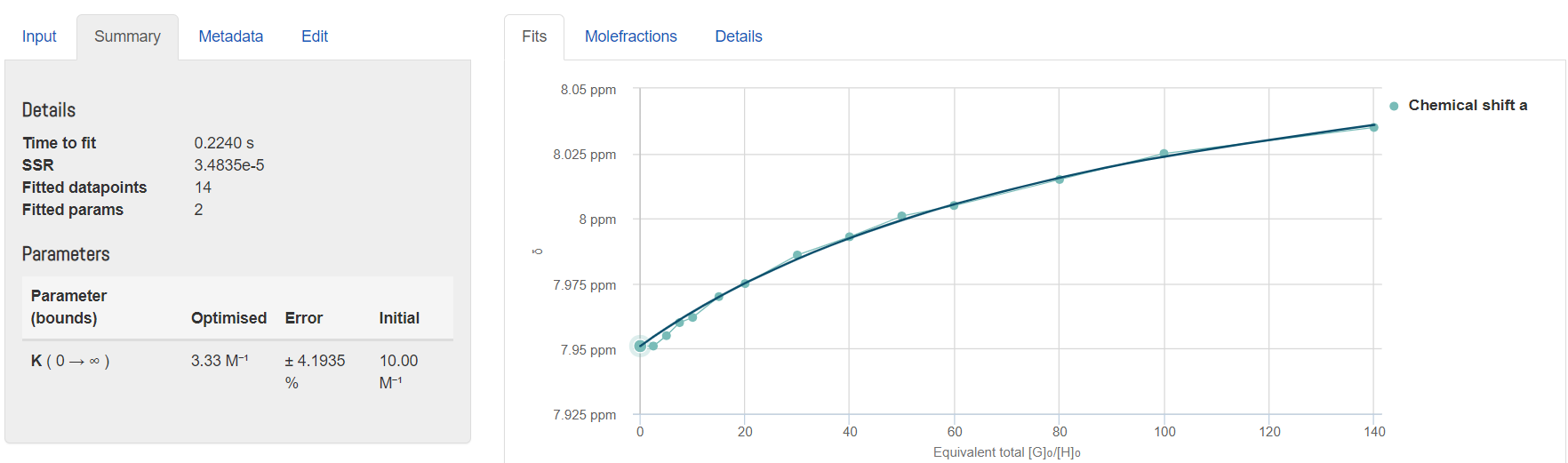


Figure S58: Screenshot of the fitted data plot from supramolecular.org for the titration of **CH6** with TBACl. The binding constant was found to be 3.33 M^-1^ ± 4.19% in 1:1 receptor to anion binding model (left side). The changing pattern of chemical shift with the increasing equivalent TBACl (right side). The Bindfit URL for this experiment is: <http://app.supramolecular.org/bindfit/view/3d8b5418-33b4-4d04-8672-be754052eb9e>


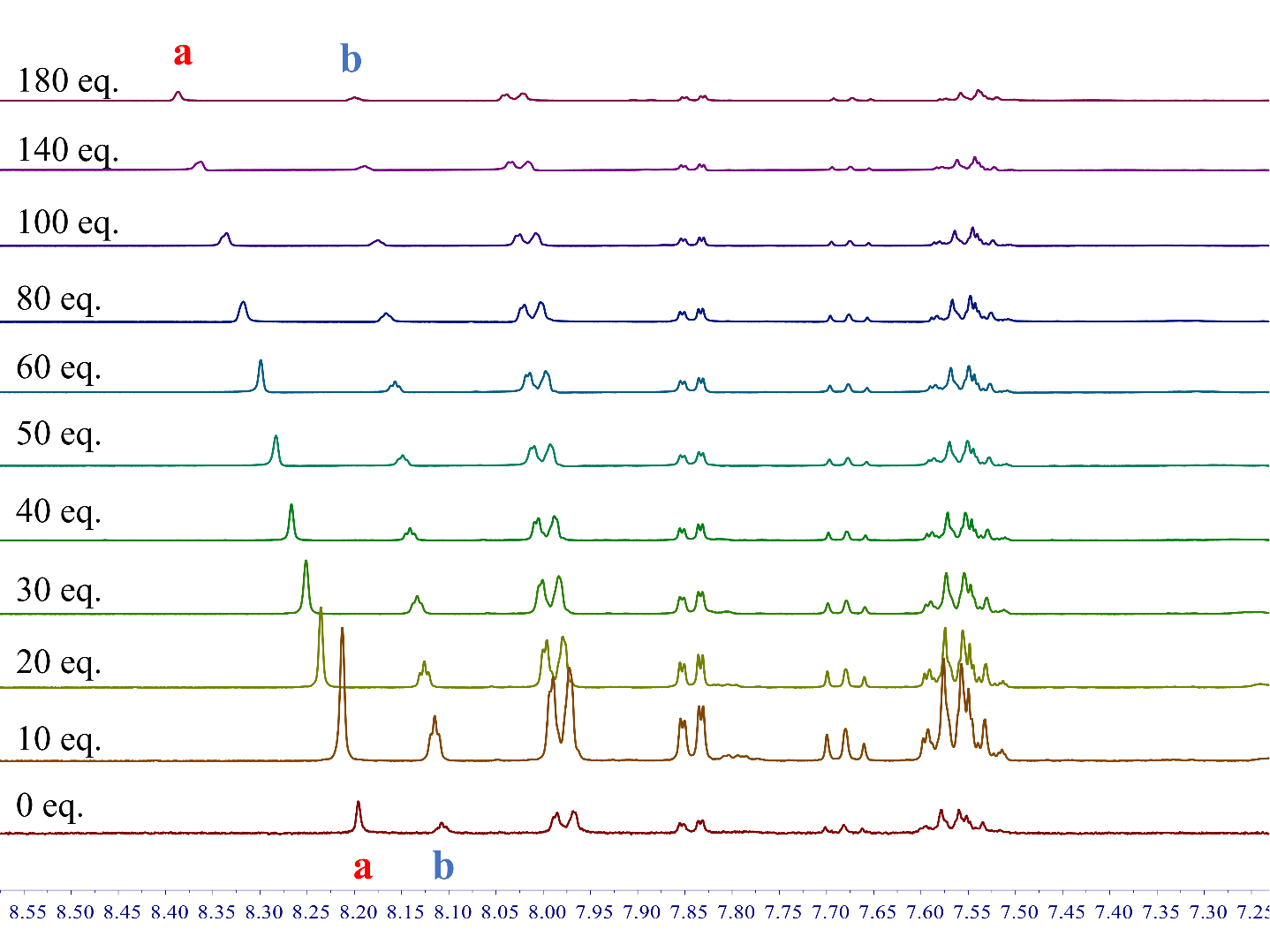


Figure S59: Stacked spectra for the ^1^H NMR titration (400 MHz, DMSO-*d6*) of compound **CH1** (3 mM) with the successive addition of TBABr.


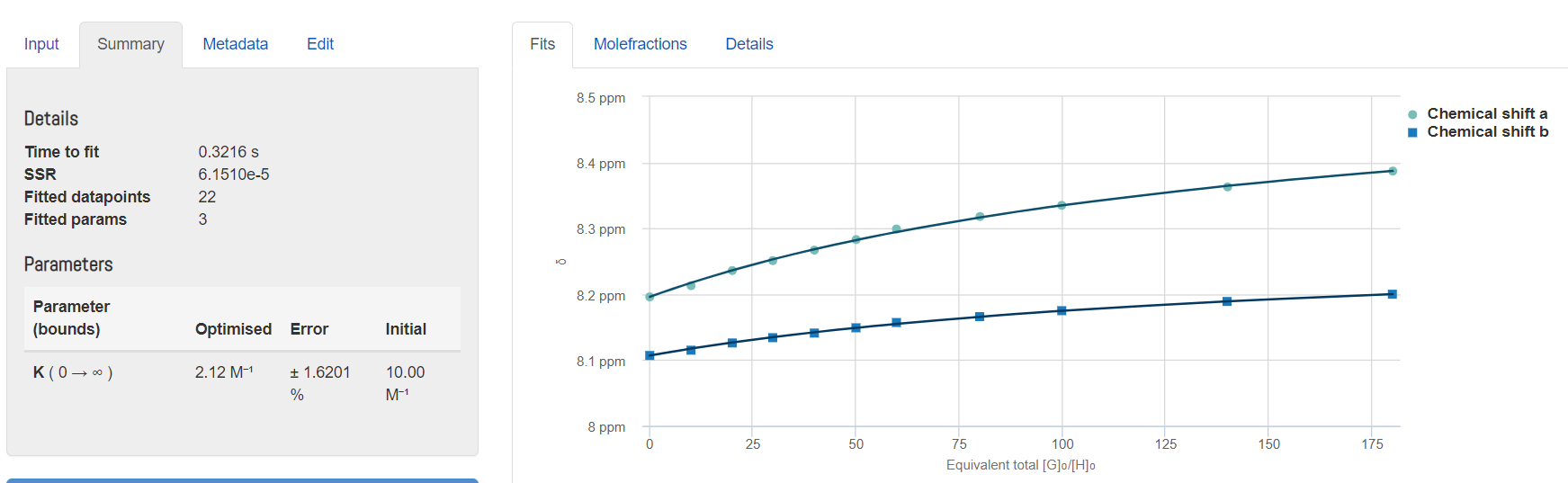


Figure S60: Screenshot of the fitted data plot from supramolecular.org for the titration of **CH1** with TBABr. The binding constant was found to be 2.12 M^-1^ ± 1.62% in 1:1 receptor to anion binding model (left side). The changing pattern of chemical shift with the increasing equivalent TBABr (right side). The Bindfit URL for this experiment is: <http://app.supramolecular.org/bindfit/view/4e900ecc-12a8-422a-9276-18f5d968dd61>


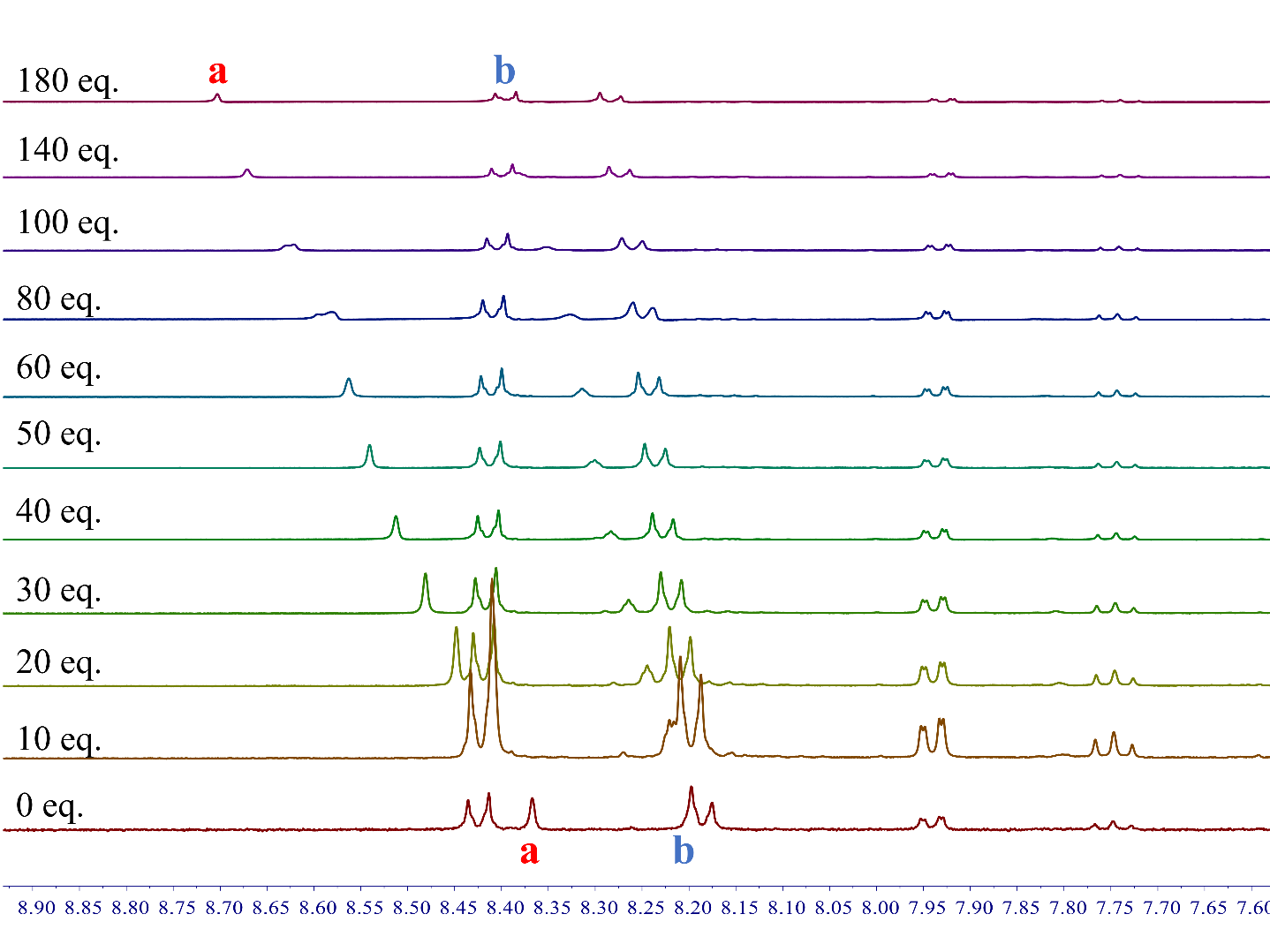


Figure S61: Stacked spectra for the ^1^H NMR titration (400 MHz, DMSO-*d6*) of compound **CH2** (3 mM) with the successive addition of TBABr.


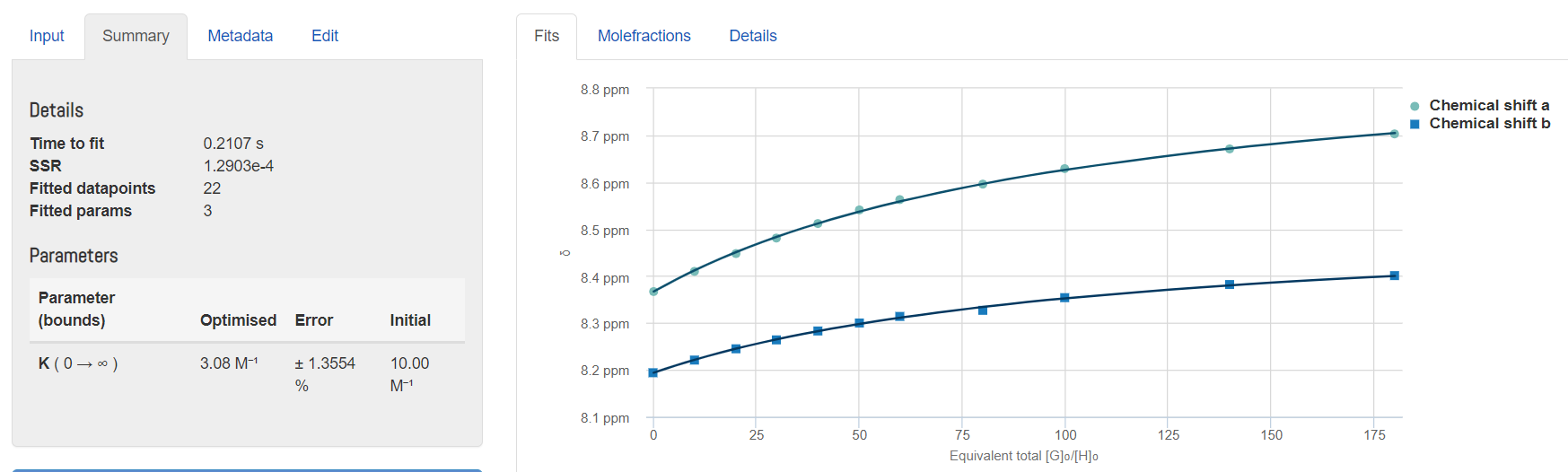


Figure S62: Screenshot of the fitted data plot from supramolecular.org for the titration of **CH2** with TBABr. The binding constant was found to be 3.08 M^-1^ ± 1.35% in 1:1 receptor to anion binding model (left side). The changing pattern of chemical shift with the increasing equivalent TBABr (right side). The Bindfit URL for this experiment is: <http://app.supramolecular.org/bindfit/view/af8c7b67-7be5-4ca2-9696-3ed9bd83ec42>


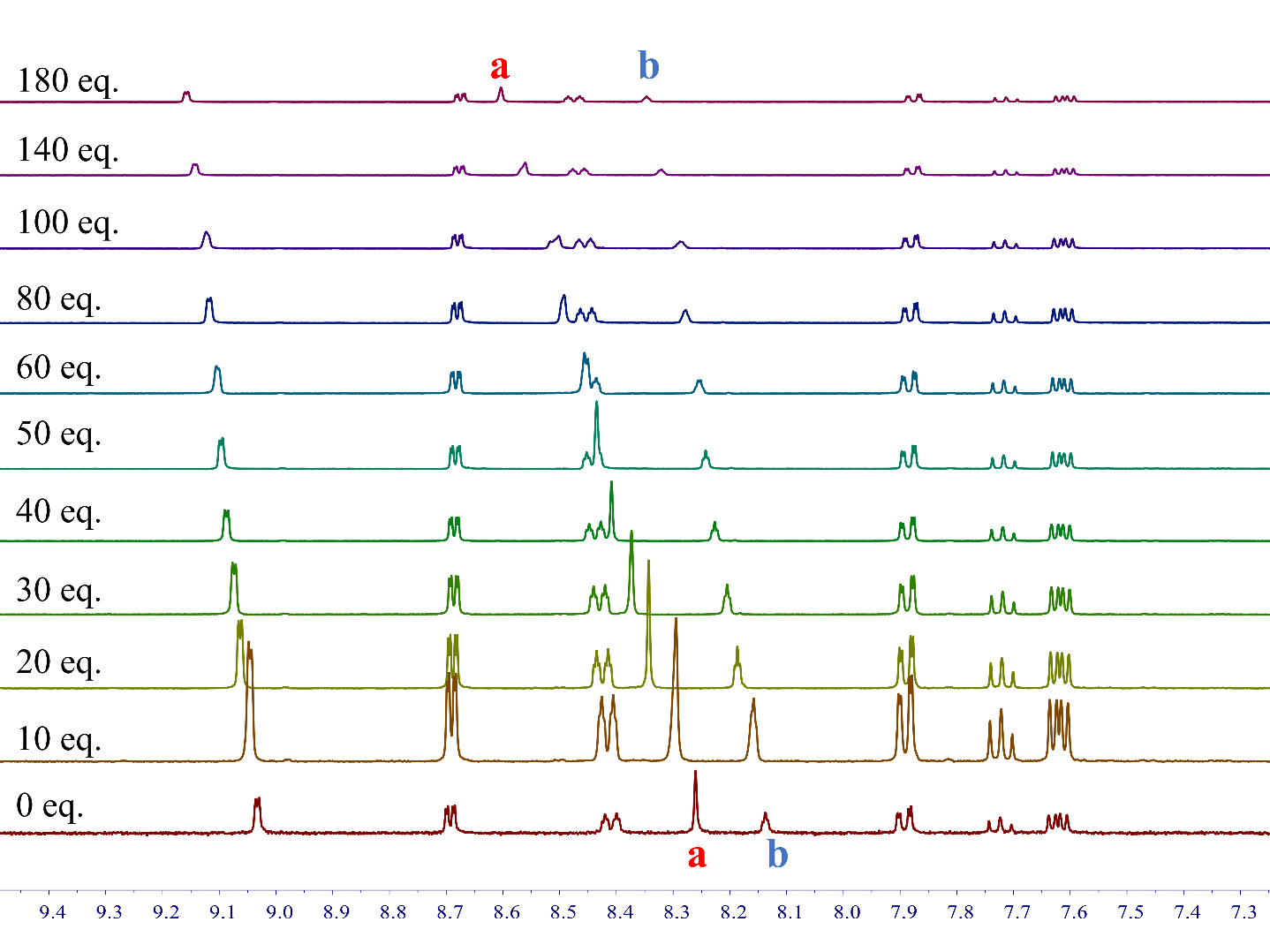


Figure S63: Stacked spectra for the ^1^H NMR titration (400 MHz, DMSO-*d6*) of compound **CH4** (3 mM) with the successive addition of TBABr.


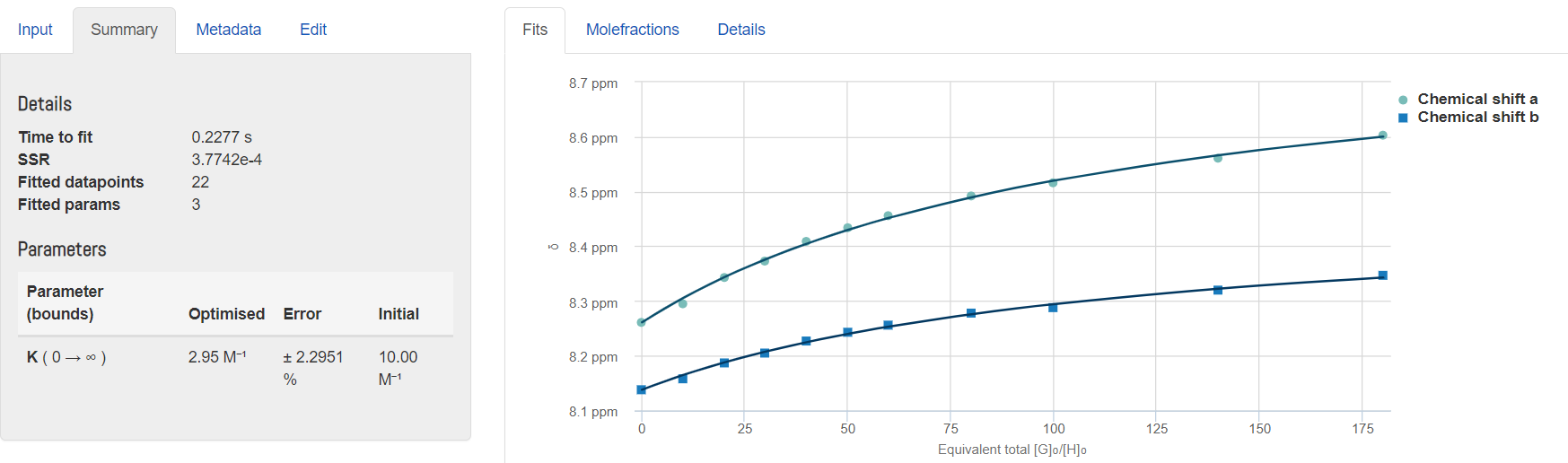


Figure S64: Screenshot of the fitted data plot from supramolecular.org for the titration of **CH4** with TBABr. The binding constant was found to be 2.95 M^-1^ ± 2.29% in 1:1 receptor to anion binding model (left side). The changing pattern of chemical shift with the increasing equivalent TBABr (right side). The Bindfit URL for this experiment is: <http://app.supramolecular.org/bindfit/view/09abf2a6-cfc1-47eb-ab96-e34feee015a9>


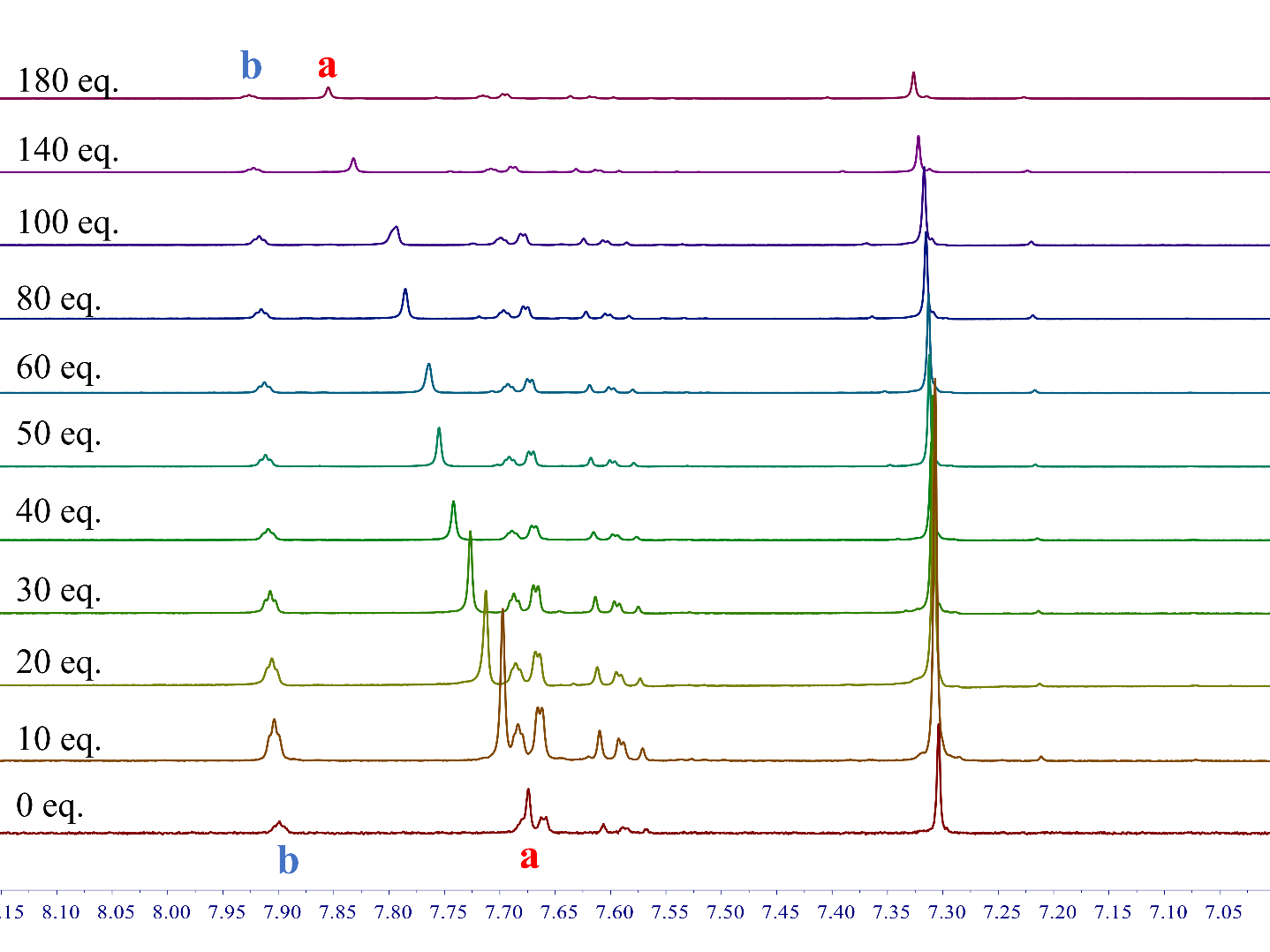


Figure S65: Stacked spectra for the ^1^H NMR titration (400 MHz, DMSO-*d6*) of compound **CH5** (3 mM) with the successive addition of TBABr.


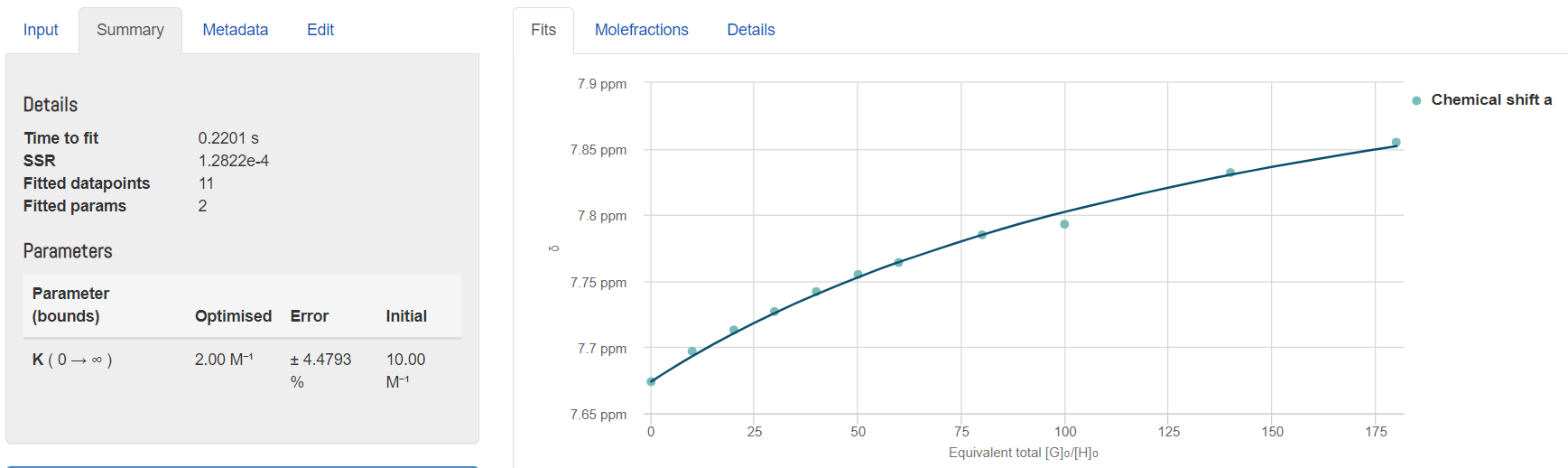


Figure S66: Screenshot of the fitted data plot from supramolecular.org for the titration of **CH5** with TBABr. The binding constant was found to be 2.00 M^-1^ ± 4.48% in 1:1 receptor to anion binding model (left side). The changing pattern of chemical shift with the increasing equivalent TBABr (right side). The Bindfit URL for this experiment is: <http://app.supramolecular.org/bindfit/view/c532d0b4-33a6-4b12-819d-d3e772c1903c>


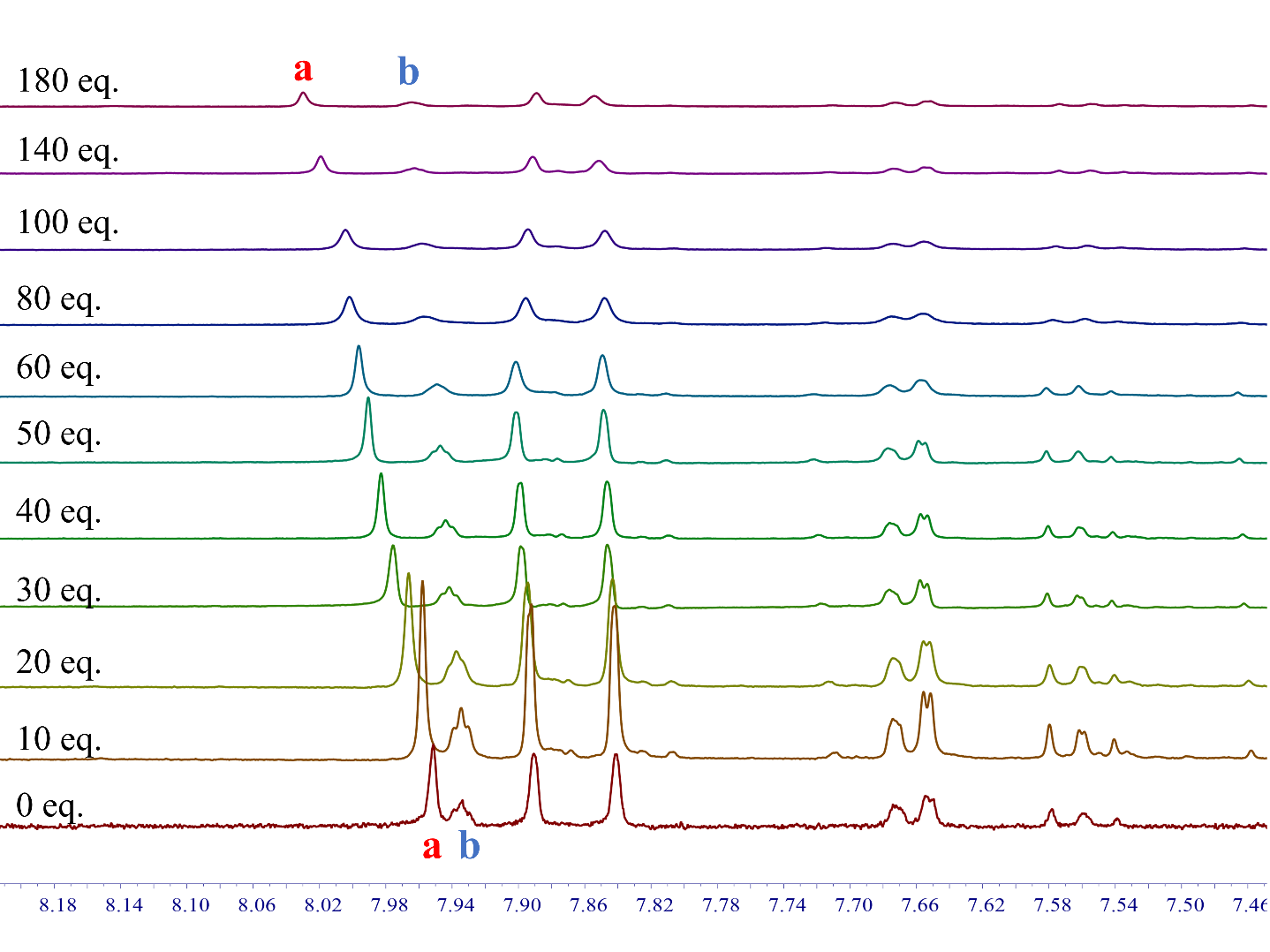


Figure S67: Stacked spectra for the ^1^H NMR titration (400 MHz, DMSO-*d6*) of compound **CH6** (3 mM) with the successive addition of TBABr.


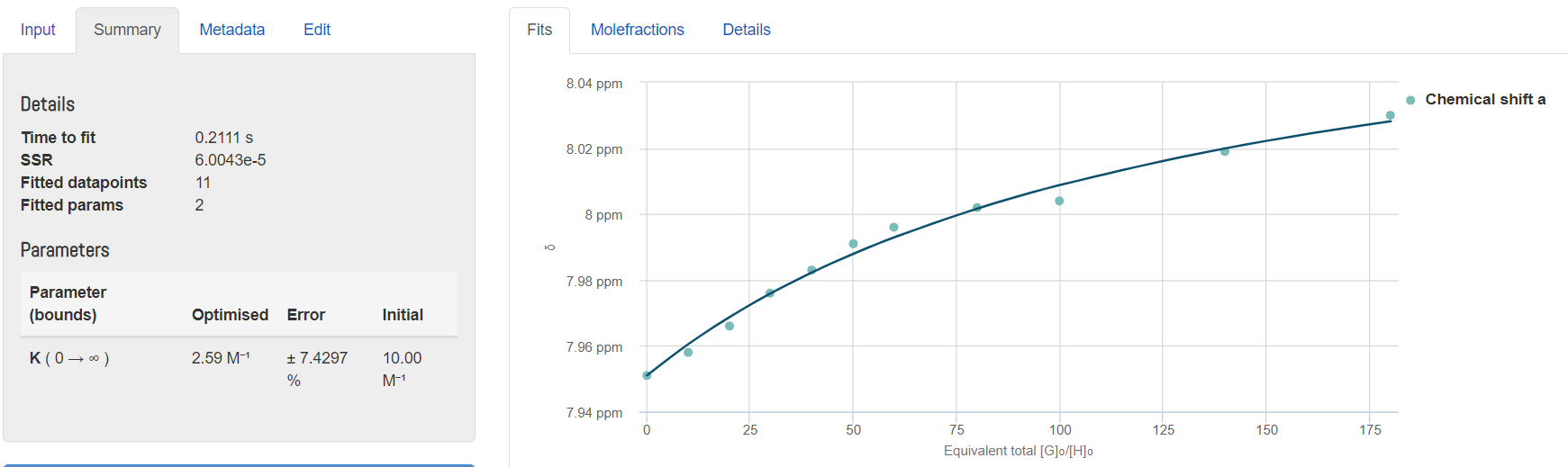


Figure S68: Screenshot of the fitted data plot from supramolecular.org for the titration of **CH6** with TBABr. The binding constant was found to be 2.59 M^-1^ ± 7.43% in 1:1 receptor to anion binding model (left side). The changing pattern of chemical shift with the increasing equivalent TBABr (right side). The Bindfit URL for this experiment is: <http://app.supramolecular.org/bindfit/view/af6db2e3-1d0b-499c-b3af-a9671e36ada1>


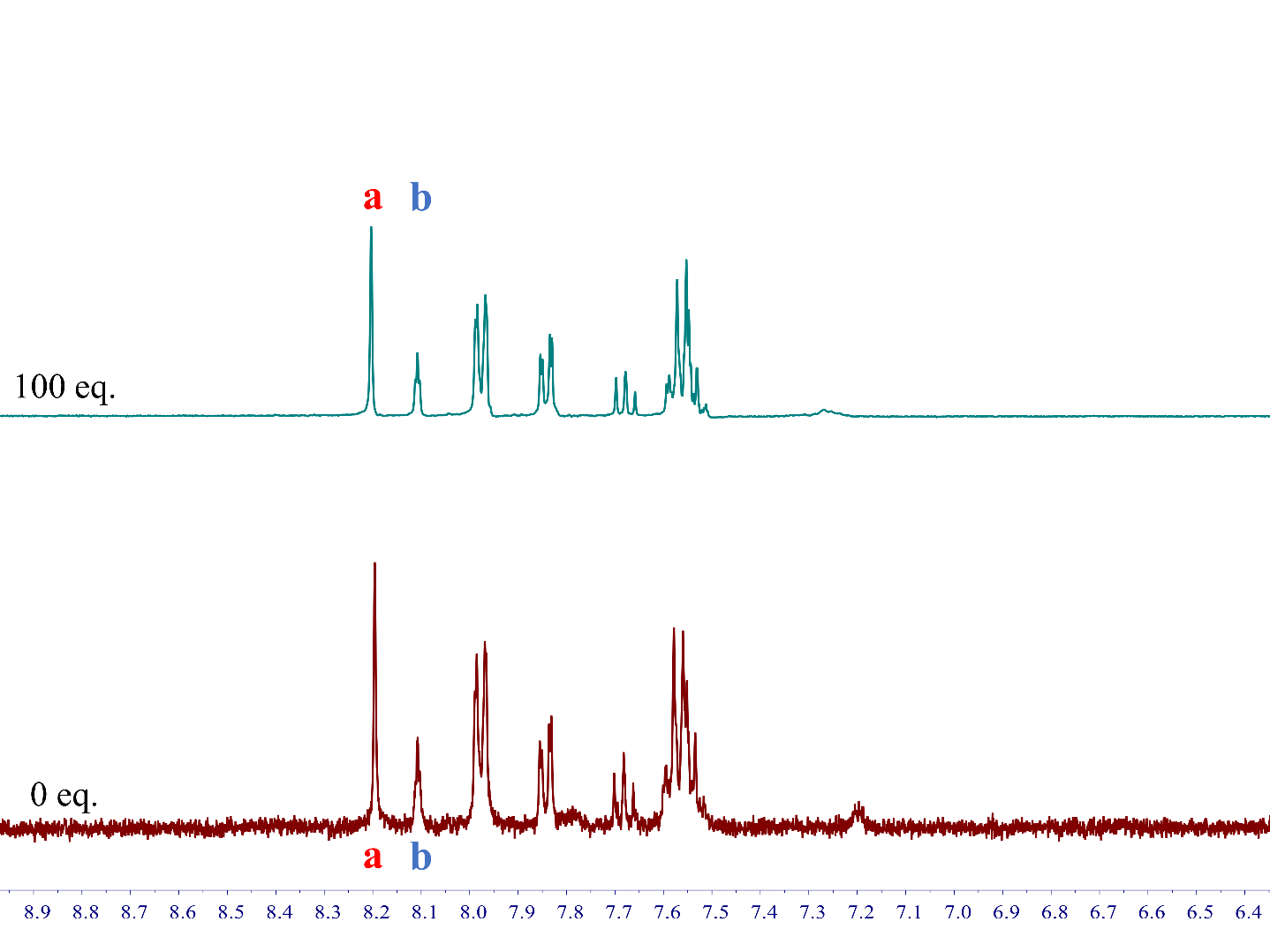


Figure S69: ^1^H NMR spectra (400 MHz, DMSO-*d6*) showing no significant change of the chemical shift of the protons of compound **CH1** (3 mM) upon addition of 100 eq. of TBANO3.


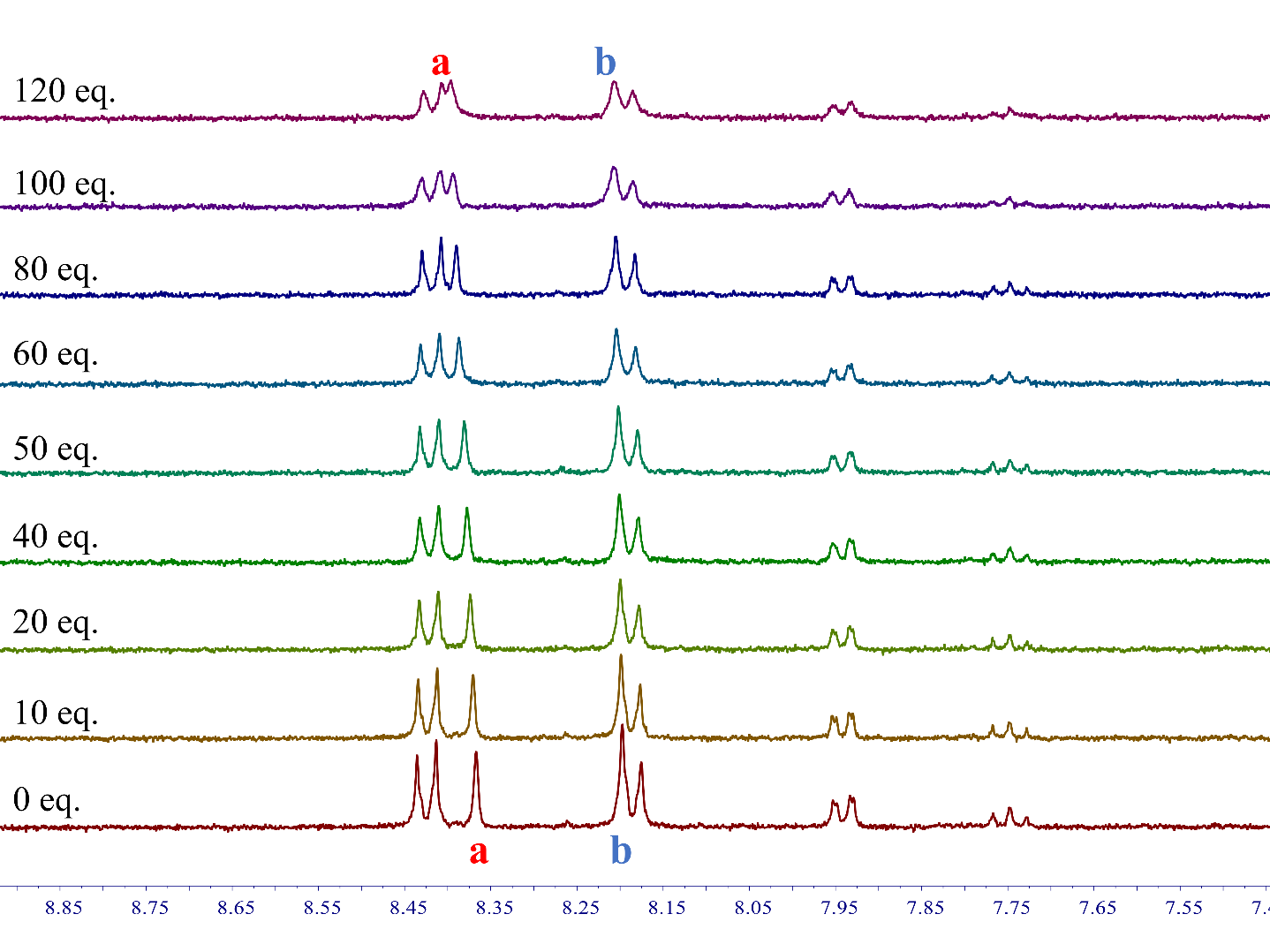


Figure S70: Stacked spectra for the ^1^H NMR titration (400 MHz, DMSO-*d6*) of compound **CH2** (3 mM) with the successive addition of TBANO3.


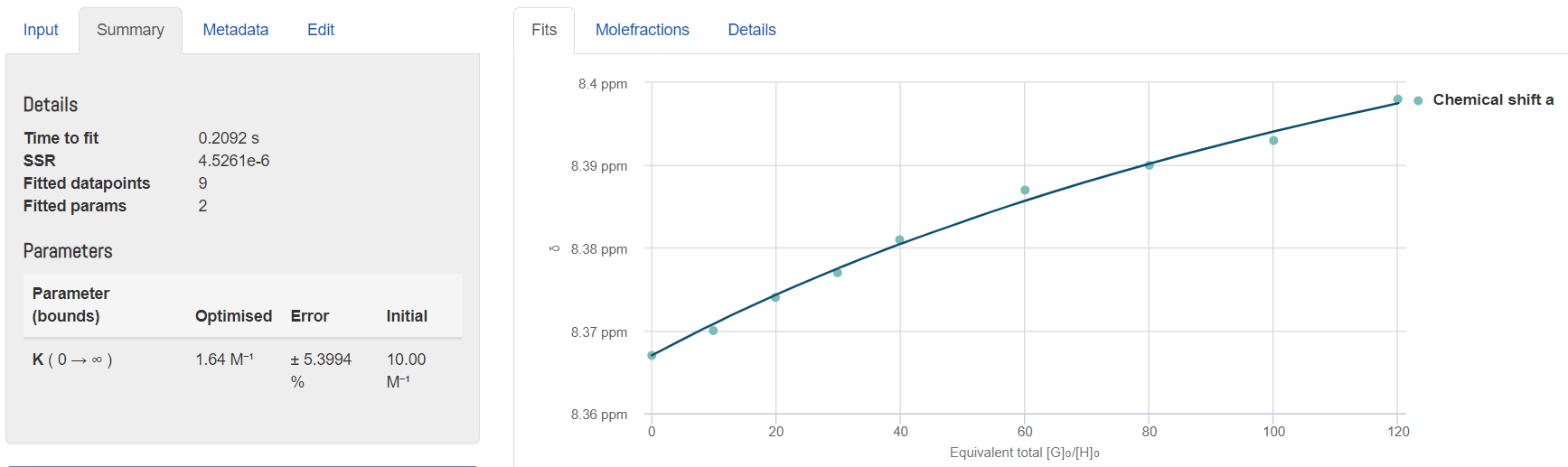


Figure S71: Screenshot of the fitted data plot from supramolecular.org for the titration of **CH2** with TBANO3. The binding constant was found to be 1.64 M^-1^ ± 5.40% in 1:1 receptor to anion binding model (left side). The changing pattern of chemical shift with the increasing equivalent TBABr (right side). The Bindfit URL for this experiment is: <http://app.supramolecular.org/bindfit/view/1c094357-dd3d-4819-b9fc-048280000567>


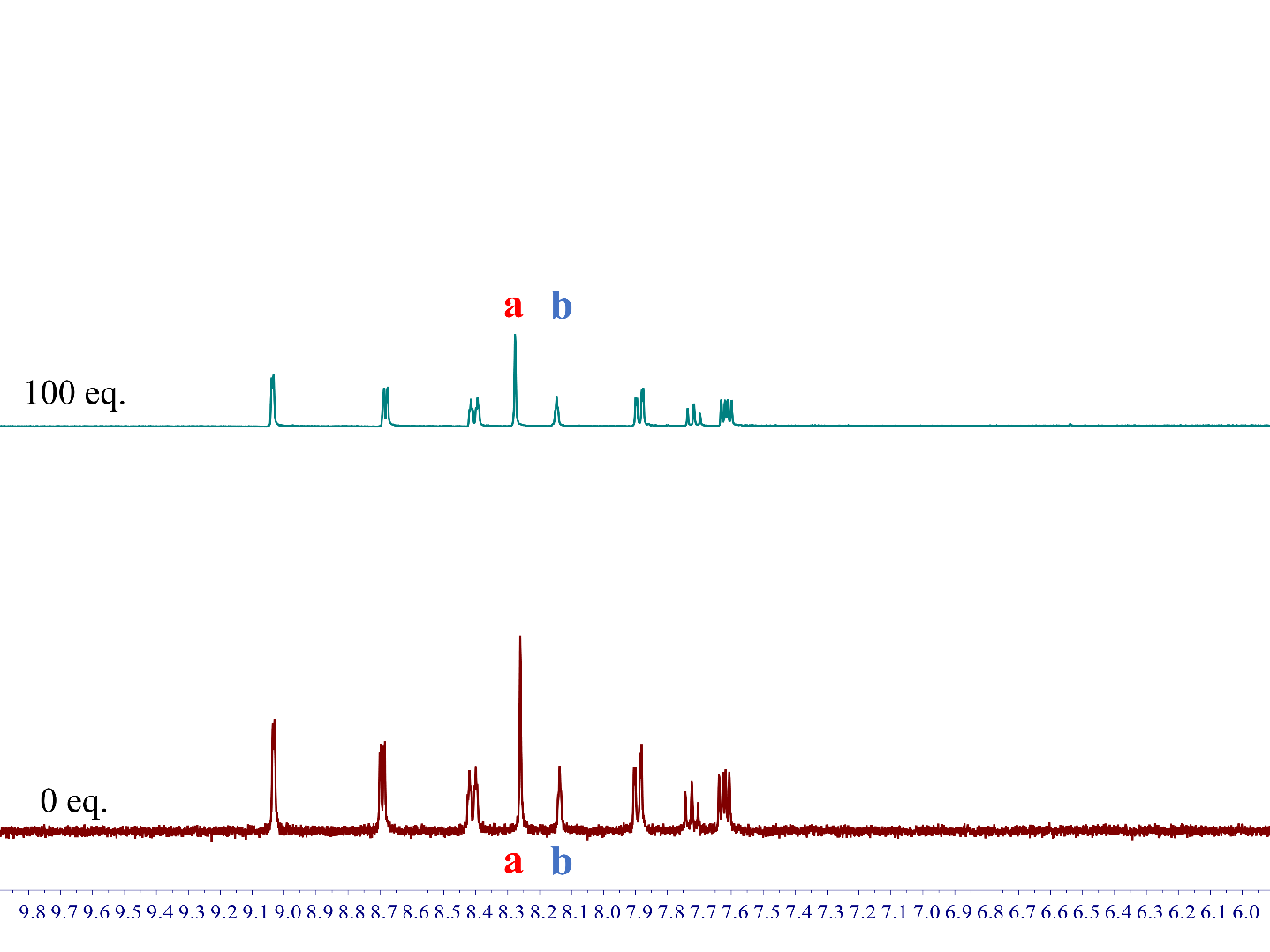


Figure S72: ^1^H NMR spectra (400 MHz, DMSO-*d6*) showing no significant change of the chemical shift of the protons of compound **CH4** (3 mM) upon addition of 100 eq. of TBANO3.


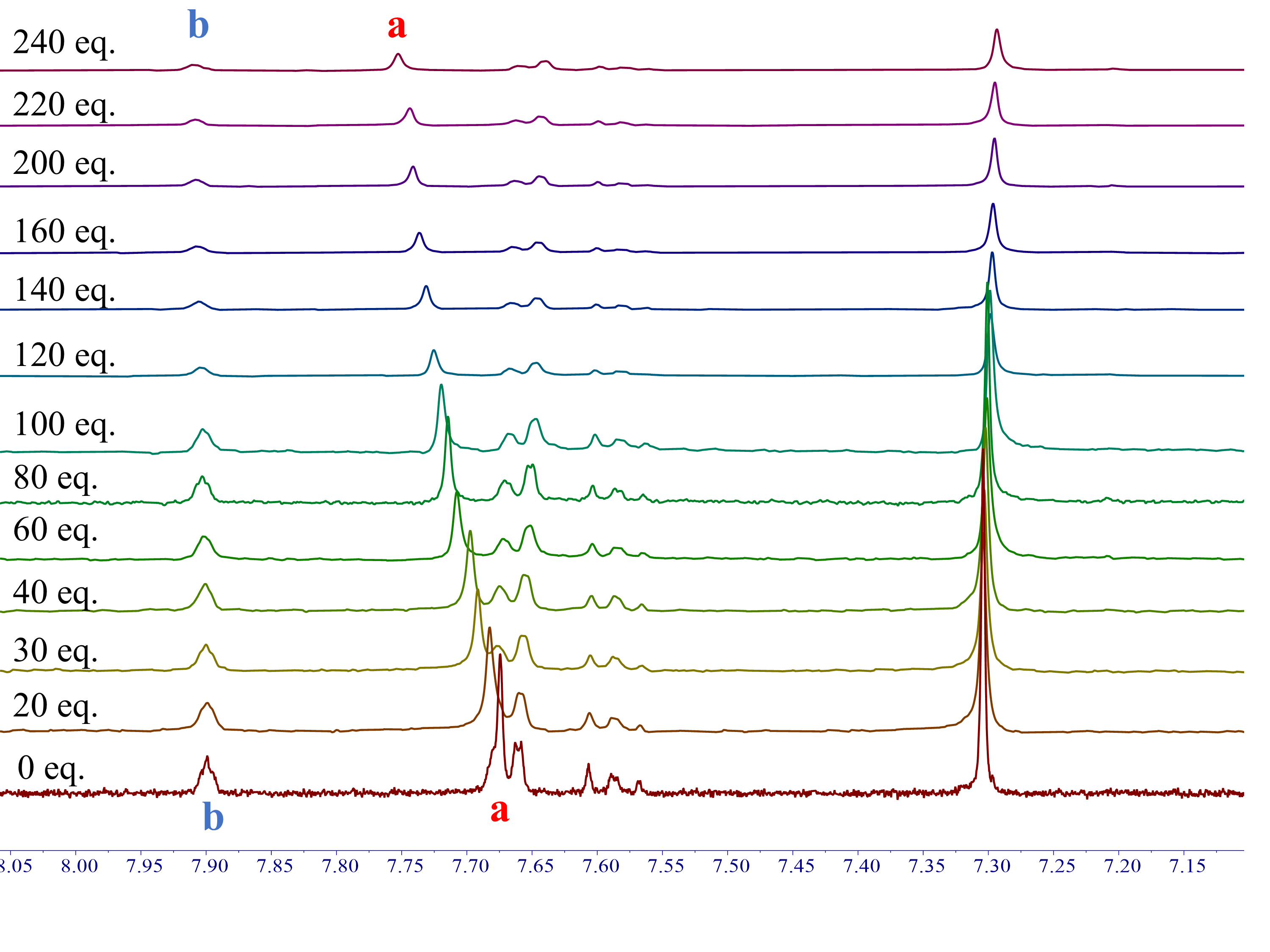


Figure S73: Stacked spectra for the ^1^H NMR titration (400 MHz, DMSO-*d6*) of compound **CH5** (3 mM) with the successive addition of TBANO3.


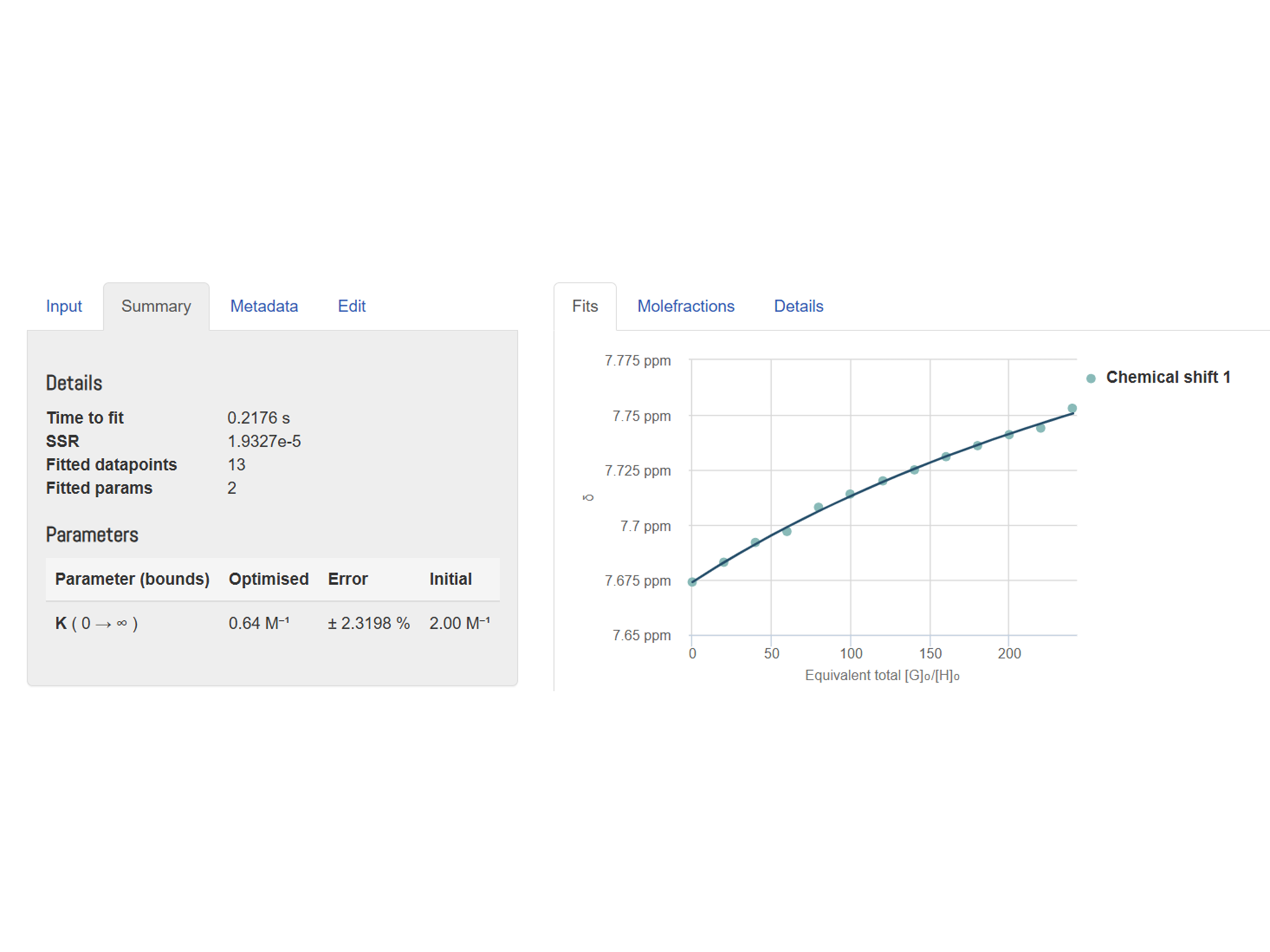


Figure S74: Screenshot of the fitted data plot from supramolecular.org for the titration of **CH5** with TBANO3. The binding constant was found to be 0.64 M^-1^ ± 2.0% in 1:1 receptor to anion binding model (left side). The changing pattern of chemical shift with the increasing equivalent TBABr (right side). The Bindfit URL for this experiment is: <http://app.supramolecular.org/bindfit/view/4ea74d6f-7996-4d74-b340-adc848815c90>

# DFT calculations

DFT calculations were performed using the Gaussian 16 package,^[10]^ employing the B3LYP hybrid functional^[11,12]^ in conjunction with the Def2-TZVP basis set.^[13]^ One must mention that Grimme's empirical dispersion D3 was implemented.^[14]^ All the geometries were optimised in gas phase, water (to mimic the intra- and extravesicular space) and a medium with a dielectric constant of 2 (to mimic the membrane hydrophobic medium) using the PCM solvation model^[15]^ and without any symmetry constraints. Moreover, frequency calculations have been performed to characterise the nature of the stationary points (i.e., ground state or transition state) and to determine the zero-point energy, as well as the thermal corrections. All optimised geometries show zero imaginary frequency and correspond to minima on the potential energy surface. Nevertheless, within all calculations, the integration grid was set to ultrafine.


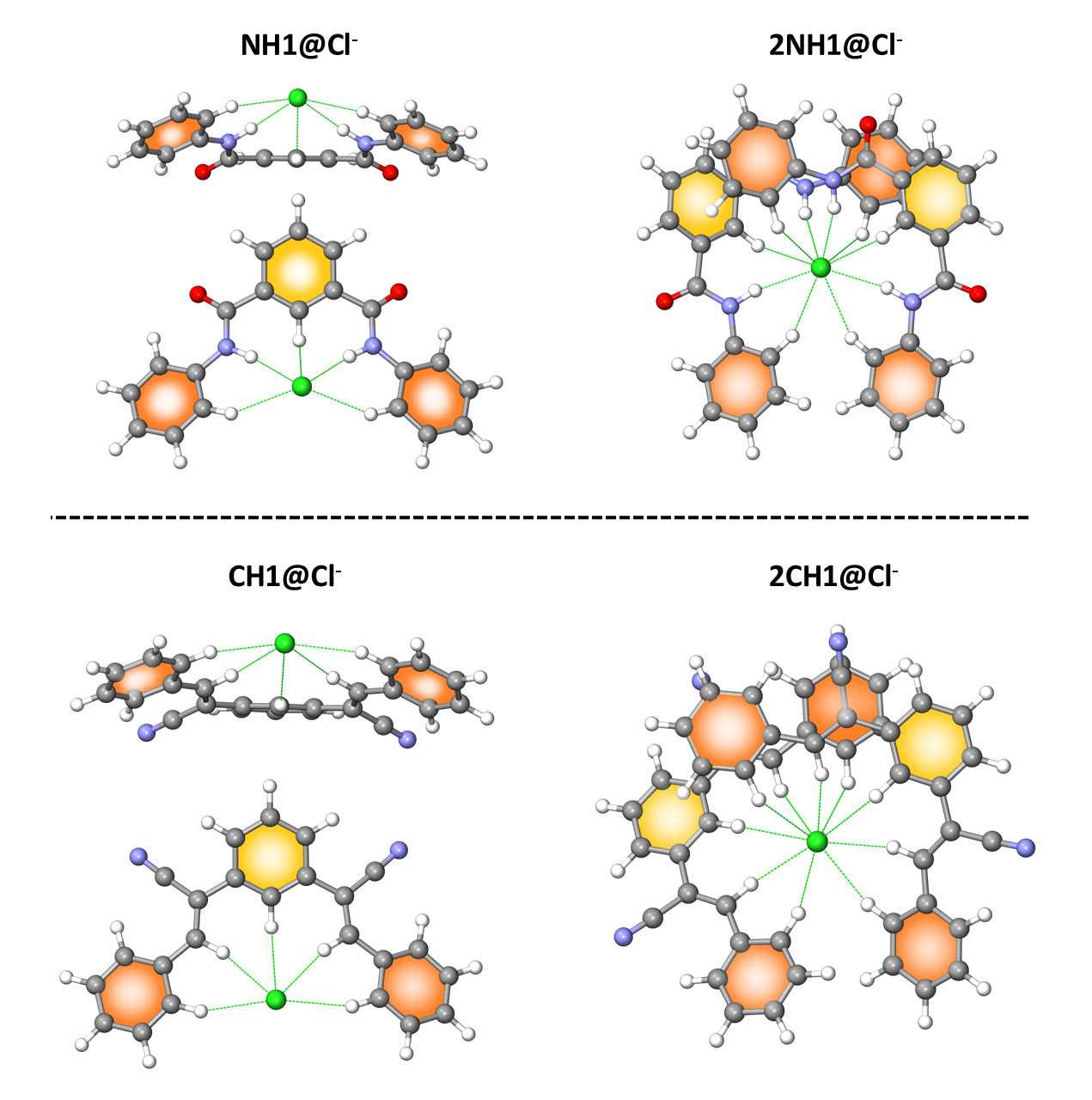


Figure S75. Optimized geometries of **[NH1@Cl]^−^**, **[2NH1@Cl]^−^**, **[CH1@Cl]^−^**, and **[2CH1@Cl]^−^.**

The distribution of the electrostatic potential (ESP) surface was computed for the optimized free-anion **CH** derivatives in gas phase at the same level of theory. The quantitative analysis of the molecular surface has been performed using Multiwfn.^[16,17]^


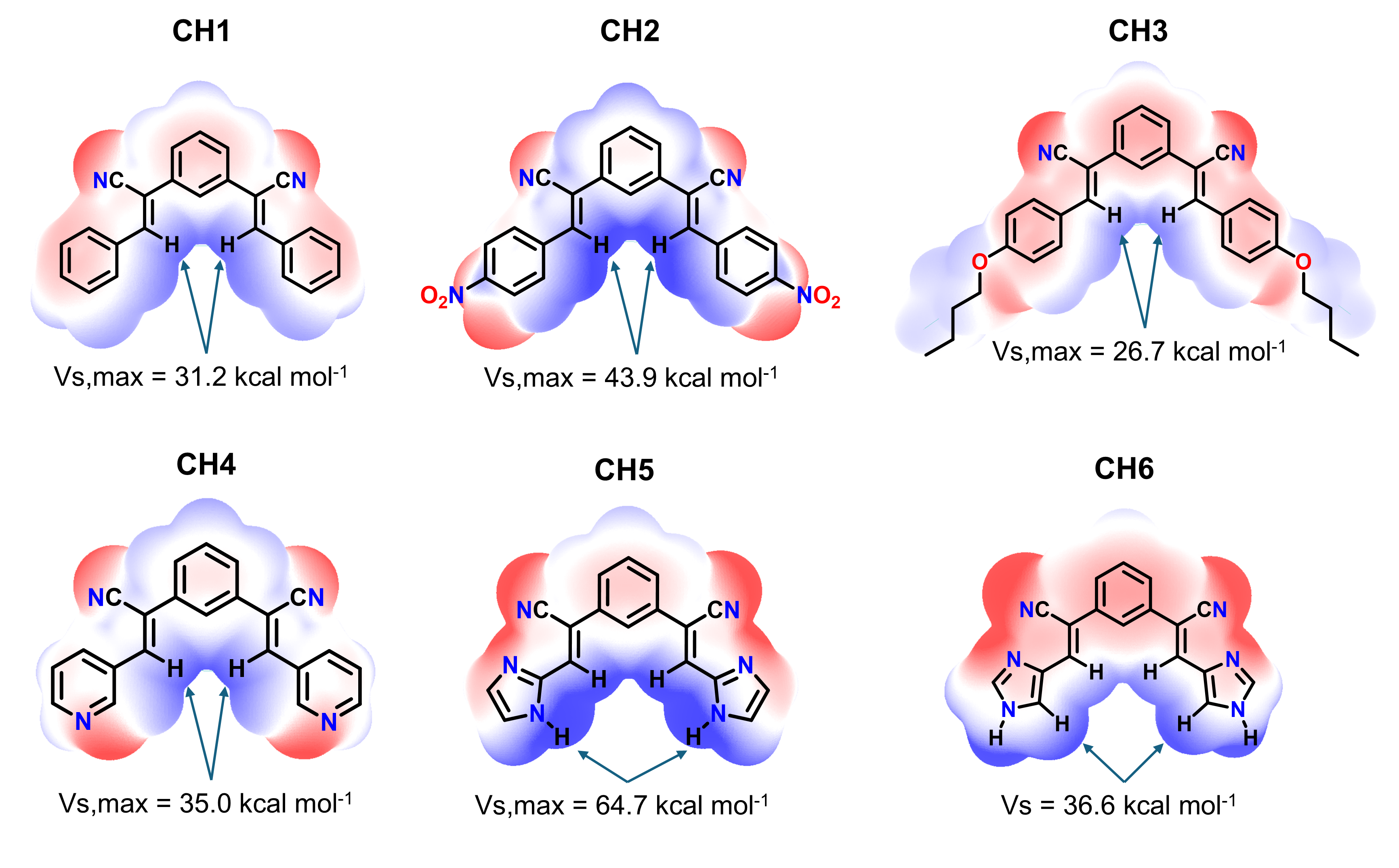


Figure S76. The distribution of the electrostatic potential (ESP) surface and the calculated maxima points (Vs,max kcal mol^-1^) for the studied **CH** derivatives.

The strength of the CH•••Anion interaction was evaluated using the Quantum Theory of Atoms in Molecules (QTAIM) formalism.^[18,19]^ The energy of the hydrogen bonding interaction (*E_HB_*) was estimated in the hydrophobic membrane medium (ε = 2) from the potential energy density (ν) using the Espinosa relation (11).^[20]^ Moreover, the Laplacian of the electron density (∇^2^ρ) was found positive for all CH•••Anion interactions, indicating a depletion of the electron distribution consistent with the formation of the hydrogen bonding interactions.

$$E_{HB}\approx\frac{1}{2}\nu(11)$$

Table S2. Non-bonding interaction energies of **CH3** with chloride, bromide and nitrate estimated using Espinosa relationship based on the QTAIM analysis on the optimized 1:1 complexes at B3LYP-D3/def2TZVP level of theory in the hydrophobic membrane medium (ε = 2).

| **Transp.** | **Anion** | ***E*(Ha···X^-^)**  **(kJ mol^-1^)** | ***E*(Hb···X^-^)**  **(kJ mol^-1^)** | ***E*(Hc···X^-^)**  **(kJ mol^-1^)** |
| --- | --- | --- | --- | --- |
| **CH3** | Cl^-^ | 8.53 | -6.98 | -4.47 |
|  | Br^-^ | -6.56 | -6.70 | -1.53 |
|  | NO_3_^-^ | -10.15 | -9.33 | -5.41 |

Table S3. Hydrogen bond distances and angles for Ha···X^-^ (average values with standard deviations in parentheses), Hb···X^-^ (average values with standard deviations in parentheses), and Hc···X^-^ interactions (as labelled in Figure 9 in the main text) for the 1:1 complexes of CH donors with chloride, bromide and nitrate optimized at B3LYP-D3/def2TZVP level of theory in a hydrophobic medium with ε = 2.

| **Transp.** | **Anion** | **d (Å)**  **Ha···X^-^** | **d (Å)**  **Hb···X^-^** | **d (Å)**  **Hc···X^-^** | **∠ (°)**  **CHa···X^-^** | **∠ (°)**  **CHb···X^-^** | **∠ (°)**  **CHc···X^-^** |
| --- | --- | --- | --- | --- | --- | --- | --- |
| **CH1** | Cl^-^ | 2.530 (0.006) | 2.648 (0.026) | 2.949 | 166.51 (0.17) | 155.21 (1.21) | 135.52 |
|  | Br^-^ | 2.737 (0.013) | 2.824 (0.021) | 3.308 | 165.53 (0.01) | 156.90 (0.09) | 125.07 |
|  | NO_3_^-^ | 2.261 (0.007) | 2.290 (0.007) | 2.542 | 165.25 (1.33) | 164.19 (3.63) | 135.80 |
| **CH2** | Cl^-^ | 2.498 (0.016) | 2.586 (0.003) | 2.711 | 160.50 (0.42) | 154.35 (0.88) | 157.72 |
|  | Br^-^ | 2.646 (0.003) | 2.804 (0.004) | 3.157 | 166.41 (0.18) | 154.14 (0.03) | 128.18 |
|  | NO_3_^-^ | 2.230 (0.015) | 2.278 (0.022) | 2.498 | 165.83 (1.09) | 158.75 (0.68) | 137.24 |
| **CH3** | Cl^-^ | 2.551 (0.001) | 2.650 (0.016) | 2.946 | 165.57 (0.34) | 155.21 (0.74) | 138.38 |
|  | Br^-^ | 2.772 (0.018) | 2.757 (0.005) | 3.248 | 164.27 (0.60) | 159.01 (1.69) | 134.33 |
|  | NO_3_^-^ | 2.267 (0.005) | 2.266 (0.024) | 2.574 | 165.93 (1.57) | 166.36 (1.79) | 143.83 |
| **CH4** | Cl^-^ | 2.461 (0.000) | 2.696 (0.005) | 2.789 | 167.77 (0.58) | 151.28 (0.21) | 141.69 |
|  | Br^-^ | 2.646 (0.010) | 2.882 (0.003) | 3.3.163 | 168.31 (0.36) | 153.42 (0.64) | 124.68 |
|  | NO_3_^-^ | 2.230 (0.013) | 2.453 (0.034) | 2.513 | 167.09 (0.36) | 148.21 (0.09) | 134.20 |
| **CH5** | Cl^-^ | 2.734 (0.011) | 2.243 (0.004) | 3.378 | 147.68 (0.21) | 161.71 (0.52) | 142.14 |
|  | Br^-^ | 2.903 (0.016) | 2.438 (0.003) | 3.530 | 149.02 (0.61) | 162.13 (0.52) | 142.74 |
|  | NO_3_^-^ | 2.267 (0.006) | 1.841 (0.001) | 2.530 | 153.77 (0.14) | 172.91 (0.05) | 151.87 |
| **CH6** | Cl^-^ | 2.648 (0.000) | 2.655 (0.014) | 3.125 | 156.27 (0.65) | 140.66 (0.17) | 148.88 |
|  | Br^-^ | 2.860 (0.021) | 2.817 (0.009) | 3.352 | 155.69 (0.40) | 142.34 (0.21) | 148.43 |
|  | NO_3_^-^ | 2.369 (0.009) | 2.105 (0.011) | 2.732 | 162.01 (1.47) | 176.82 (0.05) | 145.34 |

Figure S77. Calculated H_2_O and NO_3_^−^ binding enthalpies of **CH1**, **CH2** and **CH4** relative to **CH3** at B3LYP-D3/Def2-TZVP level of theory.

# References

[1] Bruker, Bruker AXS Inc., Madison, Wisconsin, USA **2018**.

[2] L. Palatinus, G. Chapuis, “SUPERFLIP – a computer program for the solution of crystal structures by charge flipping in arbitrary dimensions” *J. Appl. Crystallogr.* **2007**, *40*, 786–790.

[3] A. Van Der Lee, “Charge flipping for routine structure solution” *J. Appl. Crystallogr.* **2013**, *46*, 1306–1315.

[4] P. W. Betteridge, J. R. Carruthers, R. I. Cooper, K. Prout, D. J. Watkin, “CRYSTALS version 12: software for guided crystal structure analysis” *J. Appl. Crystallogr.* **2003**, *36*, 1487–1487.

[5] K. Ozvoldik, T. Stockner, E. Krieger, “YASARA Model–Interactive Molecular Modeling from Two Dimensions to Virtual Realities” *J. Chem. Inf. Model.* **2023**, *63*, 6177–6182.

[6] E. Krieger, T. Darden, S. B. Nabuurs, A. Finkelstein, G. Vriend, “Making optimal use of empirical energy functions: Force-field parameterization in crystal space” *Proteins Struct. Funct. Bioinforma.* **2004**, *57*, 678–683.

[7] E. Krieger, G. Vriend, “New ways to boost molecular dynamics simulations” *J. Comput. Chem.* **2015**, *36*, 996–1007.

[8] P. Thordarson, “Determining association constants from titration experiments in supramolecular chemistry” *Chem. Soc. Rev.* **2011**, *40*, 1305–1323.

[9] D. Brynn Hibbert, P. Thordarson, “The death of the Job plot, transparency, open science and online tools, uncertainty estimation methods and other developments in supramolecular chemistry data analysis” *Chem. Commun.* **2016**, *52*, 12792–12805.

[10] M. J. Frisch, et al., *Gaussian 16*, Gaussian, Inc., Wallingford, CT, USA, **2016**.

[11] A. D. Becke, “Density‐functional thermochemistry. III. The role of exact exchange” *J. Chem. Phys.* **1993**, *98*, 5648–5652.

[12] C. Lee, W. Yang, R. G. Parr, “Development of the Colle-Salvetti correlation-energy formula into a functional of the electron density” *Phys. Rev. B* **1988**, *37*, 785–789.

[13] F. Weigend, R. Ahlrichs, “Balanced basis sets of split valence, triple zeta valence and quadruple zeta valence quality for H to Rn: Design and assessment of accuracy” *Phys. Chem. Chem. Phys.* **2005**, *7*, 3297–3305.

[14] S. Grimme, J. Antony, S. Ehrlich, H. Krieg, “A consistent and accurate ab initio parametrization of density functional dispersion correction (DFT-D) for the 94 elements H-Pu” *J. Chem. Phys.* **2010**, *132*, 154104.

[15] J. Tomasi, B. Mennucci, R. Cammi, “Quantum Mechanical Continuum Solvation Models” *Chem. Rev.* **2005**, *105*, 2999–3094.

[16] T. Lu, F. Chen, “Quantitative analysis of molecular surface based on improved Marching Tetrahedra algorithm” *J. Mol. Graph. Model.* **2012**, *38*, 314–323.

[17] T. Lu, F. Chen, “Multiwfn: A multifunctional wavefunction analyzer” *J. Comput. Chem.* **2012**, *33*, 580–592.

[18] R. F. W. Bader, “A quantum theory of molecular structure and its applications” *Chem. Rev.* **1991**, *91*, 893–928.

[19] R. F. W. Bader, R. F. W. Bader, *Atoms in Molecules: A Quantum Theory*, Oxford University Press, Oxford, New York, **1994**.

[20] E. Espinosa, E. Molins, C. Lecomte, “Hydrogen bond strengths revealed by topological analyses of experimentally observed electron densities” *Chem. Phys. Lett.* **1998**, *285*, 170–173.
